# Exploring the Chemodiversity and Biological Activities of the Secondary Metabolites from the Marine Fungus *Neosartorya pseudofischeri*

**DOI:** 10.3390/md12115657

**Published:** 2014-11-24

**Authors:** Wan-Ling Liang, Xiu Le, Hou-Jin Li, Xiang-Ling Yang, Jun-Xiong Chen, Jun Xu, Huan-Liang Liu, Lai-You Wang, Kun-Teng Wang, Kun-Chao Hu, De-Po Yang, Wen-Jian Lan

**Affiliations:** 1 School of Pharmaceutical Sciences, Sun Yat-sen University, Guangzhou 510006, China; E-Mails: natprodlwl@gmail.com (W.-L.L.); lexiu2012@163.com (X.L.); junxu@biochemomes.com (J.X.); kunchao89@gmail.com (K.-C.H.); lssydp@mail.sysu.edu.cn (D.-P.Y.); 2 Guangdong Technology Research Center for Advanced Chinese Medicine, Guangzhou 510006, China; 3 School of Chemistry and Chemical Engineering, Sun Yat-sen University, Guangzhou 510275, China; E-Mail: ceslhj@mail.sysu.edu.cn; 4 Guangdong Institute of Gastroenterology, Guangzhou 510655, China; E-Mails: morningyang100@gmail.com (X.-L.Y.); junxiong_ch@sina.com (J.-X.C.); huanliang.liu@gmail.com (H.-L.L.); 5 Guangdong Key Laboratory of Colorectal and Pelvic Floor Diseases, Guangzhou 510655, China; 6 The Sixth Affiliated Hospital, Sun Yat-sen University, Guangzhou 510655, China; 7 Institute of Chinese Medical Sciences, Guangdong Pharmaceutical University, Guangzhou 510006, China; E-Mails: wanglaiyou@gdpu.edu.cn (L.-Y.W.); kuntengwang@gmail.com (K.-T.W.)

**Keywords:** marine fungus, *Neosartorya pseudofischeri*, neosartin, diketopiperazine, antibacterial activity, cytotoxic activity

## Abstract

The production of fungal metabolites can be remarkably influenced by various cultivation parameters. To explore the biosynthetic potentials of the marine fungus, *Neosartorya pseudofischeri*, which was isolated from the inner tissue of starfish *Acanthaster planci*, glycerol-peptone-yeast extract (GlyPY) and glucose-peptone-yeast extract (GluPY) media were used to culture this fungus. When cultured in GlyPY medium, this fungus produced two novel diketopiperazines, neosartins A and B (**1** and **2**), together with six biogenetically-related known diketopiperazines,1,2,3,4-tetrahydro-2,3-dimethyl-1,4-dioxopyrazino[1,2-a]indole (**3**), 1,2,3,4-tetrahydro-2-methyl-3-methylene-1,4-dioxopyrazino[1,2-a]indole (**4**), 1,2,3,4-tetrahydro-2-methyl-1,3,4-trioxopyrazino[1,2-a] indole (**5**), 6-acetylbis(methylthio)gliotoxin (**10**), bisdethiobis(methylthio)gliotoxin (**11**), didehydrobisdethiobis(methylthio)gliotoxin (**12**) and *N*-methyl-1*H*-indole-2-carboxamide (**6**). However, a novel tetracyclic-fused alkaloid, neosartin C (**14**), a meroterpenoid, pyripyropene A (**15**), gliotoxin (**7**) and five known gliotoxin analogues, acetylgliotoxin (**8**), reduced gliotoxin (**9**), 6-acetylbis(methylthio)gliotoxin (**10**), bisdethiobis(methylthio) gliotoxin (**11**) and bis-*N*-norgliovictin (**13**), were obtained when grown in glucose-containing medium (GluPY medium). This is the first report of compounds **3**, **4**, **6**, **9**, **10** and **12** as naturally occurring. Their structures were determined mainly by MS, 1D and 2D NMR data. The possible biosynthetic pathways of gliotoxin-related analogues and neosartin C were proposed. The antibacterial activity of compounds **2**–**14** and the cytotoxic activity of compounds **4**, **5** and **7**–**13** were evaluated. Their structure-activity relationships are also preliminarily discussed.

## 1. Introduction

Marine organism, such as sponges, soft corals and invertebrates, host numerous fungi. These marine fungi are an important source of structurally unique and biologically active natural products. Recent studies revealed that the cryptic biosynthetic pathways of fungi can be activated and the chemical diversity of their metabolites can be maximized by alternating their cultivation parameters systematically, such as the components of the media [1,2], co-culture [3,4], feeding precursors [5,6] and the addition of enzyme inhibitors [7,8]. For example, marine fungus *Chondrostereum* sp., which was isolated from soft coral *Sarcophyton tortuosum*, grows in different culture media and can produce various novel bioactive hirsutane-type sesquiterpenoids [9,10,11,12].

**Figure 1:**
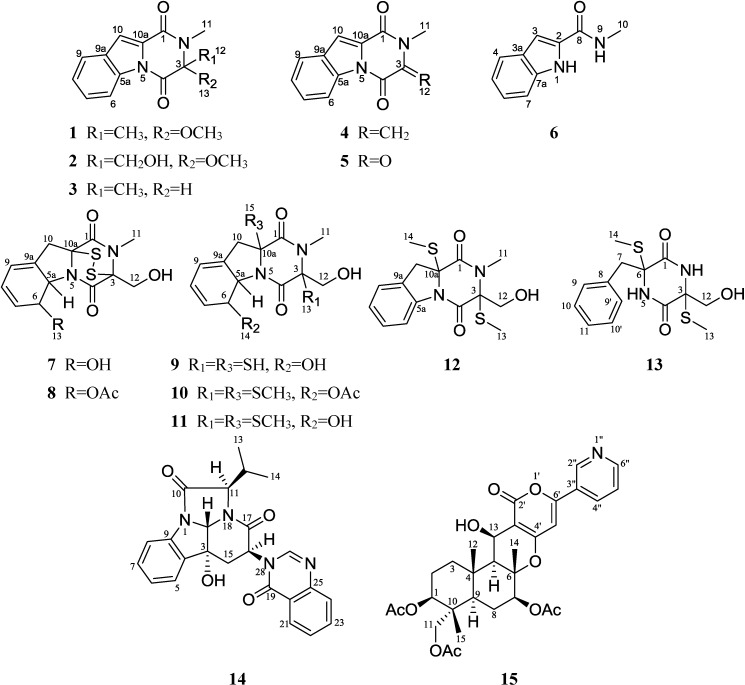
Structures of compounds **1**–**15**.

In recent years, we conducted research on the metabolites of marine fungi isolated from starfish *Acanthaster planci* and obtained a series of novel and/or bioactive metabolites [13,14,15,16]. In the current work, a marine fungus, *Neosartorya pseudofischeri*, was isolated from the inner tissue of *Acanthaster planci*. *Neosartorya* is a sexual state of *Aspergillus* section *Fumigati* [17]; however, unlike *Aspergillus*, the reports on the secondary metabolites of *Neosartorya* sp. have been relatively rare. The limited literature showed that most of the metabolites from *Neosartorya* species were cytotoxic nitrogenous-containing compounds [18,19]. In an attempt to explore the biosynthetic potentials, GlyPY (glycerol 10 g, peptone 5 g, yeast extract 2 g, CaCO_3_ 1 g, sea water 1 L) and GluPY (glucose 10 g, peptone 5 g, yeast extract 2 g, sea water 1 L, pH 7.5) media were separately used to culture the fungus, *Neosartorya pseudofischeri*. Both of the EtOAc extracts of two different culture conditions showed potent cytotoxicity against cancer cell line HCT-116 with the IC_50_ values lower than 20 μg/mL. The HPLC traces of these two EtOAc extracts also displayed distinct components and content differences (Supplementary Figure S1). Purification of the extract of GlyPY medium afforded two novel diketopiperazines, neosartins A and B (**1** and **2**), together with six biogenetically-related known diketopiperazines, 1,2,3,4-tetrahydro-2,3-dimethyl-1,4-dioxopyrazino[1,2-a]indole (**3**), 1,2,3,4-tetrahydro-2-methyl-3-methylene-1,4-dioxopyrazino[1,2-a]indole (**4**), 1,2,3,4-tetrahydro-2-methyl-1,3,4-trioxopyrazino[1,2-a]indole (**5**), 6-acetylbis(methylthio) gliotoxin (**10**), bisdethiobis (methylthio)gliotoxin (**11**), didehydrobisdethiobis(methy1thio)gliotoxin (**12**) and *N*-methyl-1*H*-indole-2-carboxamide (**6**). Isolation of the extract of GluPY medium gave a new alkaloid, neosartin C (**14**), known compounds, α-pyrone meroterpenoid pyripyropene A (**15**), gliotoxin (**7**) and gliotoxin analogues, acetylgliotoxin (**8**), reduced gliotoxin (**9**), 6-acetylbis(methylthio)gliotoxin (**10**), bisdethiobis(methylthio)gliotoxin (**11**) and bis-*N*-norgliovictin (**13**) (Figure 1). In this paper, we report the isolation, structural elucidation, proposed biosynthetic pathways, bioactivities and structure-activity relationships of these compounds.

## 2. Results and Discussion

### 2.1. Structural Elucidation

**Figure 2:**
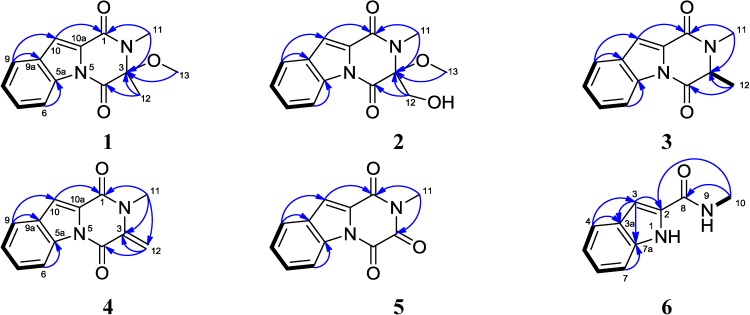
^1^H-^1^H COSY (bold line) and the main HMBC (arrows) correlations of compounds **1**–**6**.

Compound **1** was obtained as a yellowish solid. The molecular formula was determined as C_14_H_14_N_2_O_3_ from the HREIMS peak at *m/z* 258.0998 [M]^+^ (calcd. 258.0999) (Supplementary Figures S2 and S3), implying nine degrees of unsaturation. The IR spectrum indicated the presence of a carbonyl group (1680 cm^−1^) and a benzene ring (3073, 1574 and 1507 cm^−1^). UV maxima at 211, 243 and 295 nm displayed the conjugated system containing a benzene ring. The ^13^C NMR and DEPT spectra displayed three methyls, five methines and six quaternary carbons (Table 1 and Supplementary Figures S4–S6). Two quaternary carbons at δ_C_ 163.7 and 156.7 are amide carbonyls. The amide protons were substituted for the lack of the corresponding signals in IR and ^1^H NMR spectra in CDCl_3_. Eight carbon resonance signals appeared in the region of δ_C_ 114.9~134.8. Among them, four aromatic methines (δ_C_ 116.8, δ_H_ 8.50 (d, 8.0, H-6); δ_C_ 125.8, δ_H_ 7.43 (ddd, 8.0, 8.0, 0.8, H-7); δ_C_ 128.2, δ_H_ 7.55 (ddd, 8.0, 8.0, 0.8, H-8); and δ_C_ 122.7, δ_H_ 7.72 (d, 8.0, H-9)) established the partial structure -CH-CH-CH-CH- based on the ^1^H-^1^H COSY correlations of H-6/H-7, H-7/H-8 and H-8/H-9 (Figure 2, Supplementary Figure S7). Further analysis on the HMBC correlations of H-6/C-5a and H-9/C-9a suggested a 1,2-disubstutited benzene ring in the molecule. The methine (δ_C_ 114.9, δ_H_ 7.49, s) and a quaternary carbon C-10a at δ_C_ 127.8 constructed an additional trisubstituted double bond. The HMBC correlations of H-9/C-10, H-10/C-9a, H-10/C-10a and H-10/C-1 revealed the trisubstituted double bond connected to a benzene ring and an amide carbonyl. Methyl group C-11 at δ_H_ 3.13 (δ_C_ 26.6) showed HMBC correlations with amide carbonyl C-1 and quaternary carbon C-3, so it was connected to amide nitrogen N-2. Methyl group C-12 at δ_H_ 1.81 (δ_C_ 25.3) was connected to C-3 based on the HMBC correlation with the amide carbon C-4 and quaternary carbon C-3. The methoxyl group was also located at C-3, because it showed an HMBC correlation with C-3 (Supplementary Figure S8). Finally, in order to connect the remaining open bonds, C-5a must be linked to the nitrogen atom at the 5-position to form the additional five-membered ring. In the NOESY spectrum, the correlations among the protons of three methyl groups and H-9/H-10, confirmed the connection (Supplementary Figure S9). Therefore, compound **1** was established as 1,2,3,4-tetrahydro-3-methoxyl-2,3-dimethyl-1,4-dioxopyrazino[1,2-a]indole, trivially named neosartin A.

Compound **2** was isolated as a yellowish solid. The molecular formula was established as C_14_H_14_N_2_O_4_ based on the HREIMS peak at *m/z* 274.0947 [M]^+^ and ^13^C NMR data (Table 1, and Supplementary Figures S11 and S13). The ^13^C NMR and DEPT spectra displayed two methyls, one methylene, five methines and six quaternary carbons. The NMR data of compound **2** were very similar to those of compound **1** (Figure 2 and Supplementary Figures S12–S17). By comparison of their NMR data, a quick identification was made that the methyl C-12 (δ_C_ 25.3, δ_H_ 1.81, s) in **1** was replaced by an oxymethylene (δ_C_ 64.9, δ_H_ 4.18, d, *J* = 10.7 Hz; 3.99, d, *J* = 10.7 Hz) in **2**. Therefore, the structure of **2** was elucidated as 1,2,3,4-tetrahydro-3-methoxyl-3-hydroxylmethyl-2-methyl-1,4-dioxopyrazino[1,2-a]indole, commonly named neosartin B.

Compounds **1** and **2** did not show optical activity in circular dichroism (CD) spectra; thus, they existed as a racemic mixture of 3*R* and 3*S*.

Compound **3** was isolated as a white solid. The molecular formula was established as C_13_H_12_N_2_O_2_ based on the LREIMS molecular ion at *m/z* 228 and the NMR data (Table 1 and Supplementary Figure S18). Its NMR spectra data closely resembled those of **1** except for the methoxy group in **1**, which was replaced with a hydrogen atom in **3**, and that correlated with C-3 in the HMBC spectrum. Due to the vicinal coupling with methyl group C-12, the ^1^H signal at δ 4.33 appeared as a typical quartet with *J* = 7.2 Hz. The structure of **3** was confirmed by ^1^H-^1^H COSY, HMBC and NOESY data (Supplementary Figures S19–S24). Compound **3** was determined to be 1,2,3,4-tetrahydro-2,3-dimethyl-1,4-dioxopyrazino[1,2-a]indole. It was once synthesized by heating anhydrodethiogliotoxin with acetic anhydride [20]; however, this is the first time that the detailed NMR data have been presented.

**Table 1:** ^1^H and ^13^C NMR data of compounds **1**–**3** at 400/100 MHz, respectively, in CDCl_3_, δ in ppm.

Position	1			2			3		
	δ_C_	Type	δ_H_, mult., (*J* in Hz)	δ_C_	Type	δ_H_, mult., (*J* in Hz)	δ_C_	Type	δ_H_, mult., (*J* in Hz)
1	156.7	C		158.5	C		156.2	C	
2		N			N			N	
3	90.7	C		93.6	C		60.2	CH	4.33, q (7.2)
4	163.7	C		162.9	C		165.5	C	
5		N			N			N	
5a	129.2	C		128.8	C		129.2	C	
6	116.9	CH	8.49, dd (8.0, 0.8)	116.6	CH	8.47, dd (7.6, 0.8)	116.5	CH	8.43, d (8.0)
7	128.2	CH	7.55, ddd (8.0, 8.0, 0.8)	128.1	CH	7.49, ddd (7.6,7.6, 0.8)	127.8	CH	7.51, dd (8.0, 8.0)
8	125.8	CH	7.43, ddd (8.0, 8.0, 0.8)	125.7	CH	7.26, ddd (7.6, 7.6, 0.8)	125.4	CH	7.40, dd (8.0, 8.0)
9	122.7	CH	7.72, dd (8.0, 0.8)	122.5	CH	7.32, dd (7.6, 0.8)	122.5	CH	7.70, d (8.0)
9a	134.8	C		134.5	C		134.8	C	
10	114.9	CH	7.50, s	115.0	CH	7.19, s	114.1	CH	7.44, s
10a	127.8	C		127.5	C		128.5	C	
11	26.6	CH_3_	3.13, s	26.5	CH_3_	3.14, s	31.8	CH_3_	3.16, s
12	25.3	CH_3_	1.81, s	64.9	CH_2_	4.17, d (11.6);4.02, d (11.6)	19.8	CH_3_	1.71, d (7.2)
13	52.0	O*CH_3_*	3.20, s	52.0	O*CH_3_*	3.24, s			
12-OH						2.04, brs			

Compound **4** contains a typical terminal C=C double bond (δ_C_ 137.7, C-3; δ_C_ 106.0, δ_H_ 6.15, s; 5.25, s, C-12). Its structure was elucidated as 1,2,3,4-tetrahydro-2-methyl-3-methylene-1,4-dioxopyrazino[1,2-a]indole by analysis of its spectral data (Table 2, Figure 2 and Supplementary Figures S25–S31). Compound **4** was previously obtained as the conversion product of gliotoxin (**7**) by passing through a column of alkaline alumina at 20 °C [21]. Compound **5** was deduced as 1,2,3,4-tetrahydro-2-methyl-1,3,4-trioxopyrazino[1,2-a]indole by careful analysis of the MS and NMR data (Table 2, Figure 2 and Supplementary Figures S32–S38). Compound **5** was firstly reported in 1945 as a degradation product of gliotoxin by heating with selenium [22]. It was also isolated from the culture of *Penicillium terlikowskii* [23]. Compound **6** was obtained as a white solid. The molecular formula was established as C_10_H_10_N_2_O on the basis of HREIMS (*m*/*z* 174.0789 [M]^+^, calcd. 174.0788) and NMR data (Table 2 and Supplementary Figures S39–S45). The structure of **6** was elucidated as *N*-methyl-1*H*-indole-2-carboxamide by analysis on the 1D and 2D NMR (HMQC, HMBC and ^1^H-^1^H COSY) (Table 2 and Figure 2). It is unprecedented that compound **6** was obtained from a natural source.

**Table 2:** ^1^H and ^13^C NMR data of compounds **4**–**6** at 400/100 MHz, respectively, δ in ppm.

Position	4 ^a^			5 ^b^			Position	6 ^c^		
	δ_C_	Type	δ_H_, mult., (*J* in Hz)	δ_C_	Type	δ_H_, mult., (*J* in Hz)		δ_C_	Type	δ_H_, mult., (*J* in Hz)
1	154.4	C		157.0	C		1		NH	11.04, brs
2		N			N		2	132.8	C	
3	137.7	C		156.8	C		3	102.7	CH	7.07, s
4	154.9	C		149.9	C		3a	137.8	C	
5		N			N		4	122.5	CH	7.61, d (8.0)
5a	128.9	C		128.3	C		5	124.5	CH	7.22, dd (8.0, 8.0)
6	117.0	CH	8.50, d (8.0)	115.8	CH	8.32, d (8.0)	6	120.9	CH	7.06, dd (8.0, 8.0)
7	128.2	CH	7.53, dd (8.0, 8.0)	129.0	CH	7.63, d (8.0, 8.0)	7	113.2	CH	7.58, d (8.0)
8	125.5	CH	7.40, dd (8.0, 8.0)	125.7	CH	7.47, d (8.0, 8.0)	7a	128.9	C	
9	122.8	CH	7.72, d (8.0)	123.9	CH	7.88, d (8.0)	8	163.0	C	
9a	135.6	C		135.4	C		9		NH	7.83, brs
10	115.3	CH	7.51, s	116.3	CH	7.72, s	10	26.4	CH_3_	2.97, s
10a	127.6	C		127.7	C					
11	29.6	CH_3_	3.41, s	26.6	CH_3_	3.22, s				
12	106.0	CH_2_	6.15, s; 5.25, s							

^a^ Measured in CDCl_3_. ^b^ Measured in DMSO-*d*_6_. ^c^ Measured in acetone-*d*_6_.

Compound **7** was identified as gliotoxin by comparing the data with the literature values [24,25] (Supplementary Figures S46–S48). The NMR spectra data of compound **8** closely resembled those of **7**, except for one additional ^1^H resonance signal of the acetyl group. Its structure was identified as acetylgliotoxin, which was isolated from fungus strain FO2047 previously, and showed broad activities, including inhibition of fungi, bacteria and viruses [26] (Table 3 and Supplementary Figures S49 and S50). Compound **9**, having NMR data similar to those of **7**, was identified as reduced gliotoxin, which was the reduced dithiol form of 7 (Table 3 and Supplementary Figures S51 and S52). Daniel *et al*. found that gliotoxin (**7**) was generated from the corresponding dithiol (**9**) by a novel FAD-dependent dithiol oxidase, GliT [27]. Although **4**–**6**, **8** and **9** are known compounds, their detailed NMR data were never reported previously.

**Table 3:** ^1^H and ^13^C NMR data of compounds **8** and **9** in CDCl_3_, δ in ppm.

Position	8 ^a^			9 ^b^		
	δ_C_	Type	δ_H_, mult., (*J* in Hz)	δ_C_	Type	δ_H_, mult., (*J* in Hz)
1	168.4	C		169.7	C	
2		N			N	
3	78.2	C		78.6	C	
4	165.9	C		168.1	C	
5		N			N	
5a	64.9	CH	5.36, d (14.1)	70.6	CH	4.77, d (13.2)
6	74.3	CH	5.82, d (14.1)	72.7	CH	5.04, d (13.2)
7	128.6	CH	5.93, m	129.8	CH	5.93, m
8	124.9	CH	5.93, m	123.1	CH	5.88, m
9	120.7	CH	5.60, d (7.5)	120.7	CH	5.75, m
9a	131.7	C		130.0	C	
10	41.4	CH_2_	3.25, d (15.0);		CH_2_	3.28, d (16.2);
			3.08, d (15.0)			3.05, d (16.2)
10a	77.6	C		77.2	C	
11	29.0	CH_3_	3.10, s	28.9	CH_3_	3.15, s
12	62.4	CH_2_	4.32, d (12.3)	62.3	CH_2_	4.39, d (12.0)
			4.00, d (12.3)			4.06, d (12.0)
13	170.1	*CO*CH_3_				
	21.4	CO*CH_3_*	2.17, s			

^a^ ^1^H and ^13^C NMR data were measured at 300/75 MHz; ^b^ ^1^H and ^13^C NMR data were measured at 400/100 MHz. CDCl_3_.

Compounds **10**–**13** were identified as 6-acetylbis(methylthio)gliotoxin (**10**) [28], bisdethiobis(methylthio)gliotoxin (**11**) [29], didehydrobisdethiobis(methylthio)gliotoxin (**12**) [30] and bis-*N*-norgliovictin (**13**) [31], respectively, by comparing their spectroscopic data (Table 4 and Supplementary Figures S53–S60) with the literature values. In the literature, only the ^1^H NMR data of compound **12** were reported; here, we report the detailed ^1^H and ^13^C NMR data. The ^13^C NMR data of compound **13** recorded in DMSO-*d*_6_ were shifted about 0.7~2.5 ppm to a higher field compared to the data reported for pyridine-*d*_5_.

**Figure 3:**
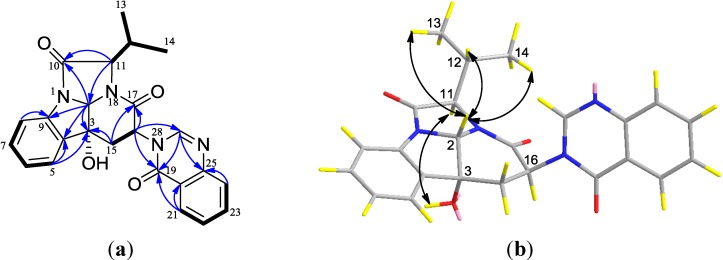
(**a**) ^1^H-^1^H COSY (bold line), the main HMBC (arrows); and (**b**) key NOESY correlations of compound **14**.

Compound **14** was obtained as a yellow solid. The molecular formula was deduced as C_24_H_22_N_4_O_4_ from the HREIMS peak at *m*/*z* 430.1635 [M]^+^ (calcd. 430.1636), implying 16 degrees of unsaturation (Supplementary Figure S62). The ^13^C NMR and DEPT spectra displayed twenty-four carbons, which were classified into two methyls, one methylene, thirteen methines and eight quaternary carbons (Table 5, Supplementary Figures S63 and S64). The chemical shifts of sixteen carbons were located at δ_C_ 115.0–172.0, corresponding to the aromatic or double-bond carbons. The ^1^H NMR spectrum showed eight proton signals in the downfield region (δ_H_ 7.15~8.20) with the coupling constants being about 7.6, suggesting at least two phenyl groups in the molecule. By analysis of the HMQC spectrum, the ^1^H and ^13^C NMR data of each carbon were definitely assigned. The ^1^H-^1^H COSY correlations of H-15/H-16, H-11/H-12, H-12/H-13 and H-12/H-14 deduced the presence of two partial structures, -CH_2_CH- and -CHCH(CH_3_)_2_, respectively (Figure 3a, Supplementary Figure S66). Furthermore, the COSY correlations of H-5/H-6, H-6/H-7, H-7/H-8, H-21/H-22, H-22/H-23 and H-23/H-24 indicate that there are two -CHCHCHCH- in the molecule. HMBC correlations of H-5/C-4, H-6/C-4, H-7/C-9, H-8/C-9, H-21/C-20, H-21/C-25, H-22/C-20, H-23/C-25 and H-24/C-25 further confirmed the presence of two disubstituted benzene rings. Three quaternary carbons at δ_C_ 171.4 (C-10), 163.6 (C-17) and 161.7 (C-19) are amide carbonyl groups. HMBC correlations of H-11/C-10, H-16/C-17, H-21/C-19 revealed that three amide carbonyl groups were connected to -CHCH(CH_3_)_2_, -CH_2_CH- and a disubstituted benzene ring, respectively. The hydroxyl group at δ_H_ 4.09 (s) was attached to C-3 (δ_C_ 74.8), and C-3 was connected to the methine C-2 (δ_C_ 84.8) and C-15 (δ_C_ 36.5) based on the HMBC correlations of H-2/C-3 and H-15/C-3. The planar structure of **14** was finally established by the HMBC correlations of H-2/C-10, H-5/C-3, H-15/C-3, H-16/C-19, H-16/C-27 and H-27/C-25 (Supplementary Figure S67).

**Table 4:** ^1^H and ^13^C NMR data of compounds **12** and **13**, δ in ppm.

Position	12 ^a^			Position	13 ^b^		
	δ_C_	Type	δ_H_, mult., (*J* in Hz)		δ_C_	Type	δ_H_, mult., (*J* in Hz)
1	166.0	C		1	165.2	C	
2		N		2	NH	8.95, brs	
3	71.7	C		3	65.8	C	
4	161.8	C		4	165.0	C	
5		N		5		NH	8.40, brs
5a	128.9	C		6	65.6	C	
6	127.9	CH	8.03, d (8.0)	7	43.3	CH_2_	3.33, d (6.0); 3.31, d (6.0)
7	126.2	CH	7.31, dd (8.0, 8.0)	8	135.0	C	
8	125.2	CH	7.19, dd (8.0, 8.0)	9/9′	130.0	CH	7.20, m
9	118.1	CH	7.30, d (8.0)	10/10′	127.6	CH	7.20, m
9a	140.6	C		11	126.5	CH	7.20, m
10	39.6	CH_2_	4.50, d (12.0); 3.96, d (12.0)	12	64. 8	CH_2_	3.52, d (18.0); 3.00, d (18.0)
10a	70.8	C		13	12.8	CH_3_	2.11, s
11	28.9	CH_3_	3.20, s	14	13.5	CH_3_	2.29, s
12	63.9	CH_2_	3.62, d (16.8); 3.51, d (16.8)				
13	14.5	CH_3_	2.32, s				
14	13.8	CH_3_	2.24, s				

^a^ ^1^H and ^13^C NMR data were measured at 400/100 MHz in CDCl_3_; ^b^ ^1^H and ^13^C NMR data were measured at 300/75 MHz in DMSO-*d*_6_.

The relative stereochemistry of **14** was determined on the basis of NOESY data. The NOESY correlations of H-2 with H-12, H-13 and H-14 and of OH-3 with H-11 suggested that H-2 and H-11 were placed on the opposite face of the ring system and H-11 and the hydroxyl group at C-3 position were placed on the same face (Figure 3b). No NOESY correlation between H-2 and H-16 was observed (Supplementary Figure S68). Therefore, H-2 was assigned a β-orientation, whereas OH-3, H-11 and H-16 were determined to have an α-orientation.

The NMR data of compound **15** recorded in CDCl_3_ and acetone-*d*_6_ showed minor differences (Table 6 and Supplementary Figures S69–S75). By comparison of its spectral data with those reported in the literature, compound **15** was elucidated as pyripyropene A [31]. Pyripyropene A (**15**), previously isolated from *Aspergillus fumigatus* FO-1289, showed very potent inhibition of cholesterol acyltransferase (ACAT).

**Table 5:** ^1^H and ^13^C NMR data of compound **14** at 400/100 MHz, respectively, in CDCl_3_, δ in ppm.

Position	δ_C_	Type	δ_H_, mult., (*J* in Hz)
1		N	
2	84.8	CH	5.95, s
3	74.8	C	
4	135.2	C	
5	124.7	CH	7.43, brd (7.6)
6	126.4	CH	7.15, dd (7.6, 7.6)
7	130.9	CH	7.35, dd (7.6, 7.6)
8	115.6	CH	7.53, d (7.6)
9	138.5	C	
10	171.4	C	
11	70.3	CH	4.43, d (8.4)
12	30.1	CH	2.35, dqq (8.4, 6.4, 6.4)
13	20.1	CH_3_	1.17, d (6.4)
14	19.1	CH_3_	1.21, d (6.4)
15	36.5	CH_2_	2.49, dd (15.2, 5.2); 3.23, dd (15.2, 4.8)
16	56.8	CH	5.11, dd (5.2, 4.8)
17	163.6	C	
18		N	
19	161.7	C	
20	121.4	C	
21	127.4	CH	8.20, dd (7.6, 0.8)
22	127.8	CH	7.50, dd (7.6, 7.6)
23	134.9	CH	7.71, ddd (7.6, 7.6, 0.8)
24	126.1	CH	7.64, d (7.6)
25	144.4	C	
26		N	
27	147.5	CH	8.61, s
28		N	
3-OH			4.09, brs

**Table 6:** ^1^H and ^13^C NMR data of compound **15** at 600/150 MHz, respectively, δ in ppm.

Position	In CDCl_3_			In Acetone-*d*_6_	
	δ_C_	Type	δ_H_, mult., (*J* in Hz)	δ_C_	δ_H_, mult., (*J* in Hz)
1	73.5	CH	5.00, dd (10.8, 4.8)	74.5	4.99, dd (10.8, 4.8)
2	22.7	CH_2_	1.83, m; 1.90, m	23.7	1.85, m; 1.89, m
3	36.2	CH_2_	1.37, m; 2.16, m	36.8	1.49, m; 2.17, ddd (12.6, 4.8, 4.8)
4	37.9	C		38.8	
5	54.7	CH	1.53, d (4.2)	55.1	1.67, d (4.8)
6	83.3	C		83.9	
7	77.7	CH	4.78, dd (12.0, 4.8)	79.0	4.81, dd (11.4, 4.8)
8	25.2	CH_2_	1.63, ddd (12.0, 12.0, 11.4); 1.78, dd (11.4, 4.8)	26.0	1.74, m; 1.83, ddd (10.8, 5.4, 5.4)
9	45.4	CH	1.58, d (12.0)	46.2	1.72, d (12.0)
10	40.3	C		41.3	
11	64.8	CH_2_	3.77, d (11.4); 3.70, d (11.4)	65.6	3.76, d (12.0); 3.72, d (12.0)
12	17.4	CH_3_	1.43, s	17.9	1.53, s
13	60.1	CH	4.99, d (4.2)	60.5	5.00, d (4.8)
14	16.2	CH_3_	1.69, s	16.8	1.76, s
15	13.2	CH_3_	0.83, s	13.6	0.93, s
2′	163.6	C		163.3	
3′	103.3	C		104.2	
4′	162.0	C		162.7	
5′	99. 9	CH	6.48, s	100.1	6.71, s
6′	156.4	C		158.1	
2″	145.4	CH	9.05, s	147.7	9.08, s
3″	127.9	C		128.5	
4″	134.3	CH	8.20, d (8.4)	133.8	8.24, ddd (7.8, 1.8, 1.8)
5″	124.2	CH	7.51, brd ( 8.4)	124.7	7.53, dd (7.8, 4.8)
6″	149.8	CH	8.72, s	152.2	8.69, d (4.8)
1-O-CO-CH_3_	170.5	C		170.6	
7-O-CO-CH_3_	170.0	C		170.3	
11-O-CO-CH_3_	170.9	C		170.8	
1-O-CO-CH_3_	21.1	CH_3_	2.07, s	21.1	2.02, s
7-O-CO-CH_3_	21.2	CH_3_	2.15, s	21.2	2.10, s
11-O-CO-CH_3_	20.8	CH_3_	2.03, s	20.7	2.00, s
13-OH		OH	3.06, brs		2.90, brs

### 2.2. Proposed Biosynthetic Pathway

**Figure 4:**
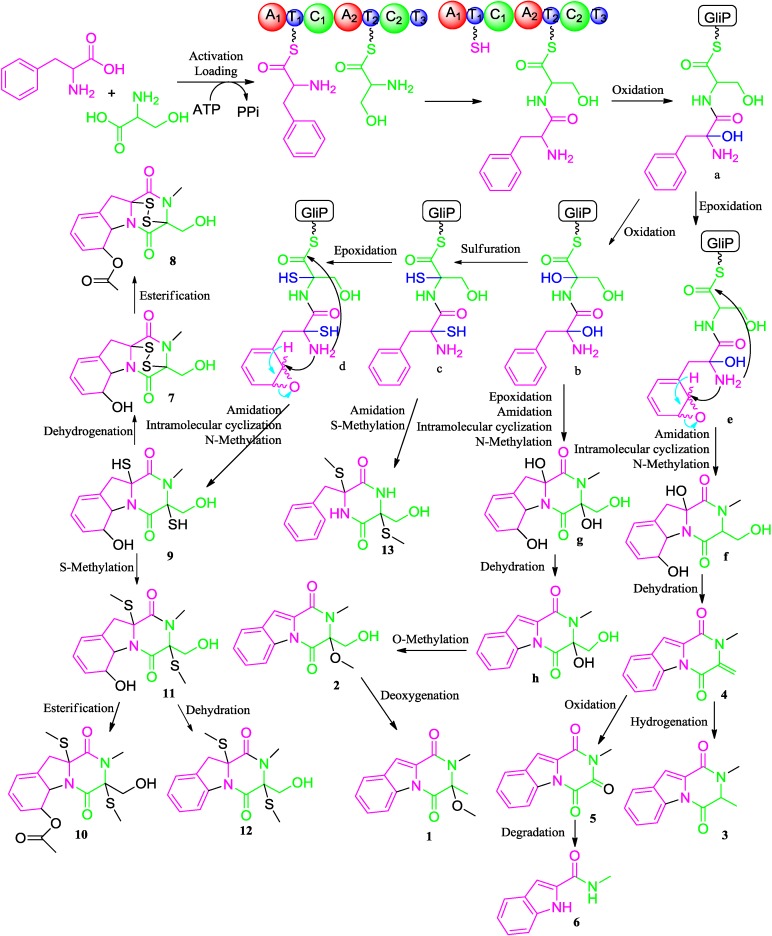
Proposed biosynthetic pathways of compounds **1**–**13**.

Diketopiperazines (DKPs) are typically synthesized via nonribosomal peptide synthetases (NRPS) to incorporate more than one amino acid from fungi [32]. Gliotoxin and its analogues have the diketopiperazine core with a disulfide bridge in an oxidized or reduced form. In the biosynthesis of gliotoxin, two-modular nonribosomal peptide synthetase, GliP, incorporates l-phe and l-ser to form dipeptidyl l-phe-l-ser, and under the action of the enzymes, the latter is converted to the corresponding diketopiperazines [33]. Therefore, the biosynthetic pathways of compounds **1**–**13** were proposed in Figure 4. Catalyzed by GliP, the intermolecular condensation between phenylalanine and serine generates an l-phe-l-ser, followed by successive oxidation, sulfuration and epoxidation reactions to obtain the intermediates, **a**–**d**. Then, **a** and **b** undergo epoxidation, amidation, intramolecular nucleophilic cyclization and *N*-methylation reactions to produce the intermediates, **f** and **g**, respectively. Dehydration of **f** furnishes compound **4**. Compound **4** is further hydrogenated and oxidized to afford **3** and **5**, respectively. The product, **g**, is sequentially dehydrated, *O*-methylated to form **2**, followed by the deoxygenation to produce **1**. After successive amidation and methylation of the thiol groups, the intermediate **c** generates compound **13**. Amidation, intramolecular nucleophilic cyclization and N-methylation of **d** afford compound **9**. Compound **9** is *S*-methylated to generate **11** or dehydrogenated to produce **7**, which further undergoes esterification to form **10** and **8**, separately. Compound **12** is produced by the dehydration of **11**, whereas compound **6** is the degradation product of **5**.

**Figure 5:**
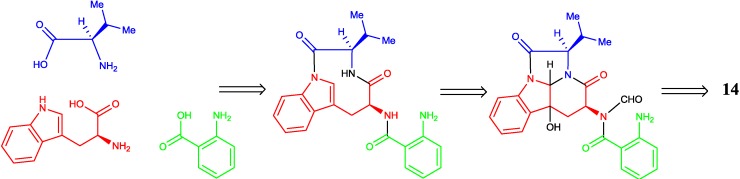
Proposed biosynthetic pathway of compound **14**.

Compound **14** possessed a unique tetracyclic-fused skeleton, and it was the diastereomer of pseudofischerine [34]. We postulated that the biosynthetic pathway may involve d-valine, d-tryptophan and anthranilic acid as the precursors (Figure 5). Chaetominine [35] and kapakahines [36] have a similar tetracyclic-fused fragment.

### 2.3. Biological Activity

Compounds **2**–**14** were evaluated for their antibacterial activity against three multidrug-resistant bacteria, *i.e.*, the Gram-positive *Staphylococcus aureus* (ATCC29213) and *Methicillin-resistant staphylococcus aureus* (R3708) and the Gram-negative *Escherichia coli* (ATCC25922), using a broth dilution method (Mueller–Hinton broth) [37]. Vancomycin and ampicillin sodium were used as positive controls. Compounds **7** and **9** displayed significant inhibitory activities against these three bacteria with MIC values ranging from 1.52 to 97.56 μM (Table 7). Compounds **4** and **8** inhibited the growth of *Staphylococcus aureus* ATCC29213 and R3708 with MIC values of 283.11, 70.70 μM and 86.91, 21.73 μM, respectively. The remaining nine compounds, **2**, **3**, **5**, **6** and **10**–**14**, were inactive in this assay (MIC > 256 μg/mL). The results suggested that the bioactive compounds are more active against the Gram-positive bacteria. Especially, compounds **7** and **9** showed potent inhibition against *Staphylococcus aureus* R3708 with MIC values of 1.53 and 1.52 μM. Preliminary analysis of the structure-activity relationships of these twelve diketopiperazines suggests that the disulfide bridge or reduced disulfide bond is essential for the inhibitory activity. If the thiol groups are substituted, like compounds **10**–**13**, the inhibitory effects disappeared. The substitution at the six-membered ring containing two conjugated double bonds influences the intensity of antibacterial activity. The analogues with a hydroxyl group at C-6 enhance the antibacterial activity compared to the analogues with an acetyl group at the same position. Additionally, the α-methylene ketone group is also the pharmacophore for the antibacterial activity.

**Table 7:** Antibacterial activities of diketopiperazines **4** and **7**–**9** (MIC, μM, *n* = 3).

Compound	*Staphylococcus aureus* (ATCC29213)	*Staphylococcus aureus* (R3708)	*Escherichia coli* (ATCC25922)
**4**	283.11	70.70	>1,132
**7**	12.20	1.53	24.53
**8**	86.91	21.73	>695.65
**9**	48.78	1.52	97.56
Vancomycin	0.84	2.01	
Ampicillin sodium	8.07	129.24	6.73

Furthermore, compounds **4**, **5** and **7**–**13** were screened for their cytotoxic activities on the human embryonic kidney (HEK) 293 cell line and human colon cancer cell lines, HCT-116 and RKO (a poorly differentiated colon carcinoma cell line). Compounds **4**, **7**–**9** and **11** exhibited potent cytotoxicities against these cell lines (Table 8). With a disulfide bridge in the molecule, compounds **7** and **8** showed potent cytotoxic activities. Compound **4** showed stronger inhibitory activities than compound **5**; their structural difference is a typical α-methylene ketone group in **4**, whereas a diketone in **5**. Compared to compounds **9** and **11**, compound **10** lacked any activity (IC_50_ > 50 μM), supposedly since the thiol groups at C-3 and C-10a were methylated and the 6-OH was acetylated. The cytotoxic activities of the other compounds, due to the limited sample amount, were not tested in this assay.

**Table 8:** Cytotoxicities of compounds **4**, **5** and **7**–**13** (IC_50_, μM, *n* = 5).

Compound		Cell line	
	293	HCT-116	RKO
4	30.10 ± 0.90	10.34 ± 1.41	33.56 ± 1.22
5	>50	>50	>50
7	1.58 ± 0.03	1.24 ± 0.38	0.80 ± 0.20
8	4.49 ± 0.24	0.89 ± 0.04	1.24 ± 0.18
9	1.26 ± 0.04	0.43 ± 0.04	0.41 ± 0.07
10	>50	>50	>50
11	16.39 ± 0.38	8.59 ± 0.96	10.32 ± 0.04
12	>50	>50	>50
13	>50	>50	>50
5-Fluorouracil		2.04 ± 0.22	45.86 ± 4.58

## 3. Experimental Section

### 3.1. General Experimental Procedures

Preparative HPLC was performed using a Shimadzu LC-20AT HPLC pump (Shimadzu Corporation, Nakagyo-ku, Kyoto, Japan) equipped with an SPD-20A dual λ absorbance detector (Shimadzu Corporation, Nakagyo-ku, Kyoto, Japan) and a Shim-pack PRC-ODS HPLC column (250 × 20 mm, Shimadzu Corporation, Nakagyo-ku, Kyoto, Japan). Optical rotations were measured using a Schmidt and Haensch Polartronic HNQW5 optical rotation spectrometer (SCHMIDT + HAENSCH GmbH & Co., Berlin, Germany). CD spectra were measured on a JASCO J-810 circular dichroism spectrometer (JASCO International Co. Ltd., Hachioji, Tokyo, Japan). UV spectra were recorded on a Shimadzu UV-Vis-NIR spectrophotometer (Shimadzu Corporation, Nakagyo-ku, Kyoto, Japan). 1D and 2D NMR spectra were recorded on Bruker Avance IIIT 600HD and Bruker Avance II 400 spectrometers (Bruker BioSpin AG, Industriestrasse 26, Fällanden, Switzerland) and a Varian Mercury-Plus 300 spectrometer (Varian Medical Systems In., Salt Lake City, UT, USA). The chemical shifts are relative to the residual solvent signals (CDCl_3_: δ_H_ 7.26 and δ_C_ 77.0; DMSO-*d*_6_: δ_H_ 2.50 and δ_C_ 39.51; acetone-*d*_6_: δ_H_ 2.05 and δ_C_ 29.92). Mass spectra were obtained on Thermo DSQ EI low-resolution and Thermo MAT95XP EI high-resolution mass spectrometers (Thermo Fisher Scientific In., Waltham, MA, USA).

### 3.2. Fungal Strain and Culture Method

The marine fungus, *Neosartorya pseudofischeri* (Collection No. 2014F27-1), was isolated from the inner tissue of the sea star, *Acanthaster planci*, collected from Hainan Sanya National Coral Reef Reserve, China. This fungal strain was maintained in 15% (v/v) glycerol aqueous solution at −80 °C. A voucher specimen was deposited in the School of Pharmaceutical Sciences, Sun Yat-sen University, Guangzhou, China. Analysis of the ITS rDNA (GenBank KF999816) by BLAST database screening provided a 100% match to *N. pseudofischeri*.

This fungus were cultured in GlyPY medium (glycerol 10 g, peptone 5 g, yeast extract 2 g, CaCO_3_ 1 g, sea water 1 L) and GluPY medium (glucose 10 g, peptone 5 g, yeast extract 2 g, sea water 1 L, pH 7.5), respectively. Fungal mycelia were cut and transferred aseptically to 500 mL Erlenmeyer flasks each containing 200 mL sterilized GlyPY or GluPY liquid media. The flasks were incubated at 28 °C on a rotary shaker (120 rpm) for 25 days.

### 3.3. Extraction and Isolation

Fifty liters of GlyPY growth culture broth were filtered through cheesecloth. The culture broth was successively extracted with EtOAc (50 L) three times. The EtOAc extract was concentrated by low-temperature rotary evaporation. The extract (9.8 g) was chromatographed on a silica gel column (diameter: 8 cm, length: 70 cm, silica gel, 200 g) using petroleum ether (2 L), EtOAc (2 liters) (100:0–0:100, v/v), followed by EtOAc (2 L) and MeOH (2 L) (100:0–0:100, v/v) as the eluent to afford 10 fractions (code Fraction 1–Fraction 10). Fraction 2 was purified by the recrystallization in the petroleum ether-EtOAc (3:1, v/v) solution to give compounds **4** (13.4 mg) and **5** (52.7 mg). Fraction 3 was separated with a preparative RP HPLC using a gradient elution MeOH-H_2_O (20:80 up to 100:0, v/v) and then on Sephadex LH-20 using MeOH as the eluent followed by preparative RP HPLC eluted with MeOH–H_2_O (70:30, v/v) to give compounds **1** (1.3 mg), **2** (2.2 mg), **3** (1.62 mg) and **12** (2.15 mg). Fraction 5 was purified with a preparative RP HPLC (MeOH–H_2_O, 65:35, v/v) to give compound **6** (2.3 mg). Compound **10** (7.2 mg) was obtained from Fraction 7 with a preparative RP HPLC (MeOH–H_2_O, 70:30, v/v).

Fifty liters of GluPY growth culture broth were filtered through cheesecloth. The culture broth was successively extracted with EtOAc (50 L) three times to afford 10.2 g of extract. The crude extract was separated as ten fractions (Fraction 1–10) by a silica gel column chromatograph (diameter: 8 cm; length: 70 cm; silica gel, 200 g) employing the gradient elution described above. Fraction 3 displayed interesting signals in the δ_H_ 8~9 region and was further purified by RP HPLC using MeCN–H_2_O (40:60, v/v) as the eluent to yield compounds **14** (3.2 mg) and **15** (4.0 mg). Fraction 4 was separated on a silica gel column chromatograph (diameter: 3 cm; length: 50 cm; silica gel, 50 g) using an elution with petroleum ether (300 mL) and EtOAc (300 mL) (50:50, v/v) to obtain five fractions (Fractions 4–1 to Fractions 4–5). Fractions 4–2 was further purified on preparative RP HPLC eluted with MeOH–H_2_O (75:25, v/v) to give compound **7** (32 mg), under the same experiment condition. Fraction 4–4 afforded compounds **10** (10.2 mg), **11** (71.3 mg) and **13** (23.1mg). Fraction 7 was purified on RP HPLC (MeOH–H_2_O, 60:40, v/v) to give compounds **8** (7.5 mg) and **9** (9.3 mg).

Neosartin A (**1**): Yellowish solid. UV (MeOH) λ_max_ (ε) 211 (25,800), 243 (30,637), 295 (21,930) nm. IR: υ_max_ 3000, 2926, 1716, 1656, 1589, 1576, 1427, 1391, 1357, 1228, 1112, 1043, 844, 750 cm^−1^. ^1^H and ^13^C NMR: Table 1. LREIMS: *m/z* 256, 243, 227, 215, 199, 187, 170, 156, 143, 129, 115, 103, 92, 89, 78, 72, 63, 56. HREIMS: *m/z* [M]^+^ calcd. for C_14_H_14_O_3_N_2_: 258.0999; found 258.0998.

Neosartin B (**2**): Yellowish solid. UV (MeOH) λ_max_ (ε) 212 (17,237), 243 (21,941), 295 (16,195) nm. IR: υ_max_ 3301, 3126, 2951, 1706, 1635, 1591, 1577, 1435, 1392, 1360, 1348, 1224, 1151, 1116, 1081, 981, 746, 733 cm^−1^. ^1^H and ^13^C NMR: Table 1. LREIMS: *m/z* 274, 260, 243, 229, 215, 202, 188, 172, 156, 143, 130, 115, 103, 89, 83, 72, 57. HREIMS: *m/z* [M]^+^ calcd. for C_14_H_14_O_4_N_2_: 274.0948; found 274.0947.

1,2,3,4-Tetrahydro-2,3-dimethyl-1,4-dioxopyrazino[1,2-a]indole (**3**): White solid. ^1^H and ^13^C NMR: Table 1. LREIMS: *m/z* 228, 213, 200, 185, 172, 143, 131, 115, 100, 89, 71, 62, 56.

1,2,3,4-Tetrahydro-2-methyl-3-methylene-1,4-dioxopyrazino[1,2-a]indole (**4**): White solid. ^1^H and ^13^C NMR: Table 2. LREIMS: *m/z* 226, 199, 185, 169, 157, 143, 129, 115, 99, 88, 75, 62, 55.

1,2,3,4-Tetrahydro-2-methyl-1,3,4-trioxopyrazino[1,2-a]indole (**5**): White solid. ^1^H and ^13^C NMR: Table 2. LREIMS: *m/z* 228, 200, 159, 143, 131, 115, 100, 88, 71, 62, 50.

*N*-methyl-1*H*-indole-2-carboxamide (**6**): White solid. ^1^H and ^13^C NMR: Table 2. LREIMS: *m/z* 174, 156, 143, 115, 89, 77, 63, 58.

Gliotoxin (**7**): Pale yellowish solid. ^1^H NMR (CDCl_3_, 400 MHz): 5.98 (1H, d, 4.0, H-7), 5.92 (1H, dd, 9.2, 4.2, H-8), 5.84 (1H, s, OH-13), 5.75 (1H, d, 9.2, H-9), 4.80 (2H, s, H-5a, H-6), 4.43 (1H, d, 17.6, H-12), 4.23 (1H, d, 17.6, H-12), 4.06 (1H, brs, OH-12), 3.73 (1H, d, 12.8, H-10), 3.19 (3H, s, CH_3_-11), 2.94 (1H, d, 12,8, H-10); ^13^C (CDCl_3_, 100 MHz): 166.0 (C-1), 165.1 (C-4), 130.8 (C-9a), 129.8 (C-7), 123.3 (C-8), 120.1 (C-9), 77.3 (C-3), 75.8 (C-10a), 73.1 (C-6), 69.7 (C-5a), 60.4 (C-12), 36.5 (C-10), 27.5 (C-11). LREIMS: *m/z* 326, 308, 277, 262, 244, 233, 217, 199, 188, 160, 144, 132, 107, 89, 77, 73, 64, 55, 42.

Acetylgliotoxin (**8**): Yellowish solid. ^1^H and ^13^C NMR: Table 3.

Reduced gliotoxin (**9**): Yellowish solid. ^1^H and ^13^C NMR: Table 3.

6-Acetylbis(methylthio)gliotoxin (**10**): Yellowish oil. ^1^H NMR (CDCl_3_, 400 MHz): 6.16 (1H, d, 14.8, H-7), 5.90 (2H, m, H-8, H-9), 5.75 (1H, d, 10.4, H-6), 5.01 (1H, d, 14.4, H-5a), 4.23 (1H, d, 11.2, H-12), 3.76 (1H, d, 11.6, H-12), 3.60 (1H, brs, OH-12), 3.09 (3H, s, CH_3_), 3.04 (1H, d, 16.0, H-10), 2.90 (1H, d, 16.0, H-10), 2.29 (3H, s, CH_3_-13), 2.12 (3H, s, CH_3_-15), 2.05 (3H, s, CO*CH_3_*-14); ^13^C (CDCl_3_, 100 MHz): 170.7 (*CO*CH_3_-14), 166.4 (C-1), 164.4 (C-4), 133.8 (C-9a), 127.8 (C-7), 123.3 (C-8), 119.8 (C-9), 72.7 (C-3), 72.3 (C-10a), 75.2 (C-6), 65.4 (C-5a), 63.6 (C-12), 40.1 (C-10), 28.7 (C-11), 21.3 (CO*CH_3_*-14), 15.0 (C-13), 13.0 (CH_3_-15).

Bisdethiobis(methylthio)gliotoxin (**11**): Yellowish oil. ^1^H NMR (CDCl_3_, 400 MHz): 5.85 (2H, m, H-7, H-8), 5.65 (1H, d, 9.2, H-9), 4.86 (2H, s, H-5a, H-6), 4.30 (1H, d, 11.6, H-12), 3.82 (1H, d, 11.6, H-12), 3.09 (3H, s, CH_3_-11), 3.01 (1H, d, 16.0, H-10), 2.92 (1H, d, 16.0, H-10), 2.22 (3H, s, CH_3_-13), 2.19 (3H, s, CH_3_-15); ^13^C (CDCl_3_, 100 MHz): 166.7 (C-1), 165.9 (C-4), 131.7 (C-9a), 129.5 (C-7), 123.2 (C-8), 119.8 (C-9), 74.2 (C-3), 72.1 (C-10a), 71.4 (C-6), 69.4 (C-5a), 63.4 (C-12), 38.7 (C-10), 28.5 (CH_3_-11), 15.0 (CH_3_-13), 13.0 (CH_3_-15) .

Didehydrobisdethiobis(methylthio)gliotoxin (**12**): Yellowish oil. ^1^H and ^13^C NMR: Table 4.

Bis-*N*-norgliovictin (**13**): White solid. ^1^H and ^13^C NMR: Table 4.

Neosartin C (**14**): Yellowish solid. ${\left[\text{α}\right]}_{D}^{20}$: −58.17°(*c* =0.0822, MeOH). UV (MeOH) λ_max_ (ε) 314 (2910), 301 (3653), 226 (35,975), 212 (29,412) nm. IR: υ_max_ 3348, 3068, 2970, 2934, 2875, 1724, 1667, 1608, 1476, 1465, 1387, 1326, 1292, 1267, 1248, 1172, 1112, 1076, 1007, 976, 915, 891, 844, 764, 742, 700, 593, 560 cm^−1^. ^1^H and ^13^C NMR: Table 5. LREIMS: *m/z* 430, 412, 341, 329, 314, 298, 286, 266, 238, 224, 184, 147, 130, 102, 83, 76, 55. HREIMS: *m/z* [M]^+^ calcd. for C_24_H_22_O_4_N_4_: 430.1636; found 430.1635.

Pyripyropene A (**15**): Yellowish solid. UV (MeOH) λ_max_ (ε) 320 (18,029), 231 (29,463), 209 (15,006) nm. IR: υ_max_ 3409, 2975, 2947, 2883, 1723, 1644, 1580, 1481, 1436, 1394, 1370, 1234, 1159, 1109, 1090, 1074, 1040, 1026, 1009, 984, 960, 925, 873, 809, 763, 704, 687, 650, 603, 583 cm^−1^. ^1^H and ^13^C NMR: Table 6. LREIMS: *m/z* 583, 565, 550, 523, 505, 494, 463, 445, 430, 403, 385, 358, 334, 304, 283, 240, 218, 202, 190, 171, 157, 148, 133, 119, 106, 95, 79, 69, 55.

### 3.4. Antibacterial Activity Assay

The MIC values were determined using a broth dilution method (Mueller–Hinton broth) based on the National Committee for Clinical Laboratory Standards (NCCLS) standard. The starting concentrations of the tested compounds were 256 µg/mL (from 256 to 0.25). The solution of compound in DMSO (10 µL) was added to 90 µL of bacterial culture (1 × 10^6^ CFU/mL) in the first well of flat-bottomed 96-well tissue culture plates. The solution was then double diluted. The bacterial culture solution containing the appropriate compound (50 µL) was discarded from the last well in order to ensure a 100-µL volume of bacterial culture in every well. A set of tubes containing only inoculated broth and solvent were kept as controls. The plate was incubated at 37 °C overnight in an electro-heating standing-temperature cultivator before the measurement of the absorbance value. The optical density values at 600 nm were measured using a multifunction microplate reader (PowerWaveTM XS2, BioTek^®^ Instruments Inc., Winooski, VT, USA). Vancomycin and ampicillin sodium were used as positive controls.

### 3.5. Cytotoxicity Assay

Compounds in DMSO at 50 mM were used in the MTS ((3-(4,5-dimethylthiazol-2-yl)-5-(3-carboxymethoxyphenyl)-2-(4-sulfophenyl)-2*H*-tetrazolium)) assay. 5-Fluorouracil was used as the positive control, and DMSO was used as the negative control. 293, HCT116 and RKO cells were suspended in fresh RPMI-1640 medium containing 10% fetal bovine serum and 100 μg/mL penicillin and streptomycin at a cell density of 1 × 10^5^ cells/mL and seeded into 96-well plates each 100 μL/well. The suspension cells, 293, HCT116 and RKO cells, were incubated at 37 °C for 12 h. Then, compounds were added to the cultures at different concentrations; then, the cells were cultured at 37 °C for 72 h. Twenty microliters of MTS/PMS (phenazine methosulfate) were added into each well, incubated at 37 °C for 4 h in a humidified, 5% CO_2_ atmosphere. The absorbance at 490 nm was recorded using a Thermo Scientific Varioskan Flash Multimode Reader (Thermo Fisher Scientific Inc., Waltham, MA, USA). The data were analyzed with the GraphPad Prism 6 software package [38].

## 4. Conclusions

Alternating the cultivation parameters systematically, such as the components of the media, to enhance the diversity of secondary metabolites produced by the fungus is a well-known, simple and efficient technique. Using this strategy, we isolated and identified fifteen compounds, including twelve diketopiperazine derivatives, a meroterpenoid, an alkaloid with a unique tetracyclic-fused skeleton and an imidazole analogue from the two cultures. The discovery of these compounds provided further evidence that the genus of *Neosartorya* is a rich source of nitrogen-containing natural products. Interestingly, the dominant metabolites from the GluPY medium were the diketopiperazines with disulfide bonds; however, the main compounds from the GlyPY medium were the diketopiperazines without a disulfide bond. The biosynthetic pathways of the unique alkaloids are complex and diverse. Hopefully, further investigation on the secondary metabolites of *Neosartorya pseudofischeri* in varied culture conditions supplied with amino acid precursors may find more novel alkaloids and improve their production. Most of the metabolites showed significant antibacterial and cytotoxic activities. Based on the structure-activity relationship analysis, the disulfide bridge, the α-methylene ketone group, the hydroxyl group at C-6 and the thiol groups were considered as the pharmacophores.

## Supplementary Files

Supplementary File 1
