# Supplementary material for: Exploring the Chemodiversity and Biological Activities of the Secondary Metabolites from the Marine Fungus Neosartorya pseudofischeri
Source: Mar Drugs. 2014 Nov 24;12(11):5657–76. doi: 10.3390/md12115657 (PMC4245550; doi:10.3390/md12115657)
Supplement: Supplementary File 1 [file marinedrugs-12-05657-s001.pdf]

Supplementary Information

Figure S1. HPLC analysis on the fungal metabolites in different culture media.
Figure S2. LREIMS of neosartin A (**1**).
Figure S3. HREIMS of neosartin A (**1**).
Figure S4. ^{1}H NMR (400 MHz, $CDCl_3$) spectrum of neosartin A (**1**).
Figure S5. ^{13}C NMR (100 MHz, $CDCl_3$) spectrum of neosartin A (**1**).
Figure S6. gHMQC of neosartin A (**1**).
Figure S7. ^{1}H-^{1}H gCOSY of neosartin A (**1**).
Figure S8. gHMBC of neosartin A (**1**).
Figure S9. NOESY of neosartin A (**1**).
Figure S10. LREIMS of neosartin B (**2**).
Figure S11. HREIMS of neosartin B (**2**).
Figure S12. ^{1}H NMR (400 MHz, $CDCl_3$) spectrum of neosartin B (**2**).
Figure S13. ^{13}C NMR (100 MHz, $CDCl_3$) spectrum of neosartin B (**2**).
Figure S14. gHMQC spectrum of neosartin B (**2**).
Figure S15. ^{1}H-^{1}H gCOSY spectrum of neosartin B (**2**).
Figure S16. gHMBC spectrum of neosartin B (**2**).
Figure S17. NOESY of neosartin B (**2**).
Figure S18. LREIMS of 1,2,3,4-tetrahydro-2,3-dimethyl-1,4- dioxopyrazino[1,2-a]indole (**3**).
Figure S19. ^{1}H NMR (400 MHz, $CDCl_3$) spectrum of 1,2,3,4-tetrahydro- 2,3-dimethyl-1,4-dioxopyrazino[1,2-a]indole (**3**).
Figure S20. ^{13}C NMR (100 MHz, $CDCl_3$) spectrum of 1,2,3,4-tetrahydro-2,3-dimethyl- 1,4-dioxopyrazino[1,2-a]indole (**3**).
Figure S21. gHMQC spectrum of 1,2,3,4-tetrahydro-2,3-dimethyl-1,4- dioxopyrazino[1,2-a]indole (**3**).
Figure S22. ^{1}H-^{1}H gCOSY spectrum of 1,2,3,4-tetrahydro-2,3-dimethyl-1,4- dioxopyrazino[1,2-a]indole (**3**).
Figure S23. gHMBC spectrum of 1,2,3,4-tetrahydro-2,3-dimethyl-1,4- dioxopyrazino[1,2-a]indole (**3**).
Figure S24. NOESY of 1,2,3,4-tetrahydro-2,3-dimethyl-1,4- dioxopyrazino[1,2-a]indole (**3**).
Figure S25. LREIMS of 1,2,3,4-tetrahydro-2-methyl-3-methylene-1,4- dioxopyrazino[1,2-a]indole (**4**).
Figure S26. ^{1}H NMR (400 MHz, $CDCl_3$) spectrum of 1,2,3,4-tetrahydro-2-methyl-3-methylene- 1,4-dioxopyrazino[1,2-a]indole (**4**).
Figure S27. ^{13}C NMR (100 MHz, $CDCl_3$)spectrum of 1,2,3,4-tetrahydro-2-methyl-3- methylene- 1,4-dioxopyrazino[1,2-a]indole (**4**).

Figure S28. gHMQC spectrum of 1,2,3,4-tetrahydro-2-methyl-3-methylene- 1,4-dioxopyrazino [1,2-a]indole (**4**).
Figure S29. ^{1}H-^{1}H gCOSY spectrum of 1,2,3,4-tetrahydro-2-methyl-3-methylene- 1,4-dioxopyrazino[1,2-a]indole (**4**).
Figure S30. gHMBC spectrum of 1,2,3,4-tetrahydro-2-methyl-3- methylene-1,4-dioxopyrazino [1,2-a]indole (**4**).
Figure S31. NOESY of 1,2,3,4-tetrahydro-2-methyl-3-methylene-1,4-dioxopyrazino[1,2-a]indole (**4**).
Figure S32. LREIMS of 1,2,3,4-tetrahydro-2-methyl-1,3,4- trioxopyrazino[1,2-a]indole (**5**).
Figure S33. ^{1}H NMR (400 MHz, DMSO-*d*6) spectrum of 1,2,3,4-tetrahydro-2-methyl-1,3,4- trioxopyrazino[1,2-a]indole (**5**).
Figure S34. ^{13}C NMR (100 MHz, DMSO-*d*6) spectrum of 1,2,3,4-tetrahydro-2-methyl-1,3,4- trioxopyrazino[1,2-a]indole(**5**).
Figure S35. gHMQC spectrum of 1,2,3,4-tetrahydro-2-methyl-1,3,4- trioxopyrazino[1,2-a]indole (**5**).
Figure S36. ^{1}H-^{1}H gCOSY spectrum of 1,2,3,4-tetrahydro-2-methyl-1,3,4- trioxopyrazino[1,2-a]indole (**5**).
Figure S37. gHMBC spectrum of 1,2,3,4-tetrahydro-2-methyl-1,3,4- trioxopyrazino[1,2-a]indole (**5**).
Figure S38. NOESY of 1,2,3,4-tetrahydro-2-methyl-1,3,4-trioxopyrazino[1,2-a]indole (**5**).
Figure S39. LREIMS of *N*-methyl-1*H*-indole-2-carboxamide (**6**).
Figure S40. ^{1}H NMR (400 MHz, acetone-*d*6) spectrum of *N*-methyl-1*H*-indole- 2-carboxamide (**6**).
Figure S41. ^{13}C NMR (100 MHz, acetone-*d*6) spectrum of *N*-methyl-1*H*-indole-2- carboxamide (**6**).
Figure S42. gHMQC spectrum of *N*-methyl-1*H*-indole-2-carboxamide (**6**).
Figure S43. ^{1}H-^{1}H gCOSY spectrum of *N*-methyl-1*H*-indole-2-carboxamide (**6**).
Figure S44. gHMBC spectrum of *N*-methyl-1*H*-indole-2-carboxamide (**6**).
Figure S45. NOESY of *N*-methyl-1*H*-indole-2-carboxamide (**6**).
Figure S46. LREIMS of gliotoxin (**7**).
Figure S47. ^{1}H NMR (300 MHz, $CDCl_3$) spectrum of gliotoxin (**7**).
Figure S48. ^{13}C NMR (75 MHz, $CDCl_3$) spectrum of gliotoxin (**7**).
Figure S49. ^{1}H NMR (300 MHz, $CDCl_3$) spectrum of acetylgliotoxin (8).
Figure S50. ^{13}C NMR (75 MHz, $CDCl_3$) spectrum of acetylgliotoxin (8).
Figure S51. ^{1}H NMR (400 MHz, $CDCl_3$) spectrum of reduced gliotoxin (**9**).
Figure S52. ^{13}C NMR (100 MHz, CDCl3) spectrum of reduced gliotoxin (**9**).
Figure S53. ^{1}H NMR (400 MHz, $CDCl_3$) spectrum of 6-acetylbis(methylthio)gliotoxin (**10**).
Figure S54. ^{13}C NMR (100 MHz, $CDCl_3$) spectrum of 6-acetylbis(methylthio)gliotoxin (**10**).

Figure S55. ^{1}H NMR (300 MHz, $CDCl_3$) spectrum of bisdethiobis(methylthio)gliotoxin (**11**).
Figure S56. ^{13}C NMR (100 MHz, $CDCl_3$) spectrum of bisdethiobis(methylthio)gliotoxin (**11**).
Figure S57. ^{1}H NMR (400 MHz, $CDCl_3$) spectrum of didehydrobisdethiobis(methy1thio)gliotoxin (**12**).
Figure S58. ^{13}C NMR (100 MHz, $CDCl_3$) spectrum of didehydrobisdethiobis(methy1thio)gliotoxin (**12**).
Figure S59. ^{1}H NMR (300 MHz, DMSO-*d*6) spectrum of bis-N-norgliovictin (**13**).
Figure S60. ^{13}C NMR (75 MHz, DMSO-*d*6) spectrum of bis-N-norgliovictin (**13**)
Figure S61. LREIMS of neosartin C (**14**).
Figure S62. HREIMS of neosartin C (**14**).
Figure S63. ^{1}H NMR (400 MHz, $CDCl_3$) spectrum of neosartin C (**14**).
Figure S64. ^{13}C NMR (100 MHz, $CDCl_3$) spectrum of neosartin C (**14**).
Figure S65. gHMQC spectrum of neosartin C (**14**).
Figure S66. ^{1}H-^{1}H gCOSY spectrum of neosartin C (**14**).
Figure S67. gHMBC spectrum of neosartin C (**14**).
Figure S68. NOESY of neosartin C (**14**).
Figure S69. LREIMS of pyripyropene A (**15**).
Figure S70. ^{1}H NMR (400 MHz, $CDCl_3$) spectrum of pyripyropene A (**15**).
Figure S71. ^{13}C NMR (100 MHz, $CDCl_3$) spectrum of pyripyropene A (**15**).
Figure S72. gHMQC of pyripyropene A (**15**).
Figure S73. ^{1}H-^{1}H gCOSY of pyripyropene A (**15**).
Figure S74. gHMBC of pyripyropene A (**15**).
Figure S75. NOESY of pyripyropene A (**15**).

Figure S1. HPLC analysis on the fungal metabolites in different culture media.

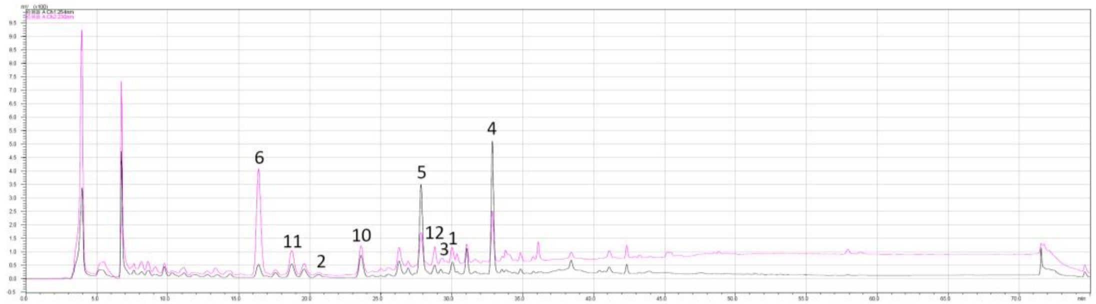

(A) Medium: glycerol 10 g, peptone 5 g, yeast extract 2 g, $CaCO_3$ 1 g, seawater 1 L.

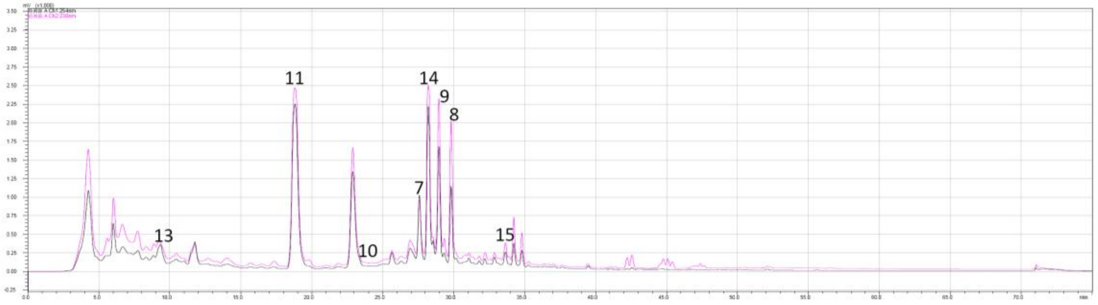

(B) Medium: glucose 10 g, peptone 5 g, yeast extract 2 g, pH 7.5, seawater 1 L.

Shimadzu LC-20AT HPLC pump (Shimadzu Corporation, Nakagyo-ku, Kyoto, Japan) equipped with an SPD-20A dual λ absorbance detector (Shimadzu Corporation, Nakagyo-ku, Kyoto, Japan) and a Shim-pack PRC-ODS HPLC column (250×4.6 mm, 5 μm). LC Time Program.

Time/min	Module	Action	Value/%
0.01	Pumps	B.Conc	30
10.00	Pumps	B.Conc	30
40.00	Pumps	B.Conc	100
60.00	Pumps	B.Conc	100
65.00	Pumps	B.Conc	30
70.00	Pumps	B.Conc	30
75.00	Controller	Stop	

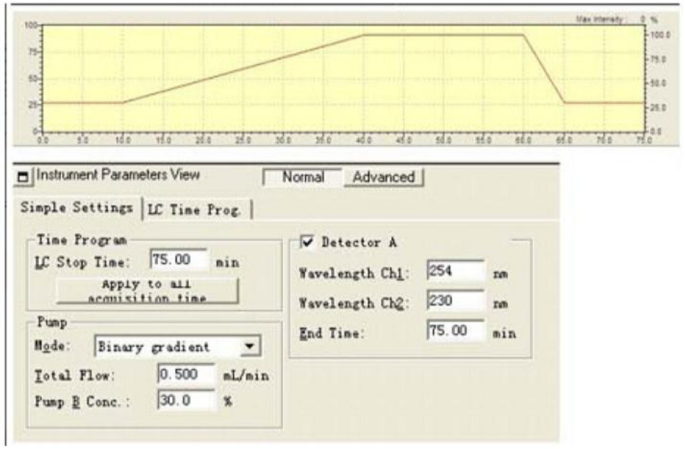

Figure S2. LREIMS of neosartin A (**1**).

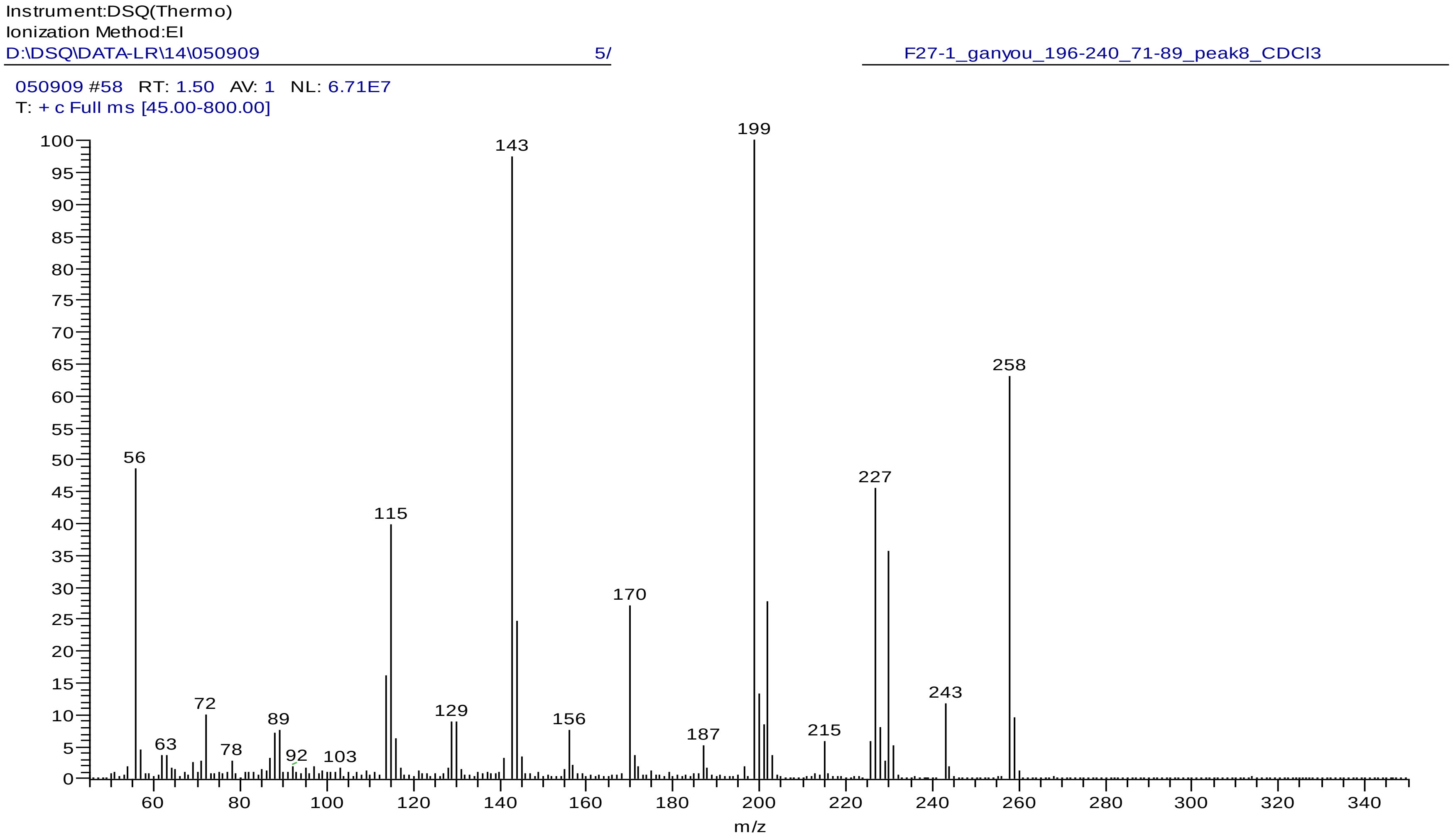

Figure S3. HREIMS of neosartin A (**1**).

SPECTRUM - MS
File : D:\DATA-HR\14\051303-f27-c3.RAW
Full ms [252.500 - 271.500] - Range: 258.000 - 258.300
Scan No. 13 of 20
Scan #: 13
RT: 0.50
Data points: 1

Mass	Relative Intensity	Theoretical Mass	Delta [ppm]	Delta [mmu]	RDB	Composition
258.0998	90.6	258.0999	-0.2	-0.1	9.0	$C_{14}H_{14}O_3N_2$

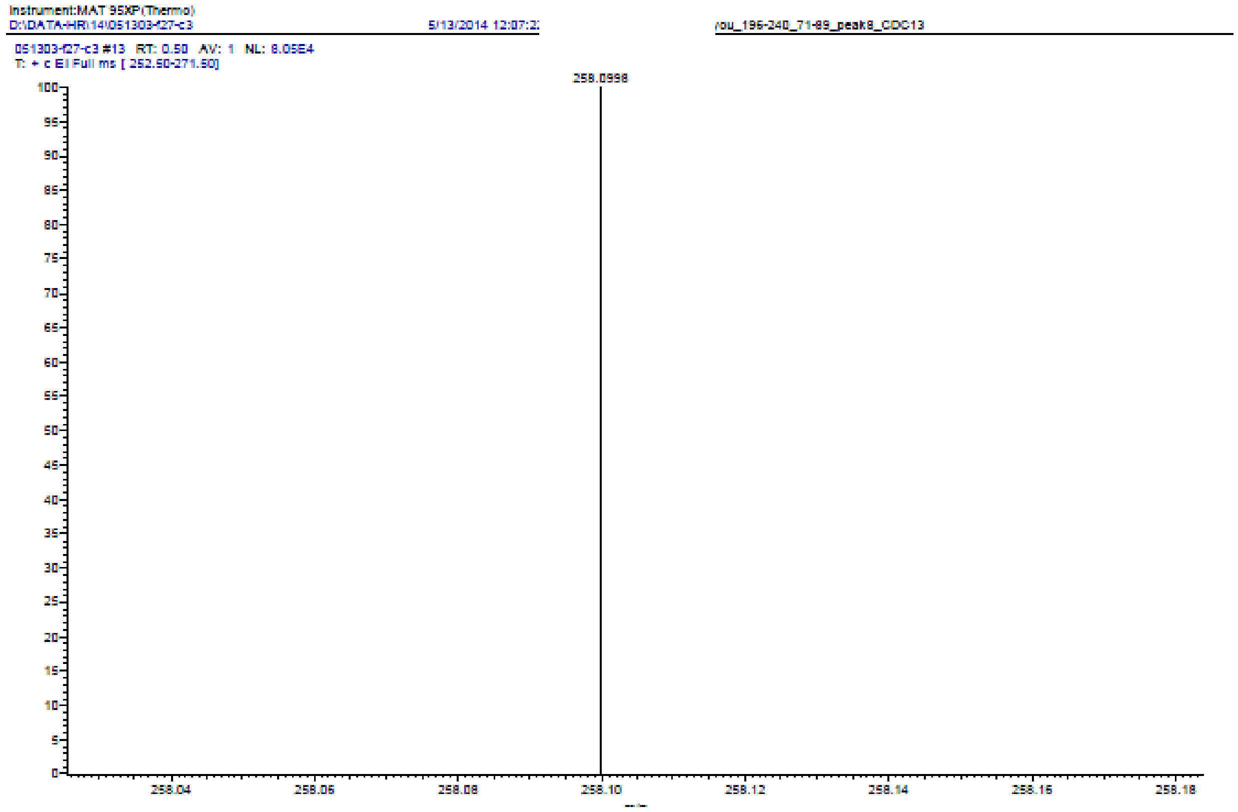

Figure S4. ^{1}H NMR (400 MHz, $CDCl_3$) spectrum of neosartin A (**1**).

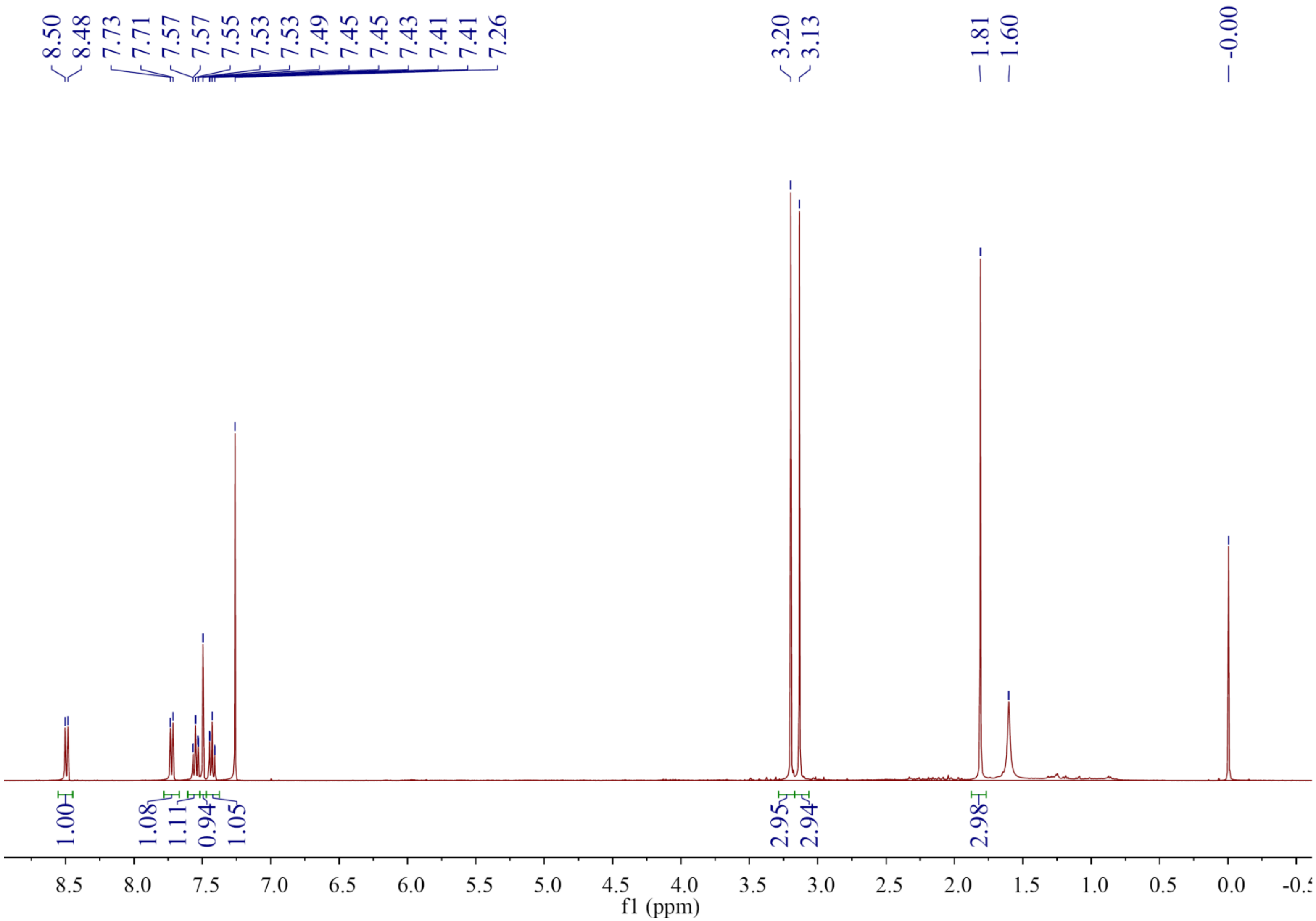

Figure S5. ^{13}C NMR (100 MHz, $CDCl_3$) spectrum of neosartin A (**1**).

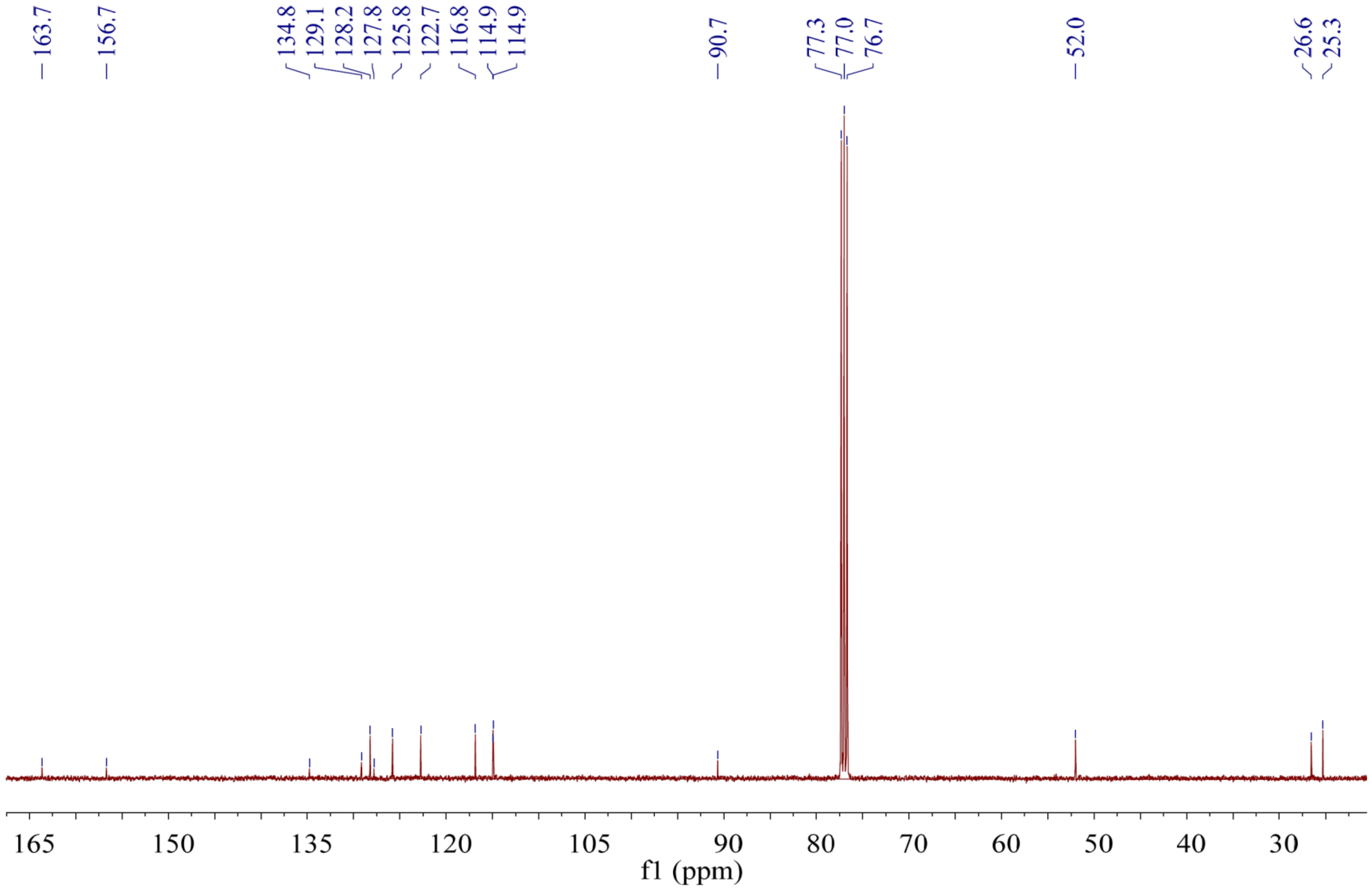

Figure S6. gHMQC of neosartin A (**1**).

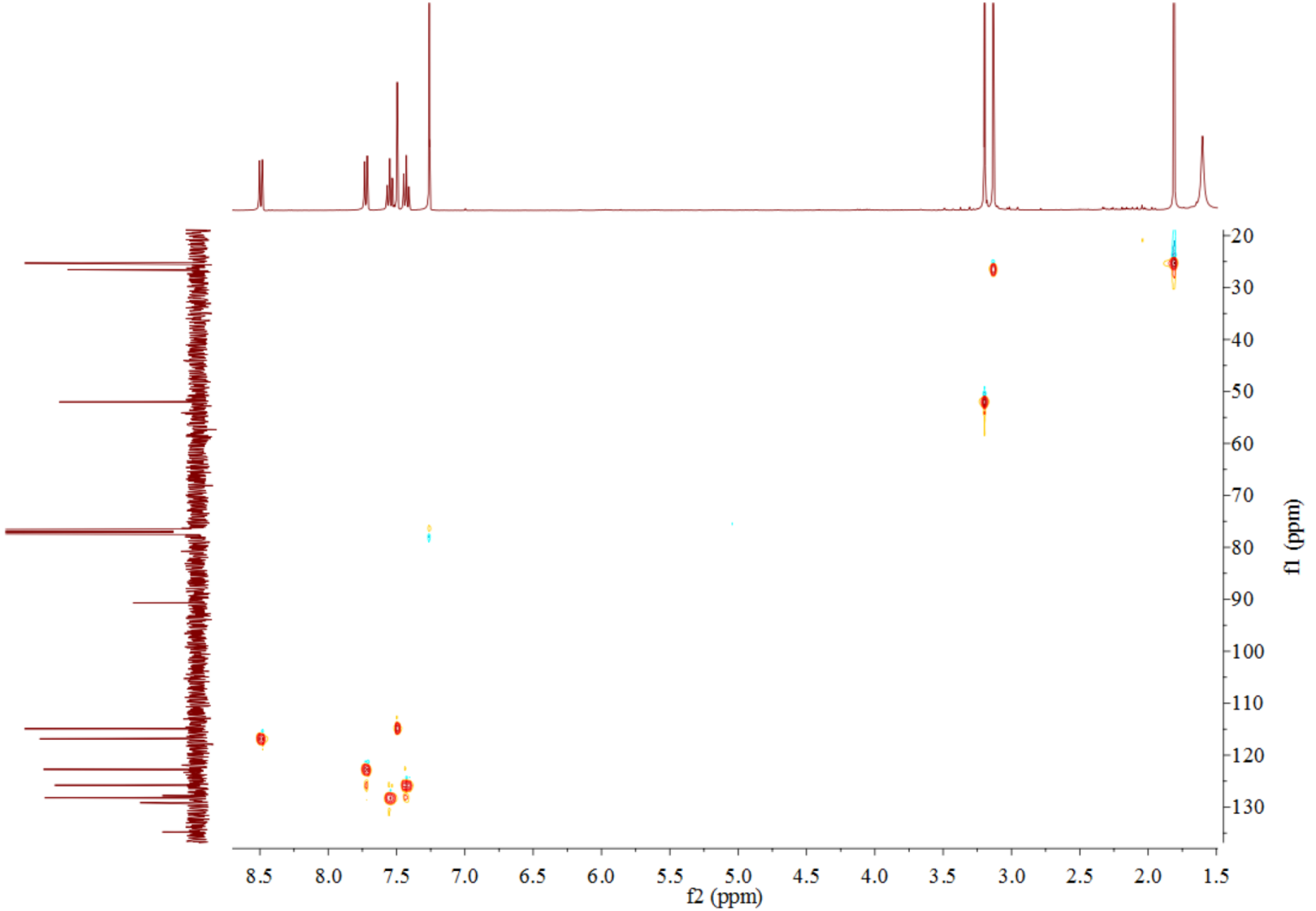

Figure S7. ^{1}H-^{1}H gCOSY of neosartin A (**1**).

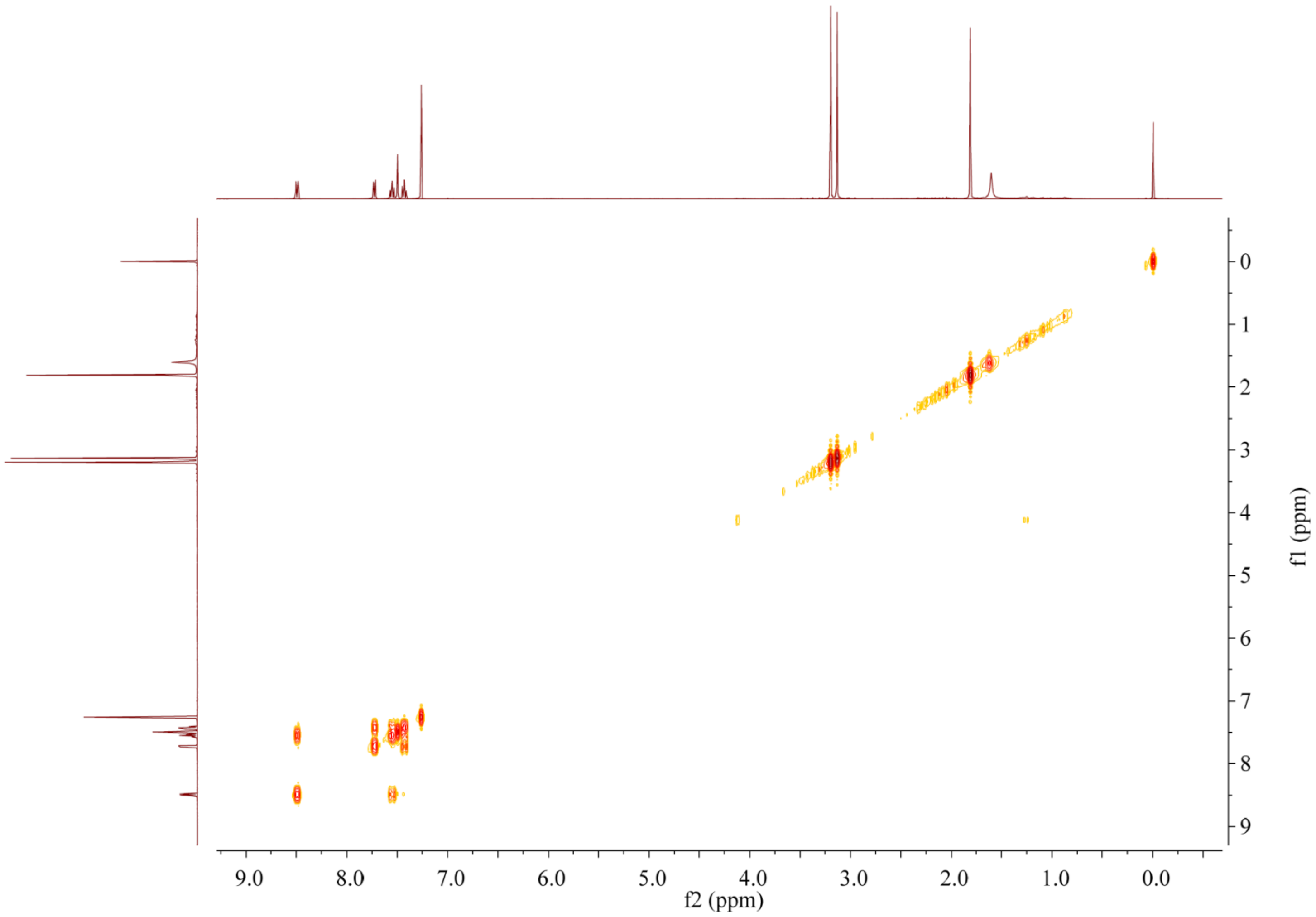

Figure S8. gHMBC of neosartin A (**1**).

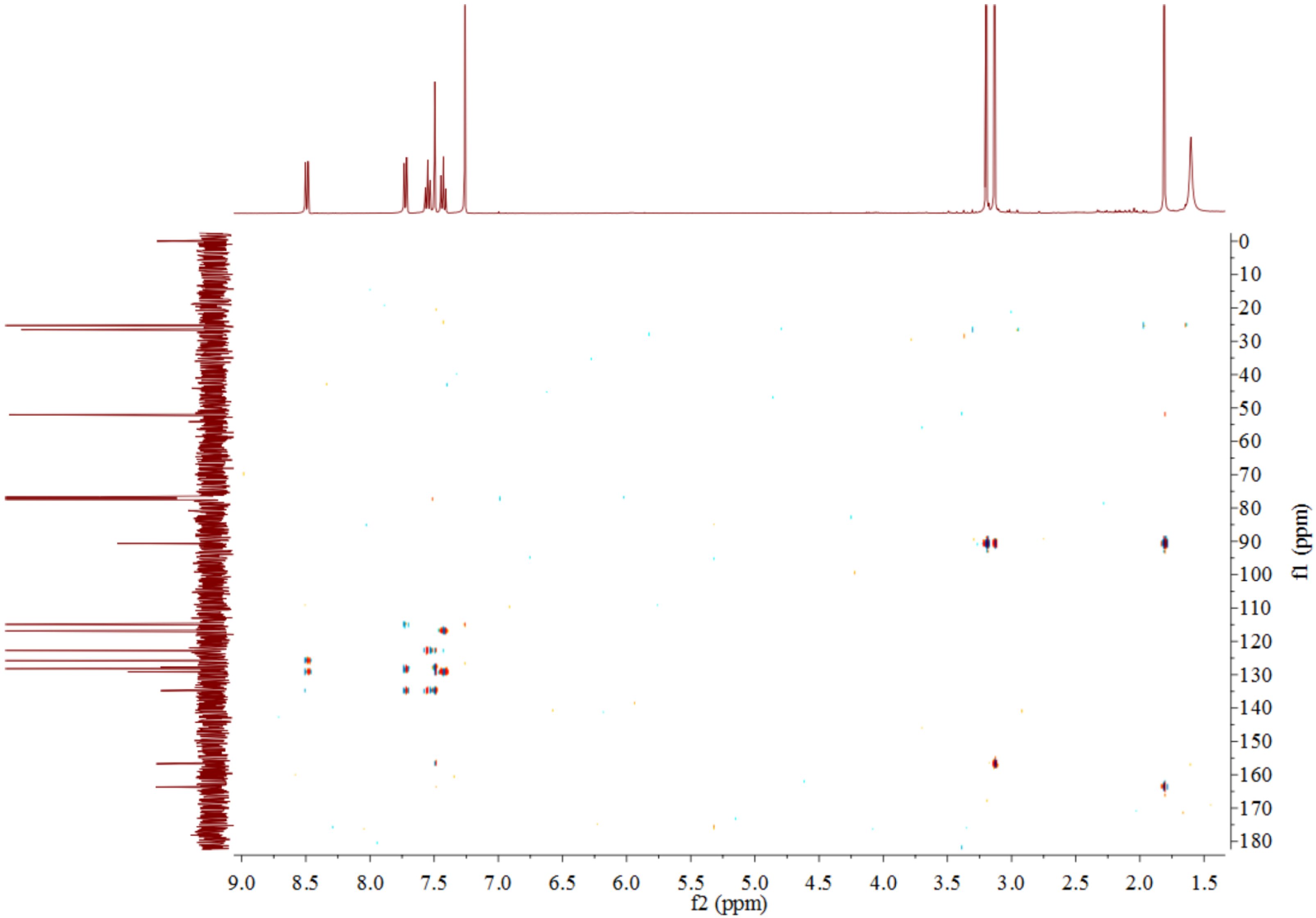

Figure S9. NOESY of neosartin A (**1**).

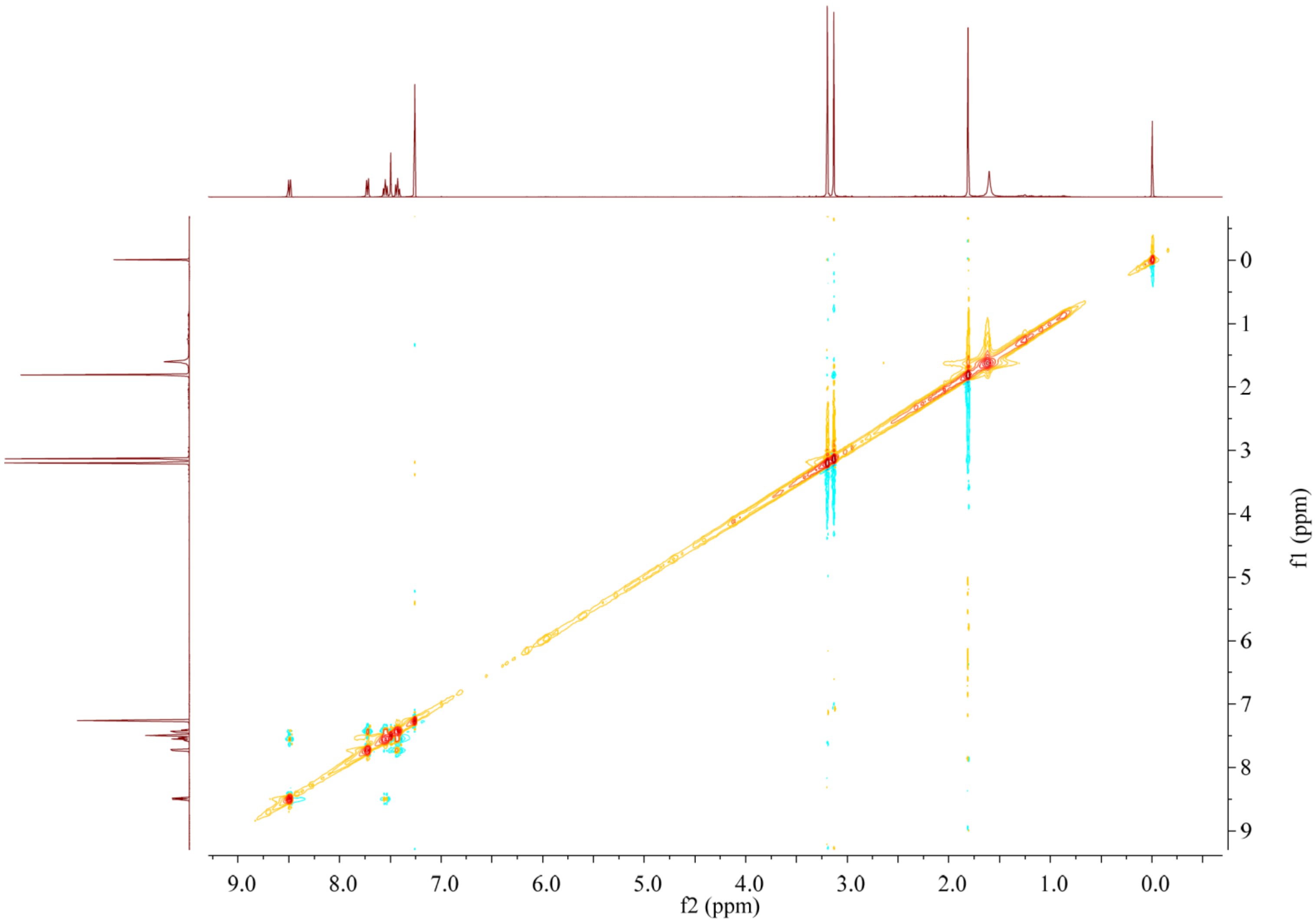

Figure S10. LREIMS of neosartin B (**2**).

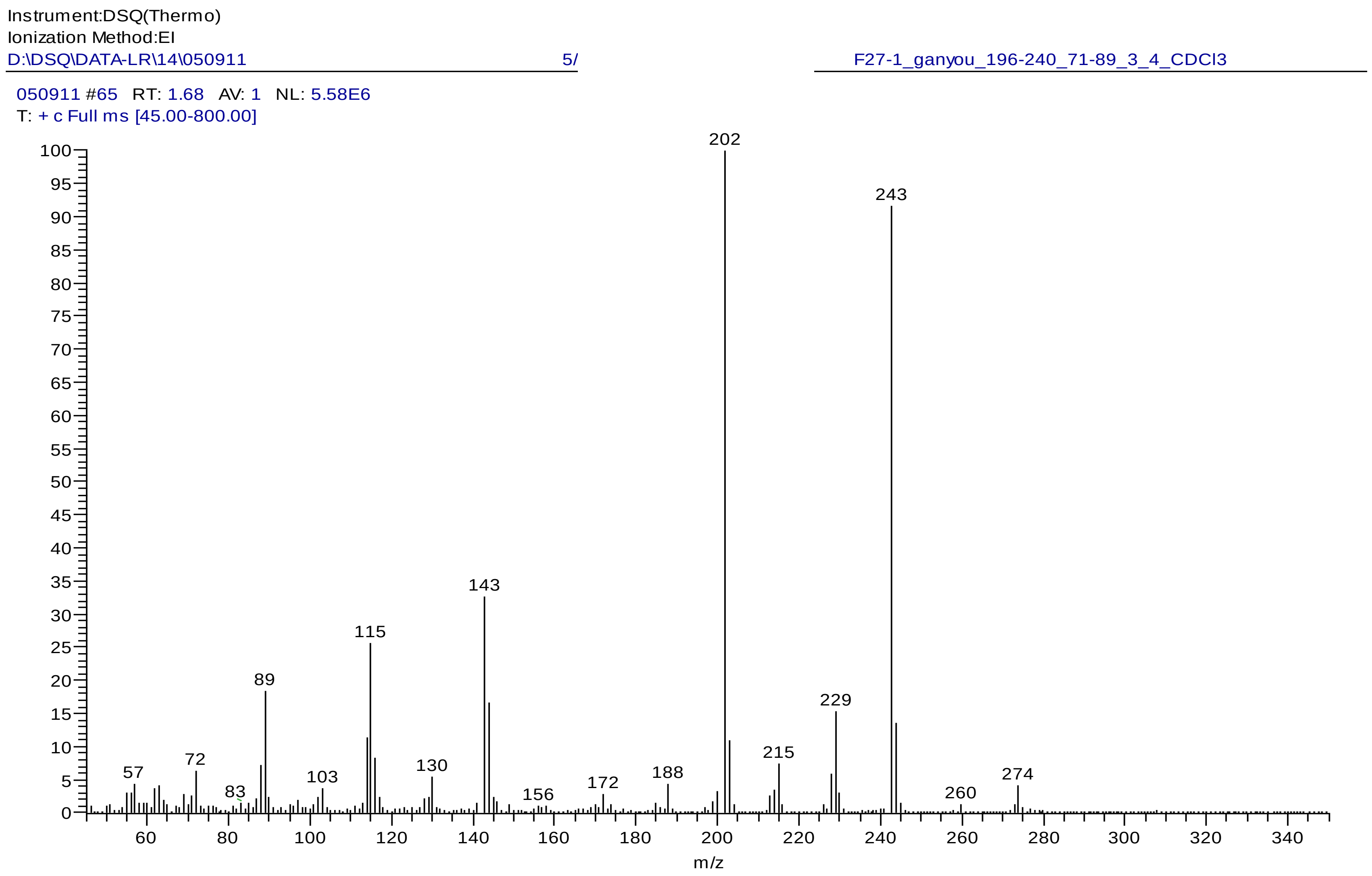

Figure S11. HREIMS of neosartin B (**2**).

SPECTRUM - MS
File : D:\DATA-HR\14\051301-f27-c3.RAW
Full ms [266.500 - 283.500] - Range: 274.000 - 274.300
Scan No. 15 of 26
Scan #: 15
RT: 0.56
Data points: 1

Mass	Relative Intensity	Theoretical Mass	Delta [ppm]	Delta [mmu]	RDB	Composition
274.0947	18.4	274.0948	-0.4	-0.1	9.0	$C_{14} H_{14} O_4 N_2$

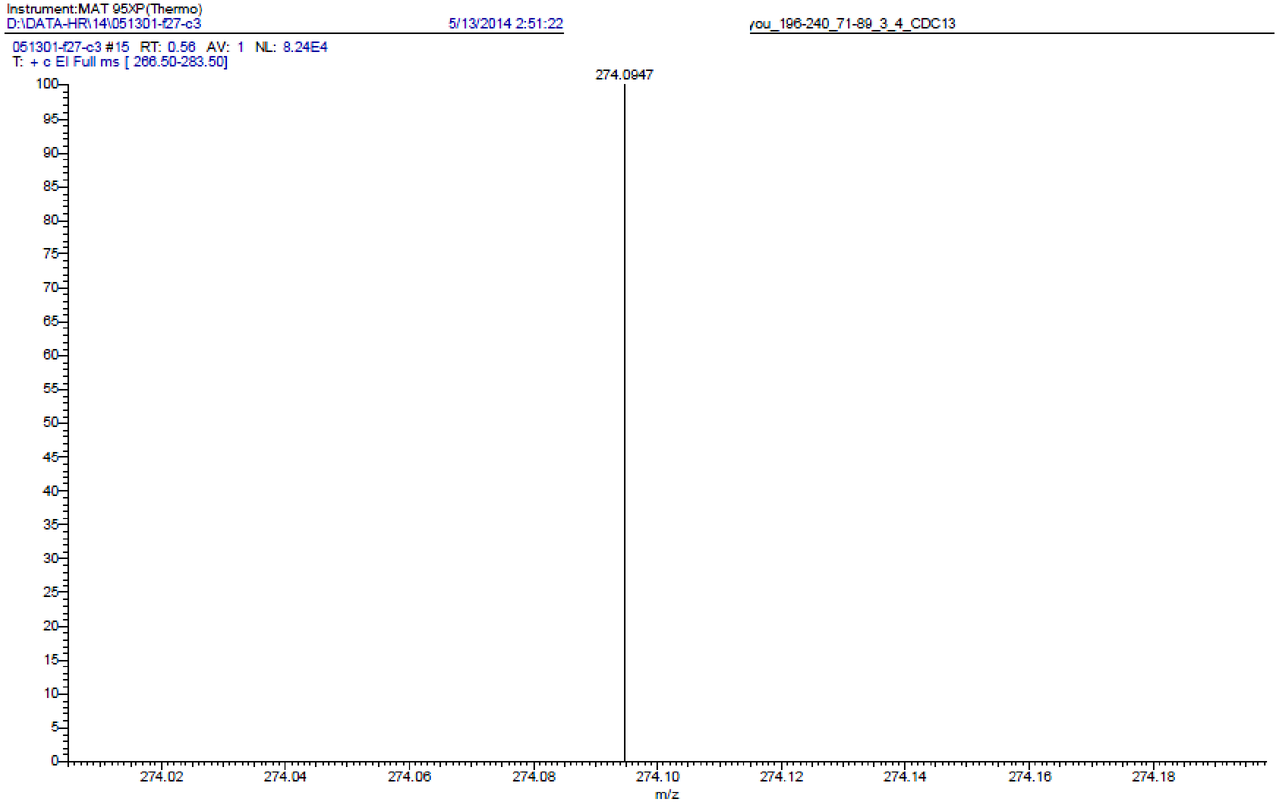

Figure S12. ^{1}H NMR (400 MHz, $CDCl_3$) spectrum of neosartin B (**2**).

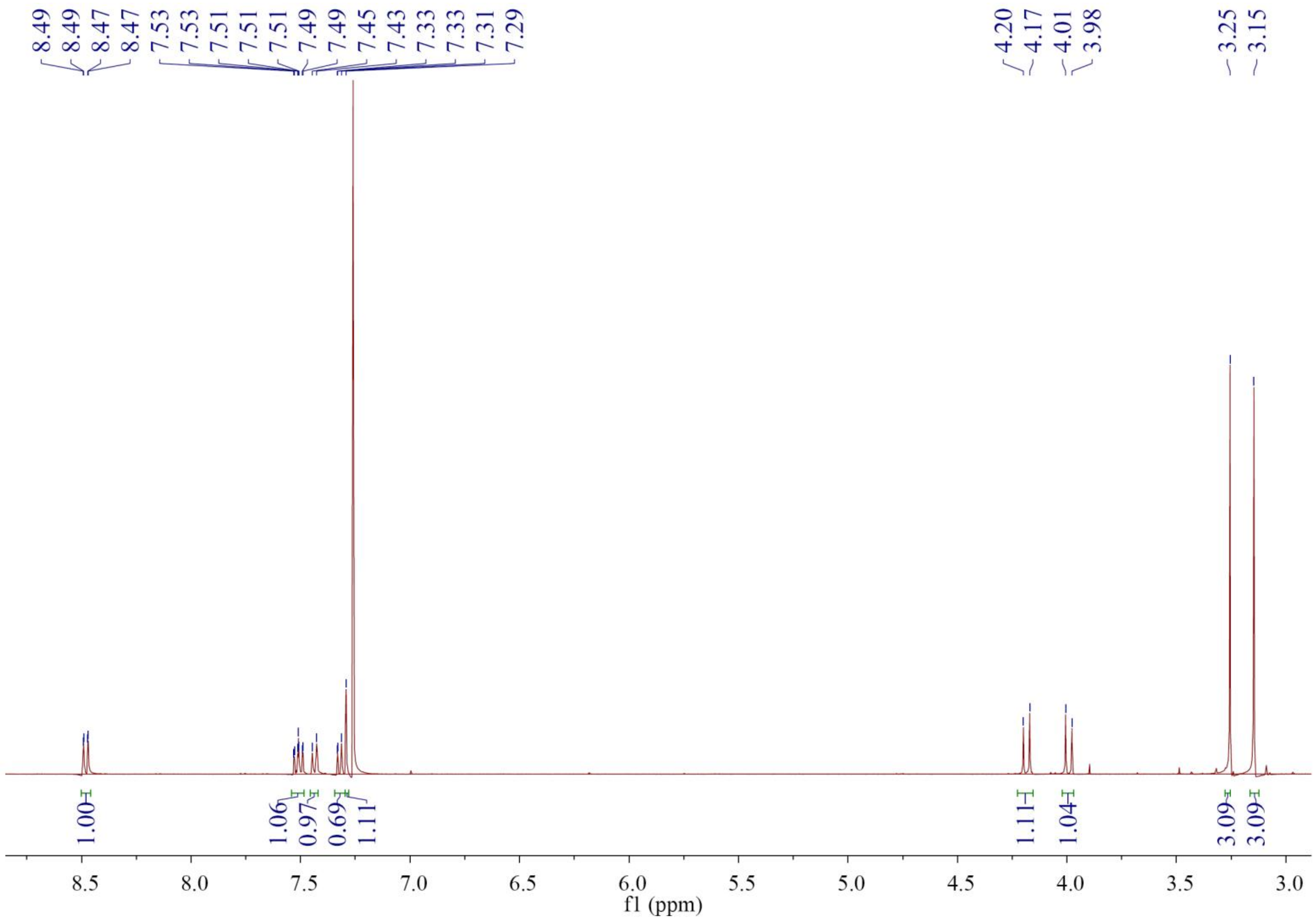

Figure S13. ^{13}C NMR (100 MHz, $CDCl_3$) spectrum of neosartin B (**2**).

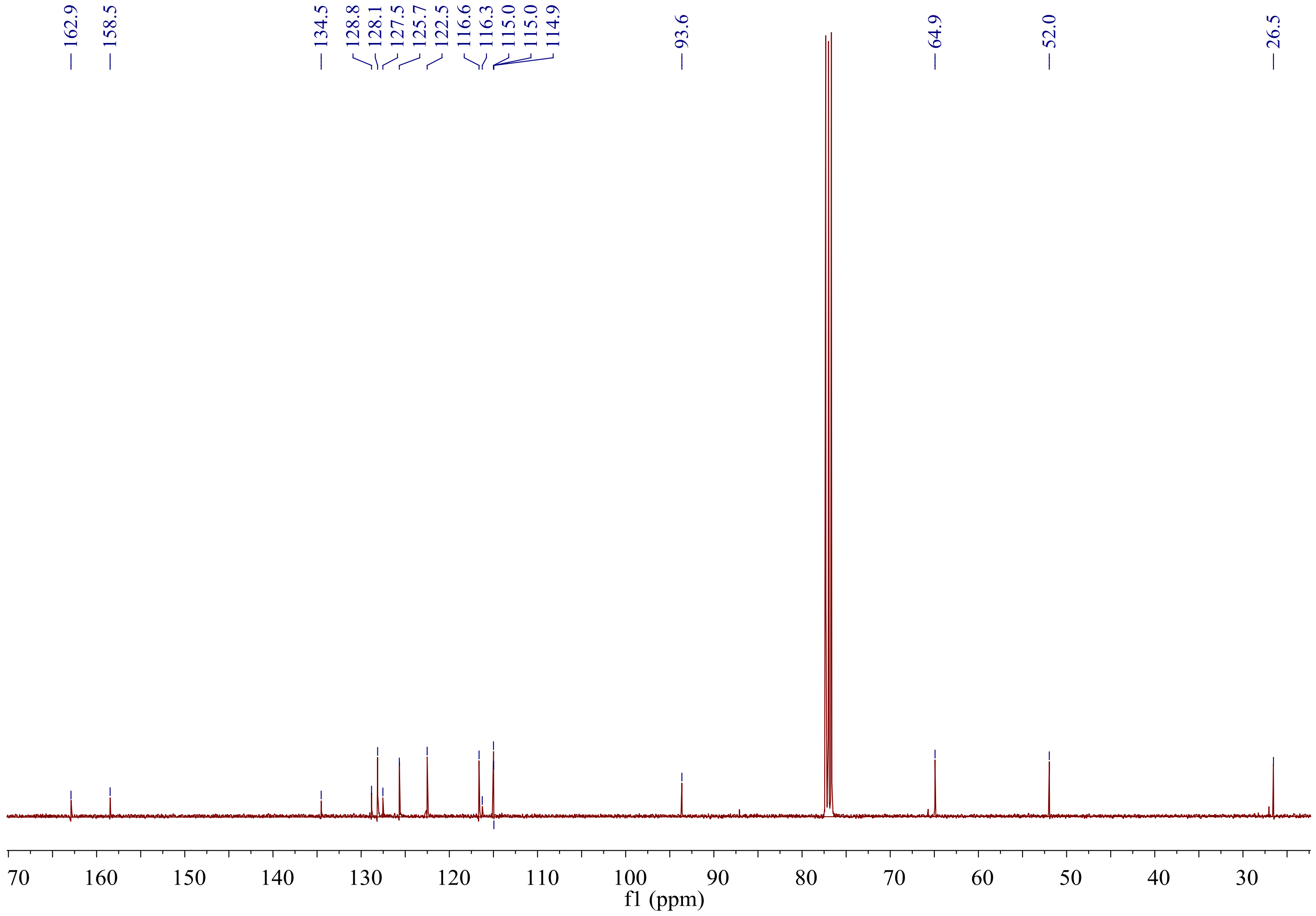

Figure S14. gHMQC spectrum of neosartin B (**2**).

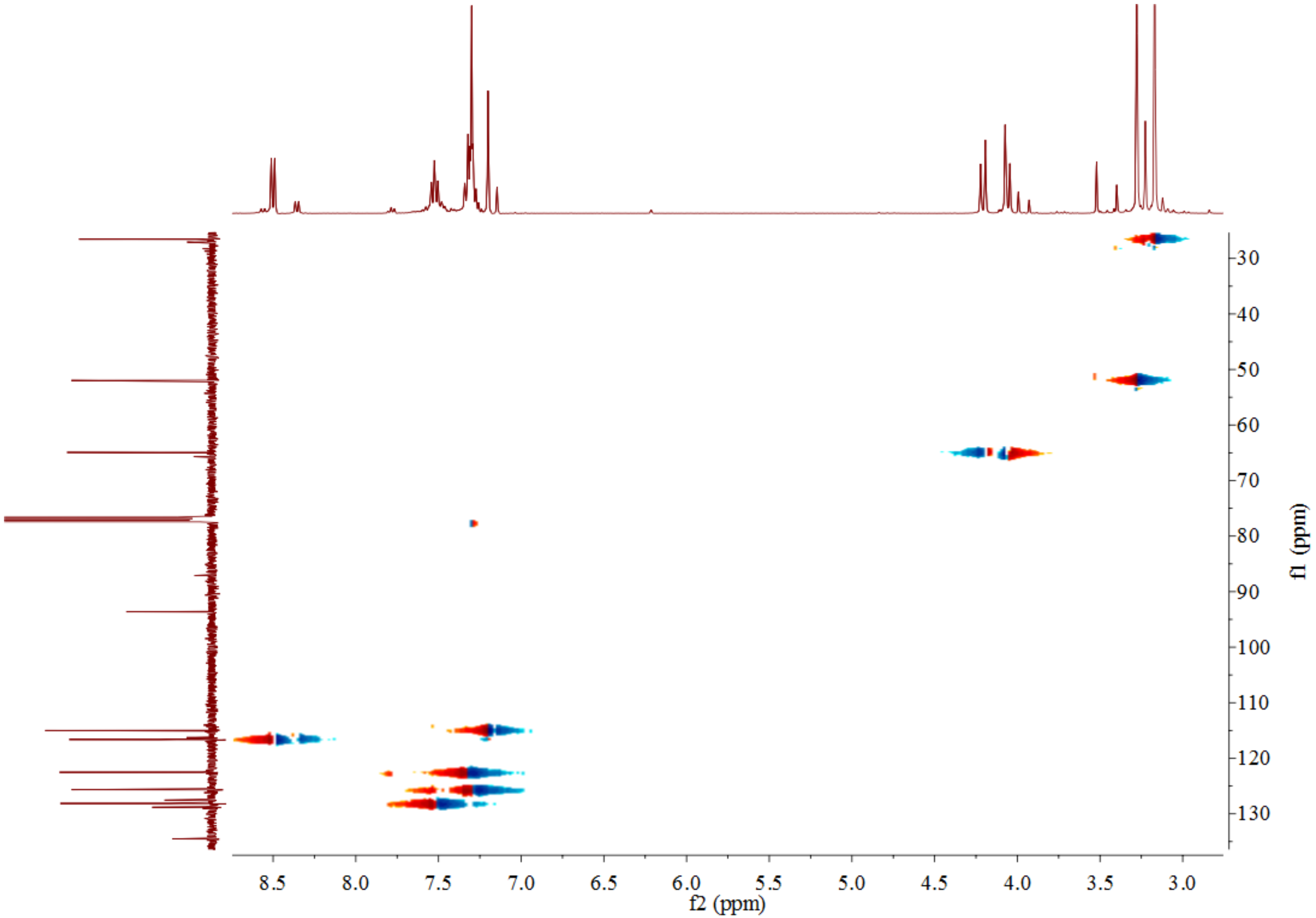

Figure S15. ^1H-^1H gCOSY spectrum of neosartin B (**2**).

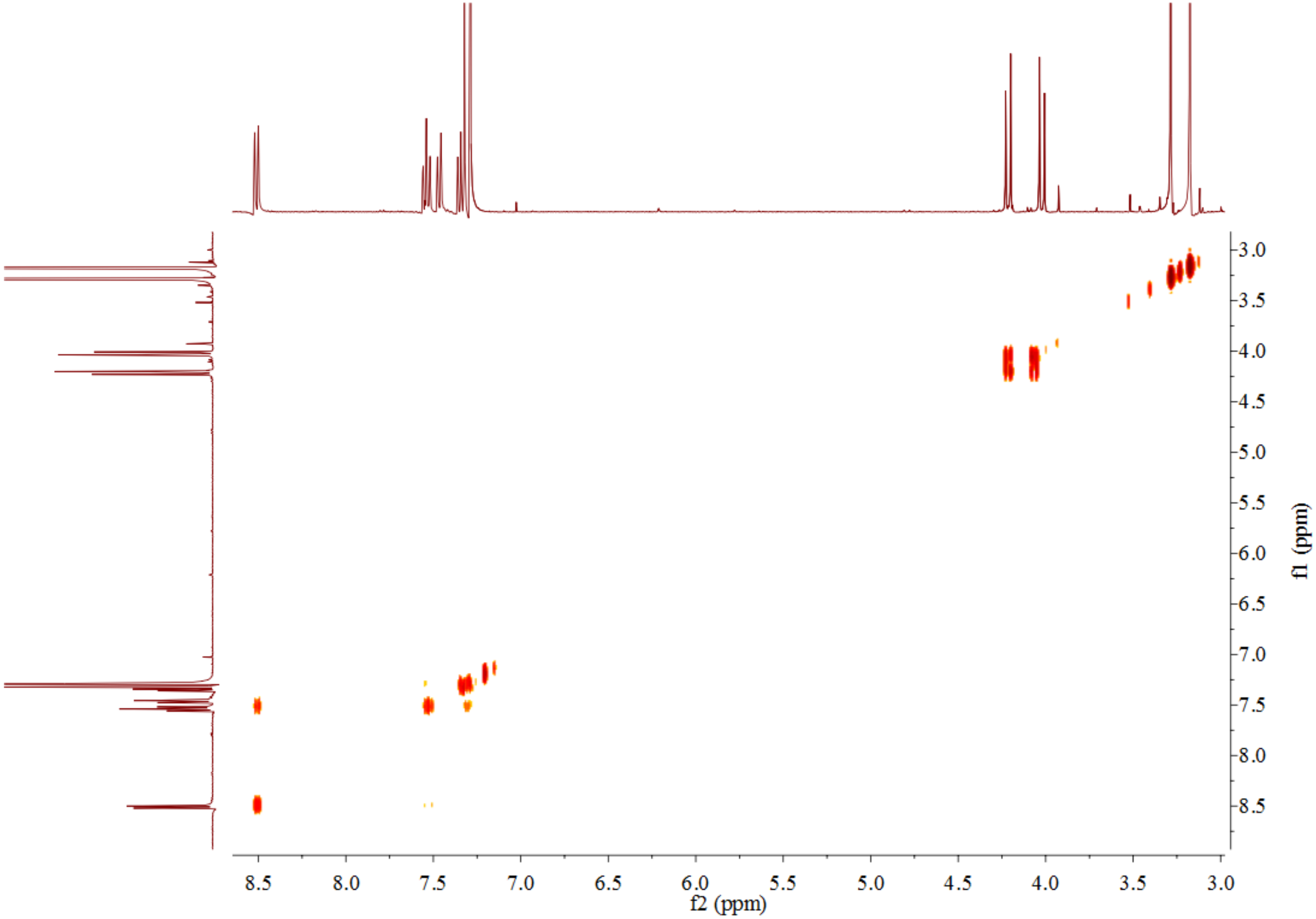

Figure S16. gHMBC spectrum of neosartin B (**2**).

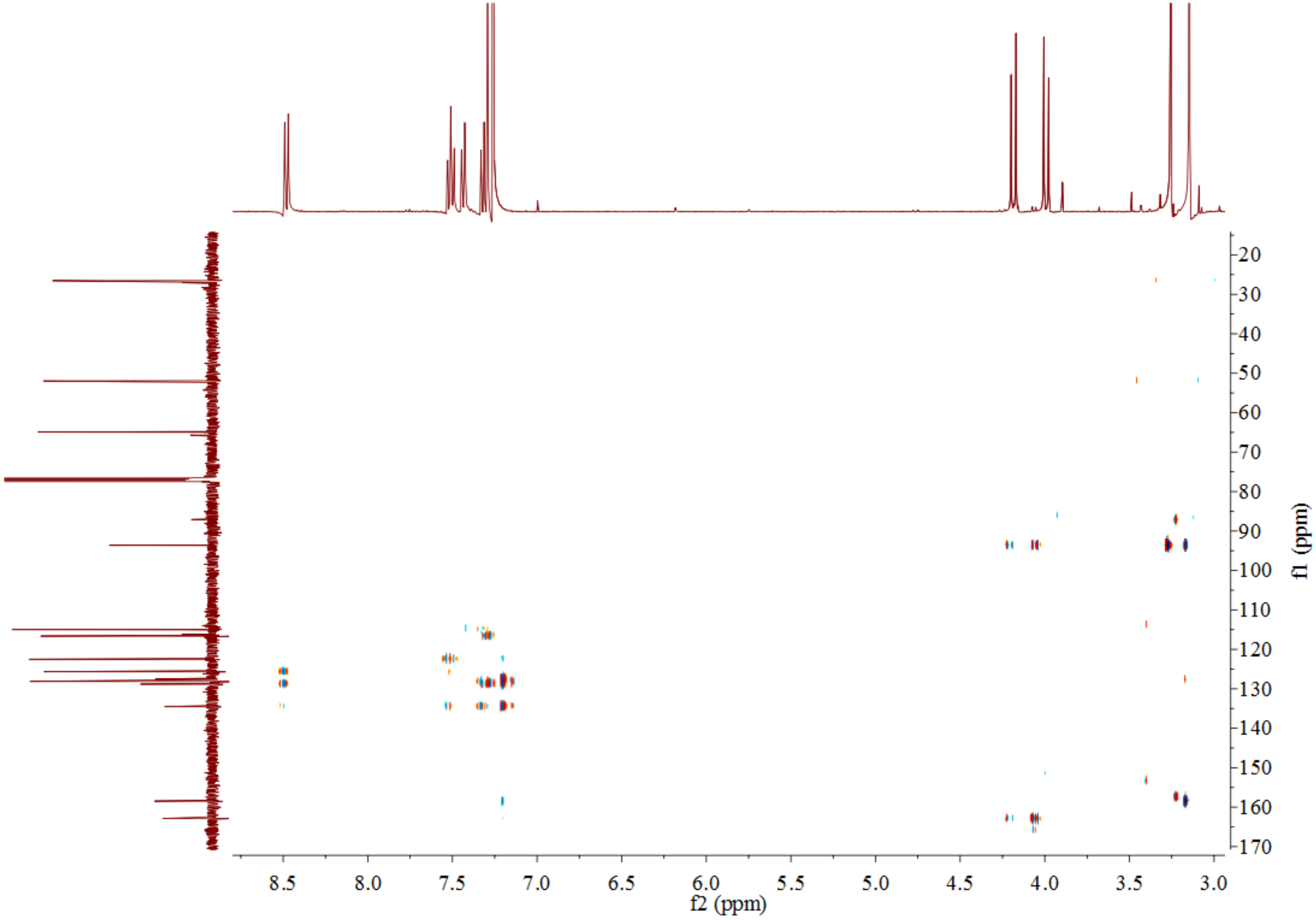

Figure S17. NOESY of neosartin B (**2**).

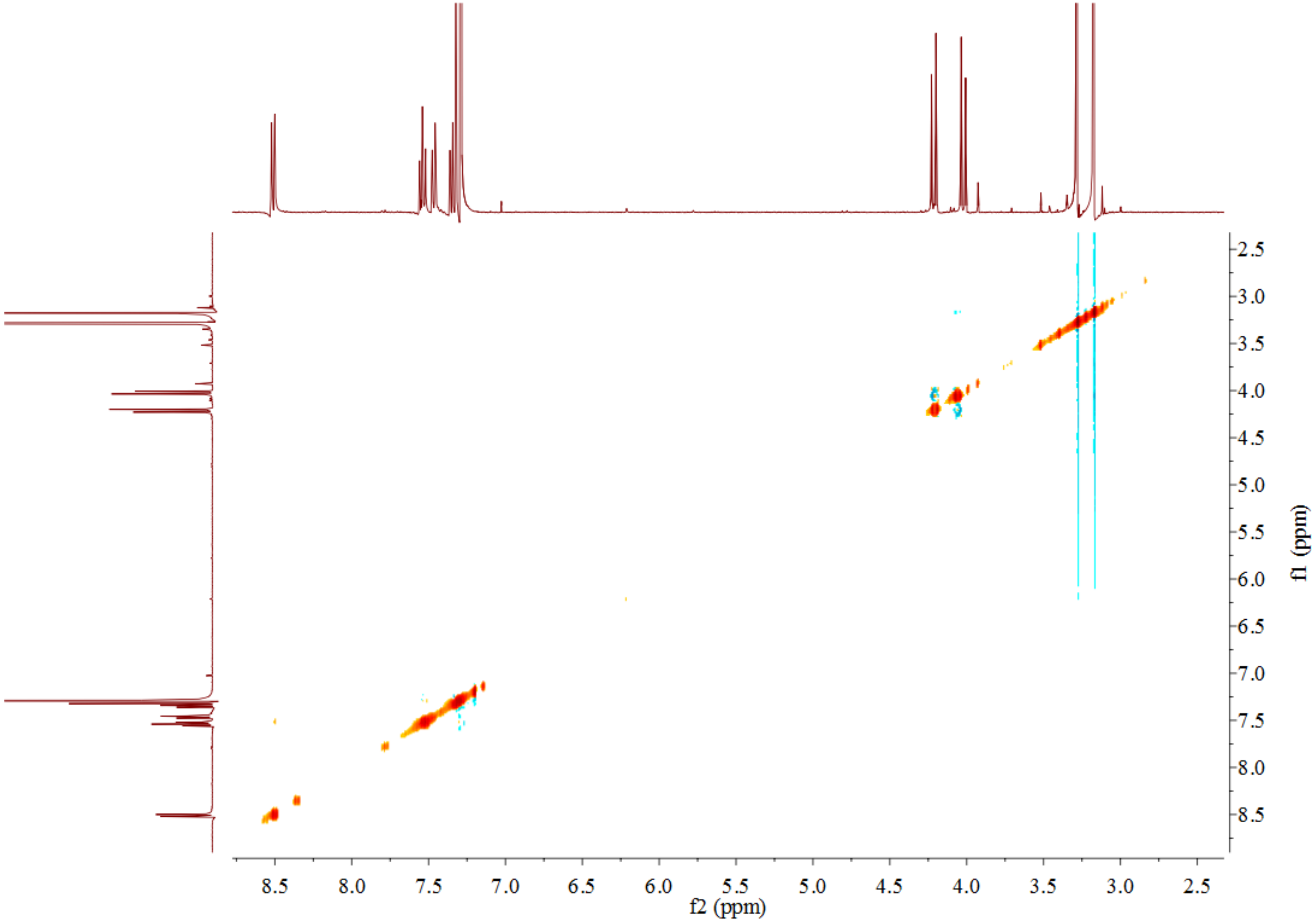

Figure S18. LREIMS of 1,2,3,4-tetrahydro-2,3-dimethyl-1,4-dioxopyrazino[1,2-a]indole (**3**).

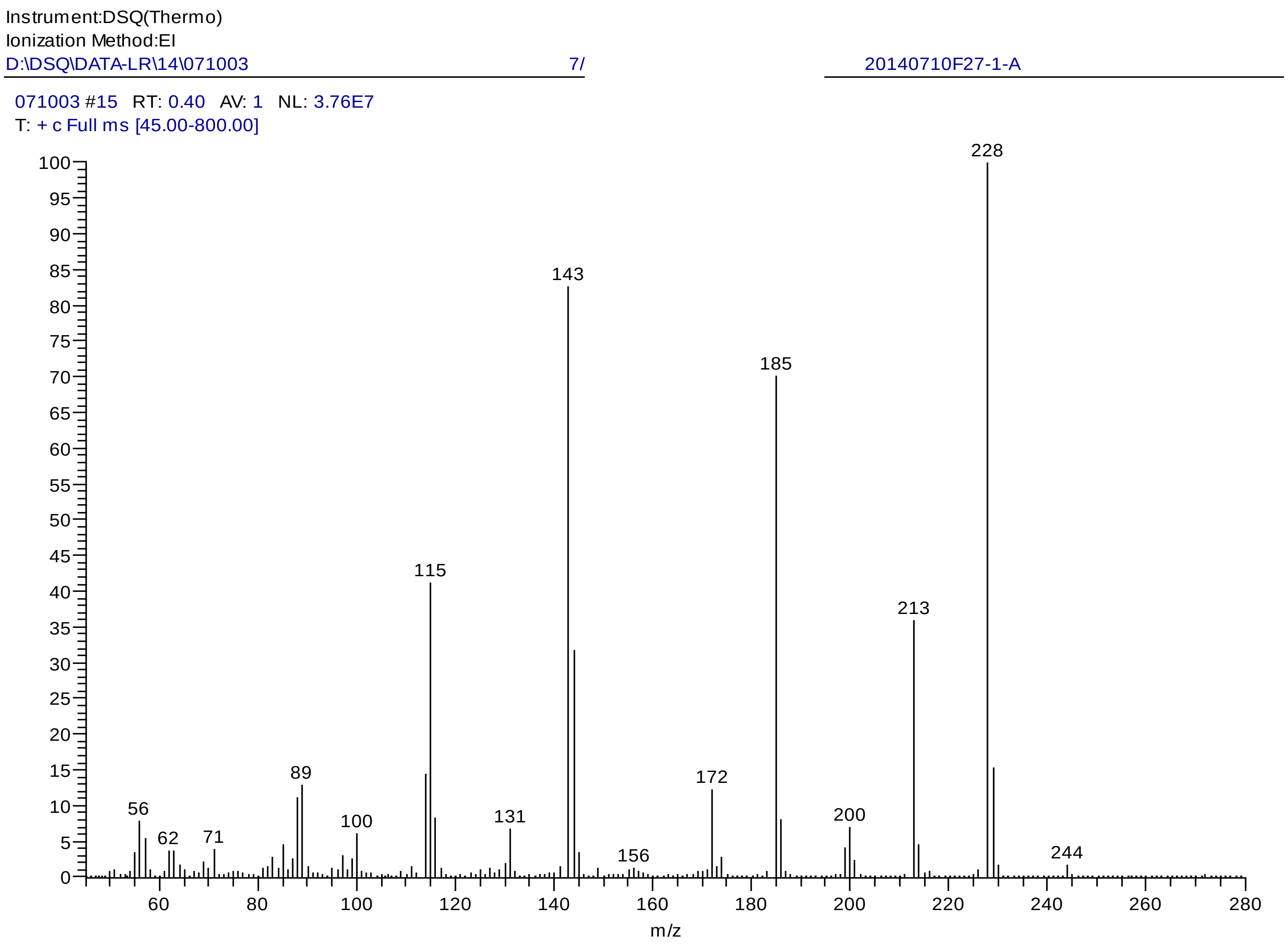

Figure S19. ^{1}H NMR (400 MHz, $CDCl_3$) spectrum of 1,2,3,4-tetrahydro-2,3-dimethyl-1,4-dioxopyrazino[1,2-a]indole (**3**).

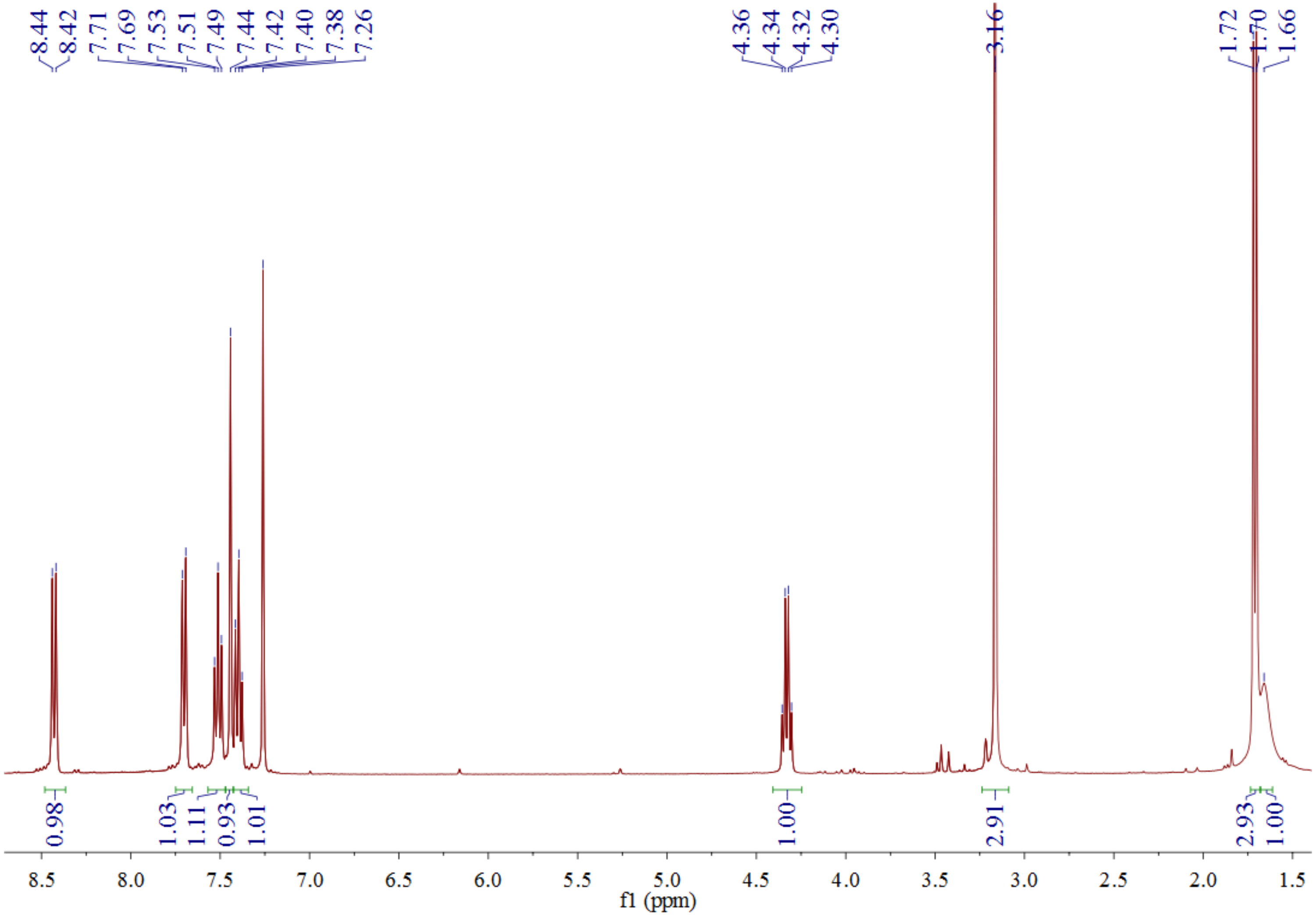

Figure S20. ^{13}C NMR (100 MHz, $CDCl_3$) spectrum of 1,2,3,4-tetrahydro-2,3-dimethyl-1,4-dioxopyrazino[1,2-a]indole (**3**).

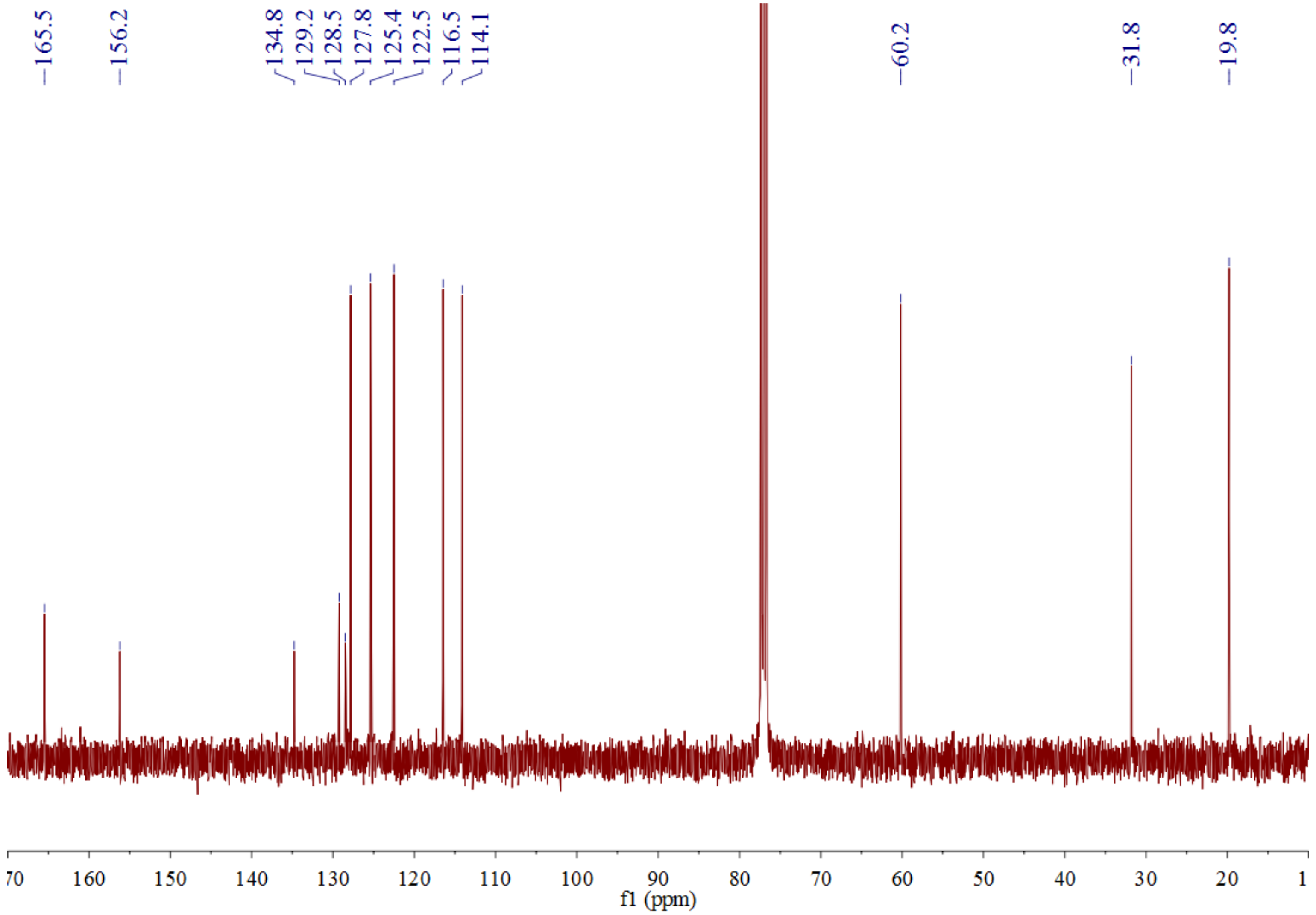

Figure S21. gHMQC spectrum of 1,2,3,4-tetrahydro-2,3-dimethyl-1,4-dioxopyrazino[1,2-a]indole (**3**).

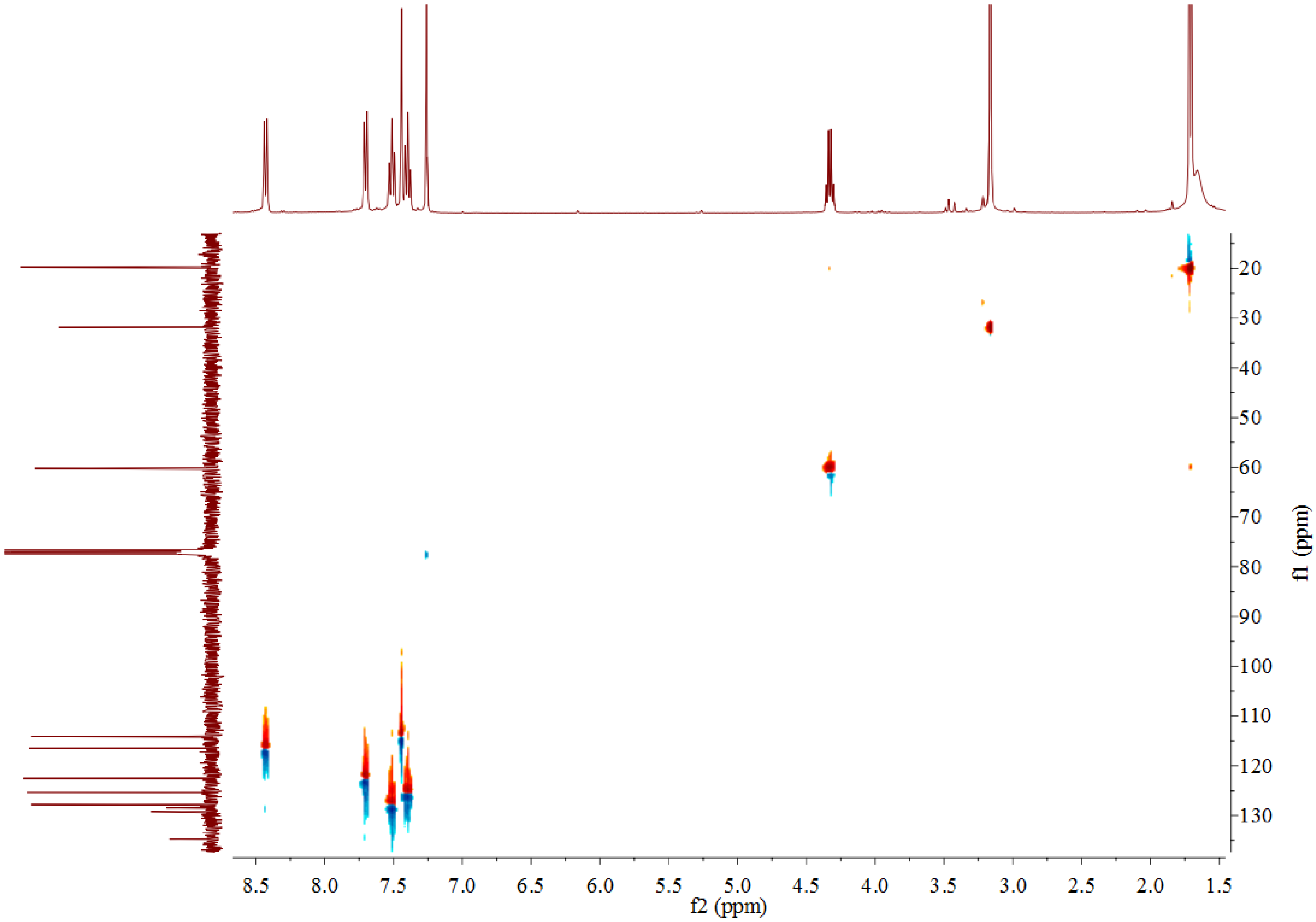

Figure S22. ^{1}H-^{1}H gCOSY spectrum of 1,2,3,4-tetrahydro-2,3-dimethyl-1,4-dioxopyrazino[1,2-a]indole (**3**).

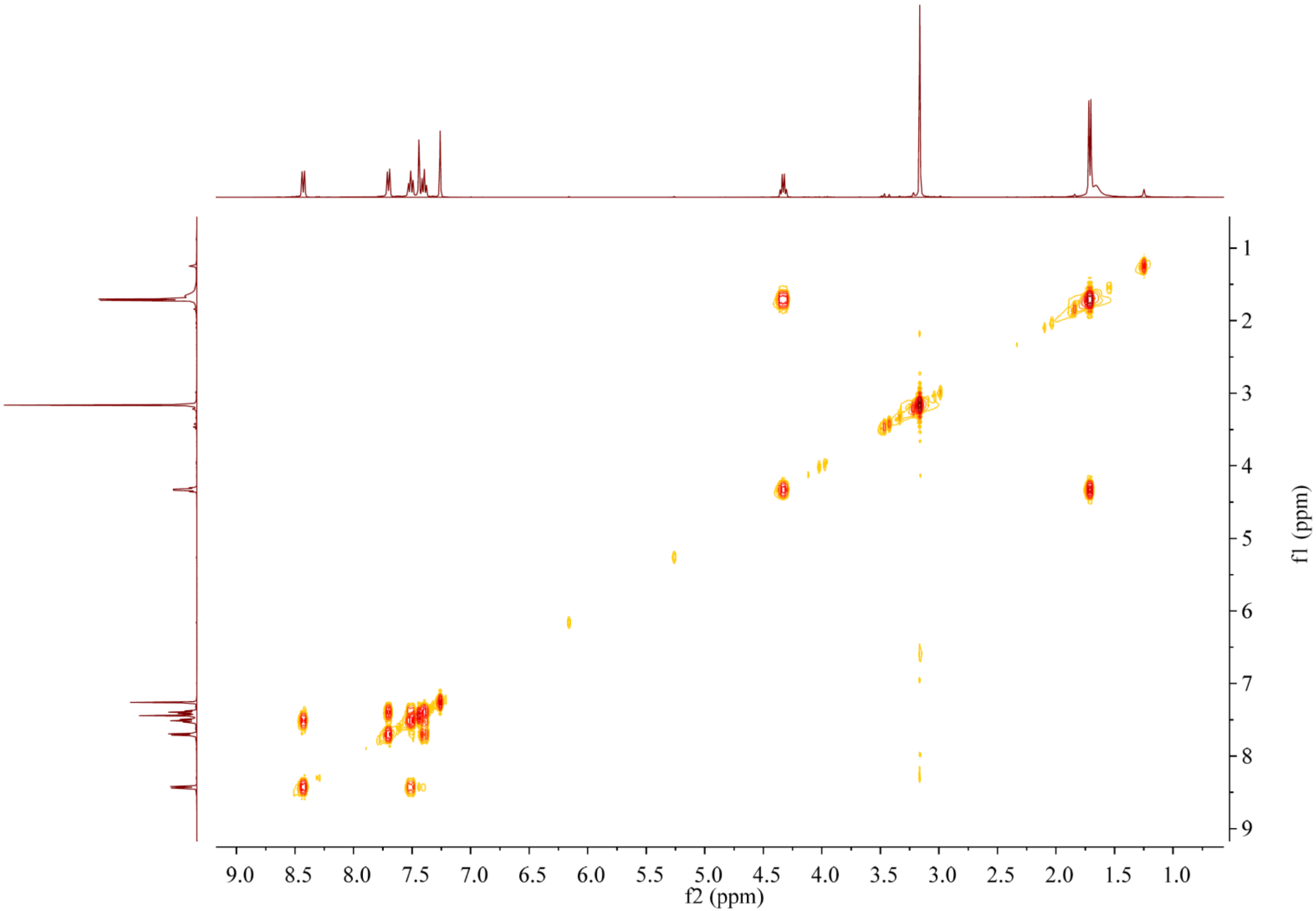

Figure S23. gHMBC spectrum of 1,2,3,4-tetrahydro-2,3-dimethyl-1,4-dioxopyrazino[1,2-a]indole (**3**).

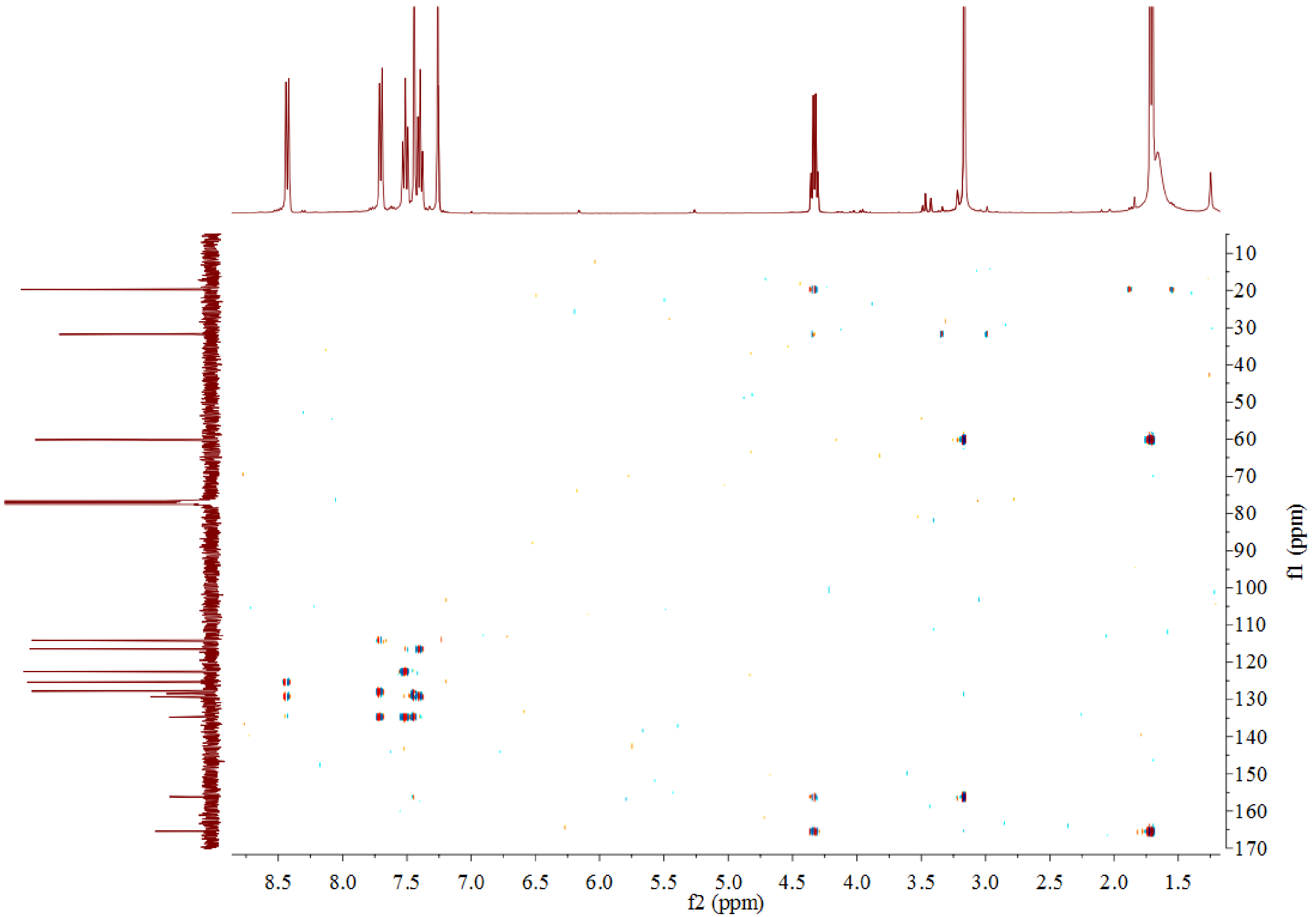

Figure S24. NOESY of 1,2,3,4-tetrahydro-2,3-dimethyl-1,4-dioxopyrazino[1,2-a]indole (**3**).

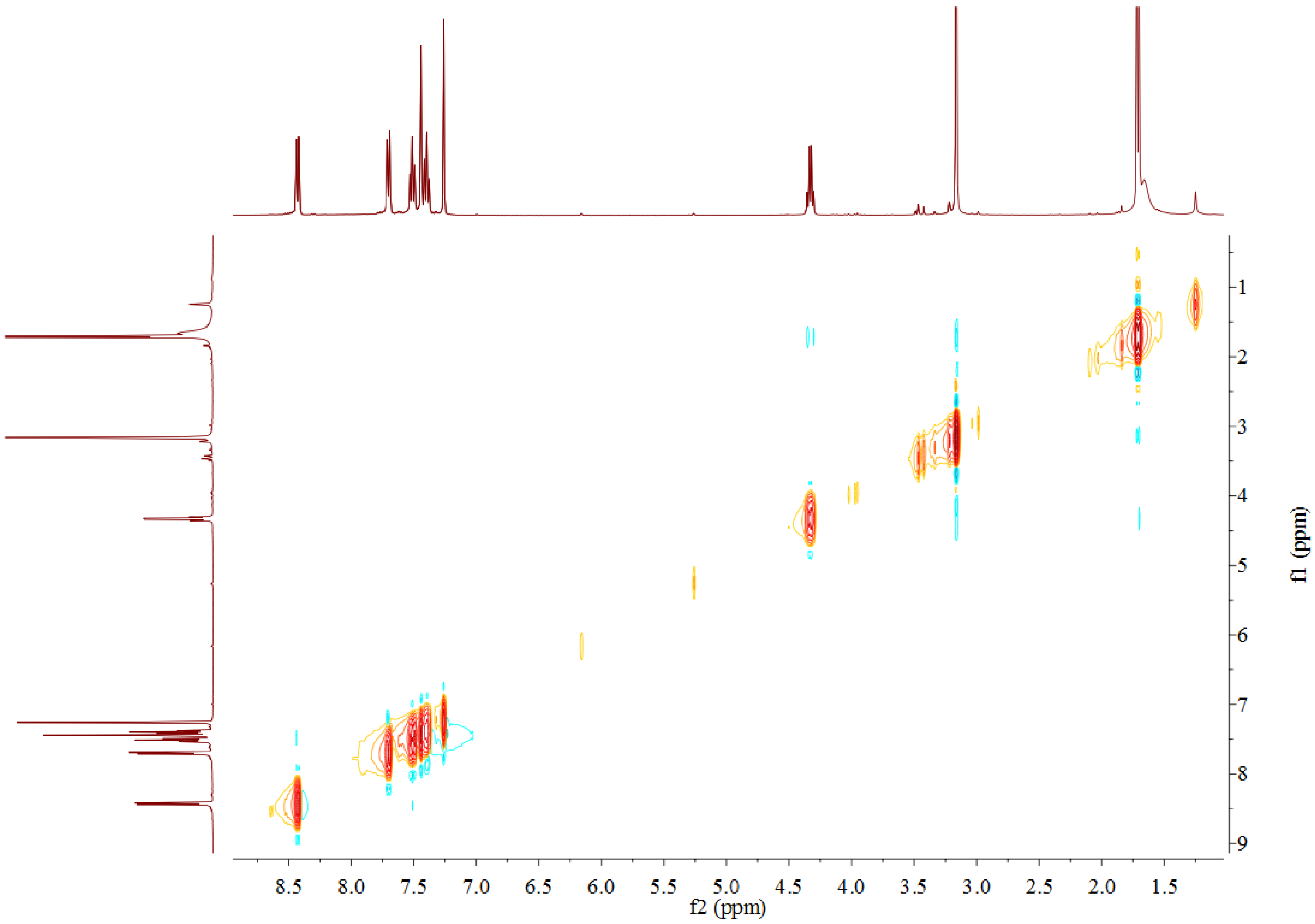

Figure S25. LREIMS of 1,2,3,4-tetrahydro-2-methyl-3-methylene-1,4-dioxopyrazino[1,2-a]indole (**4**).

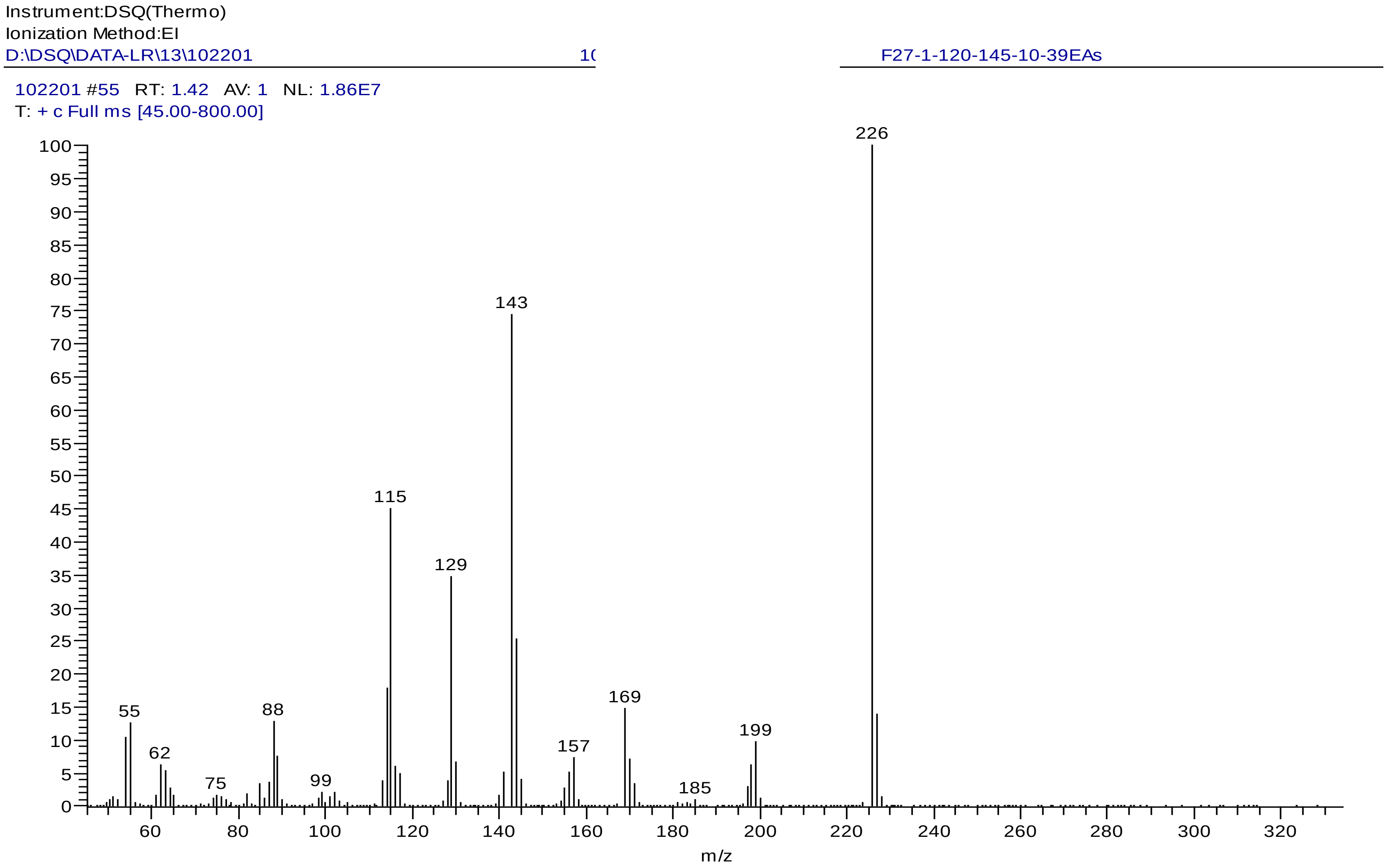

Figure S26. ^{1}H NMR (400 MHz, $CDCl_3$) spectrum of 1,2,3,4-tetrahydro-2-methyl-3-methylene-1,4-dioxopyrazino[1,2-a]indole (**4**).

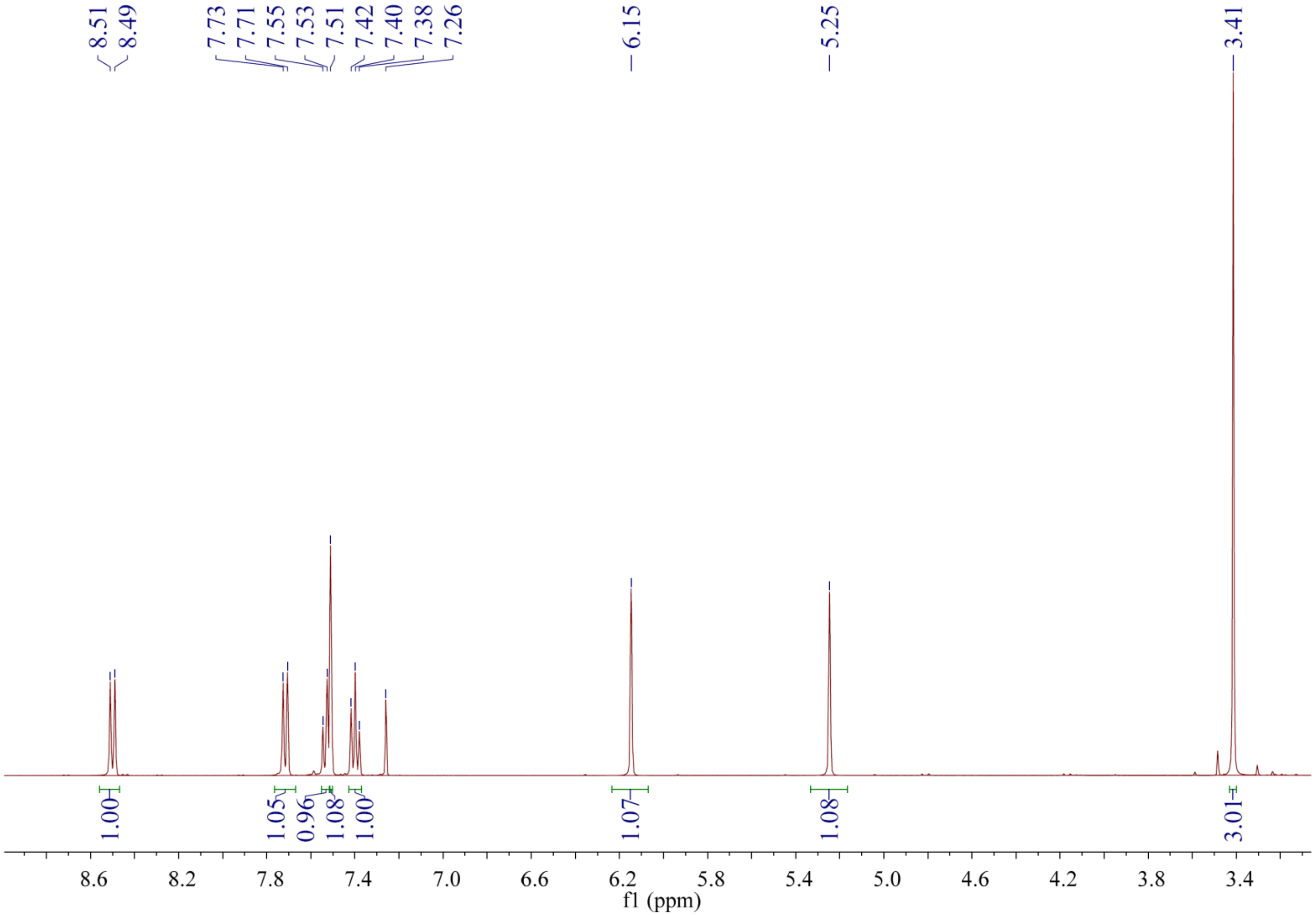

Figure S27. ^{13}C NMR (100 MHz, $CDCl_3$) spectrum of 1,2,3,4-tetrahydro-2-methyl-3-methylene-1,4-dioxopyrazino[1,2-a]indole (**4**).

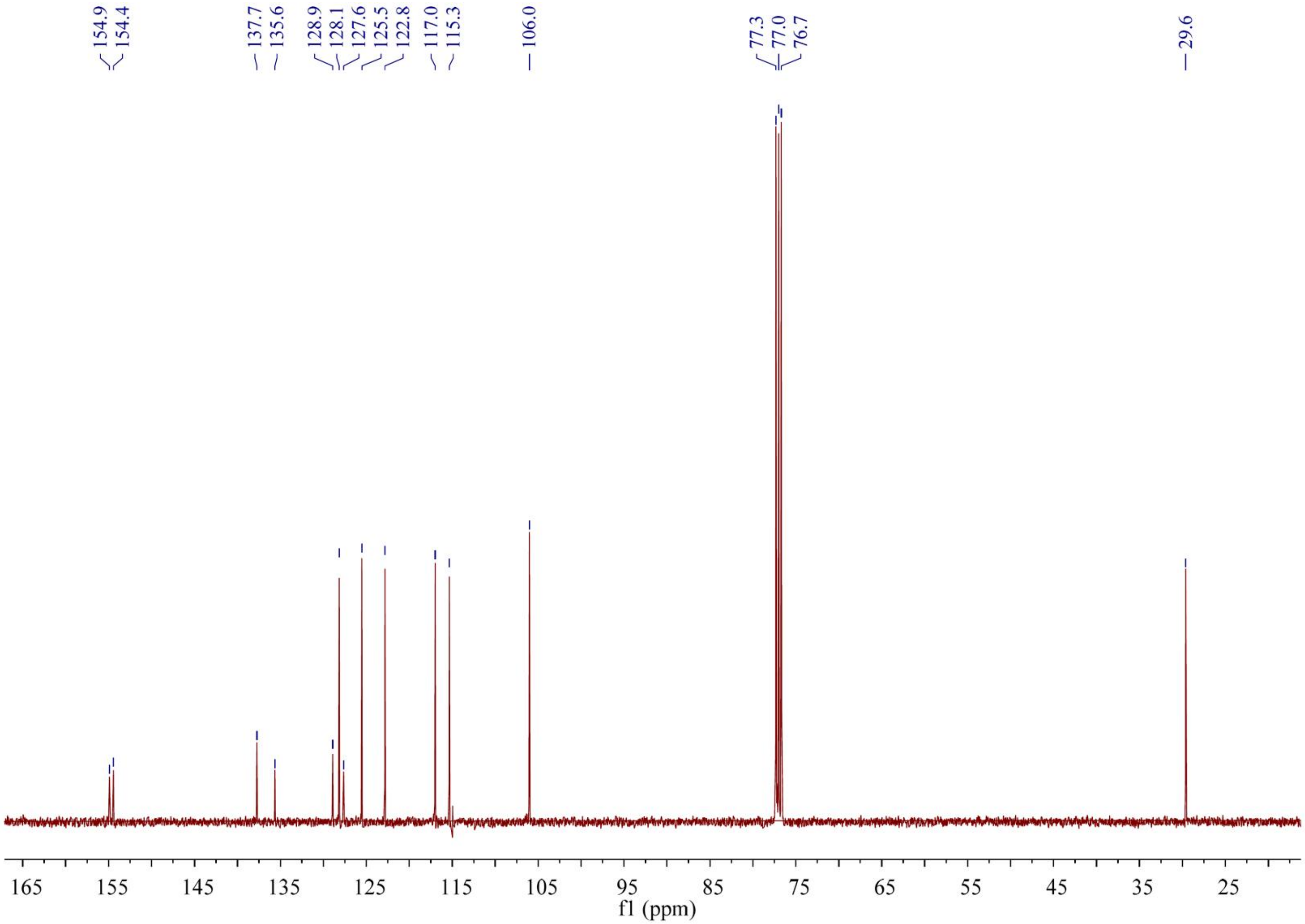

Figure S28. gHMQC spectrum of 1,2,3,4-tetrahydro-2-methyl-3-methylene-1,4-dioxopyrazino[1,2-a]indole (**4**).

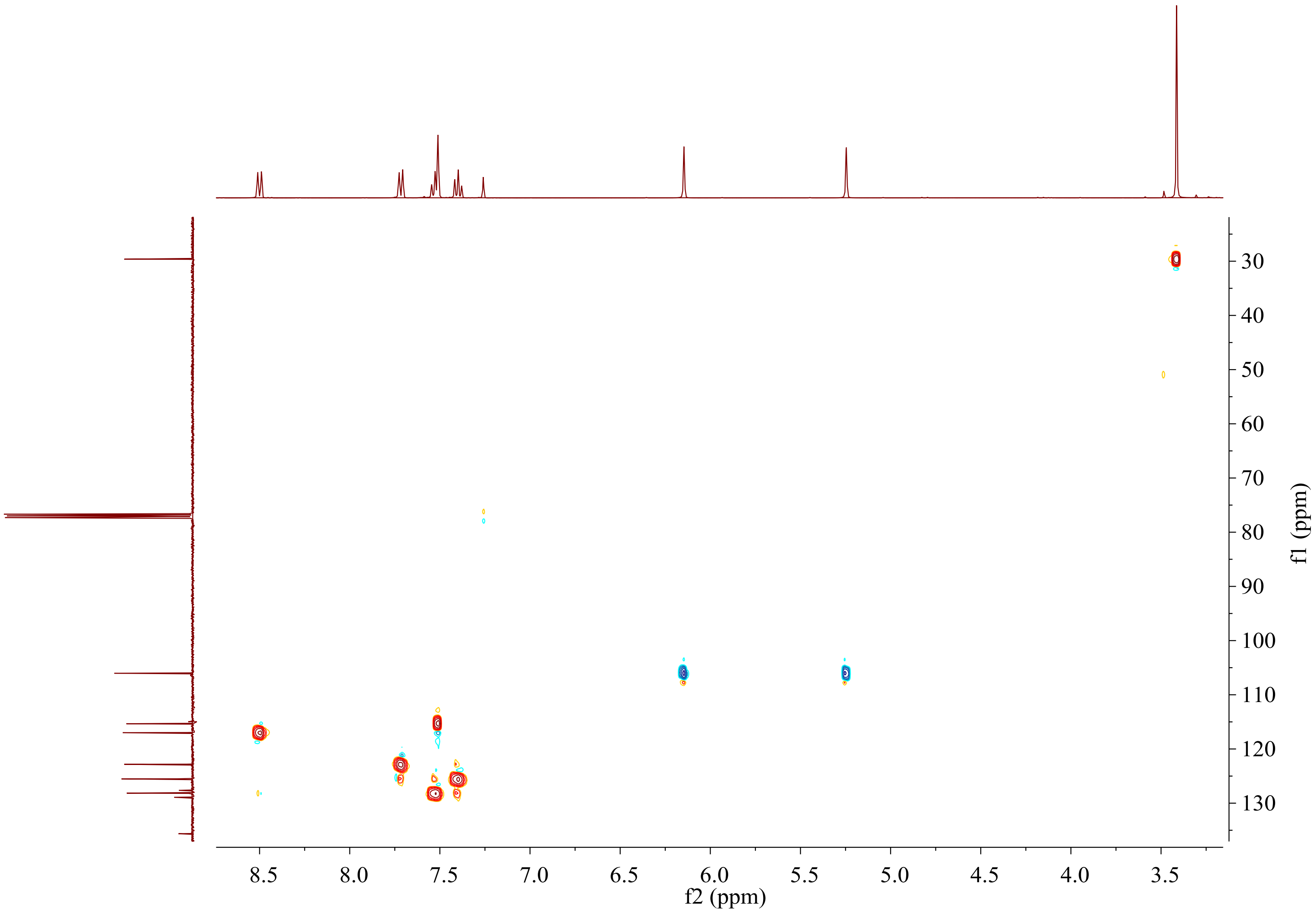

Figure S29. ^{1}H-^{1}H gCOSY spectrum of 1,2,3,4-tetrahydro-2-methyl-3-methylene-1,4-dioxopyrazino[1,2-a]indole (**4**).

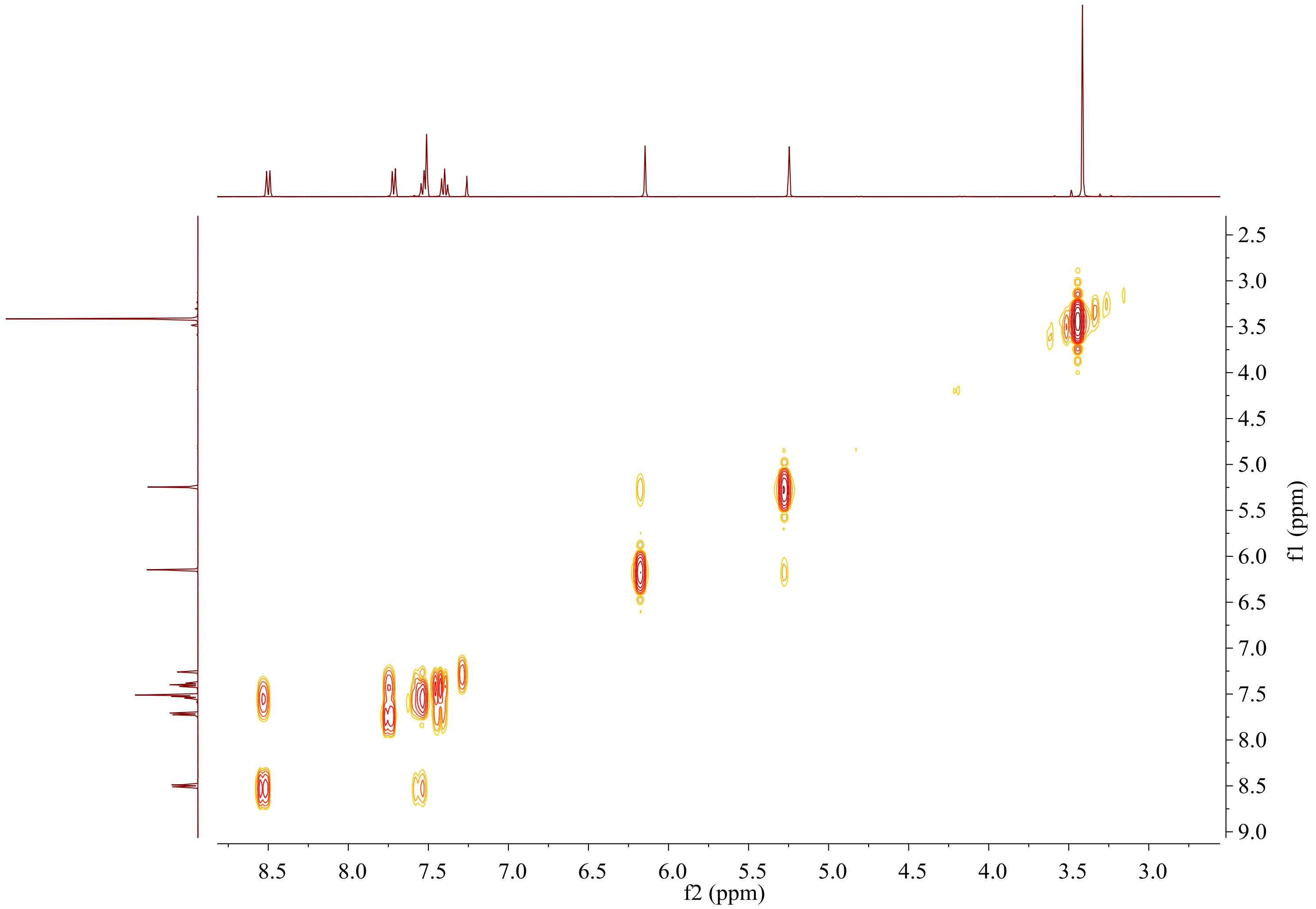

Figure S30. gHMBC spectrum of 1,2,3,4-tetrahydro-2-methyl-3-methylene-1,4-dioxopyrazino[1,2-a]indole (**4**).

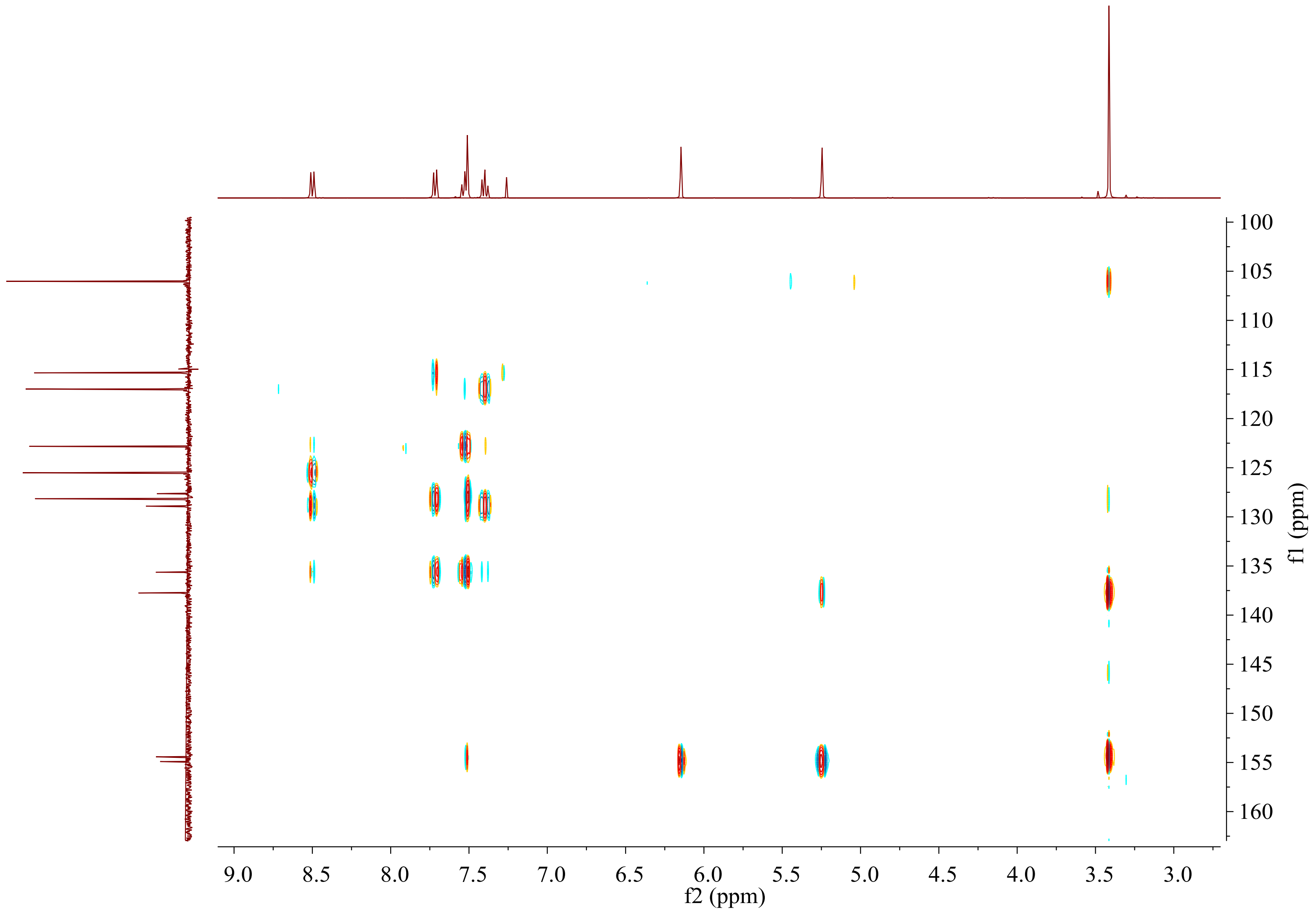

Figure S31. NOESY of 1,2,3,4-tetrahydro-2-methyl-3-methylene-1,4-dioxopyrazino[1,2-a]indole (**4**).

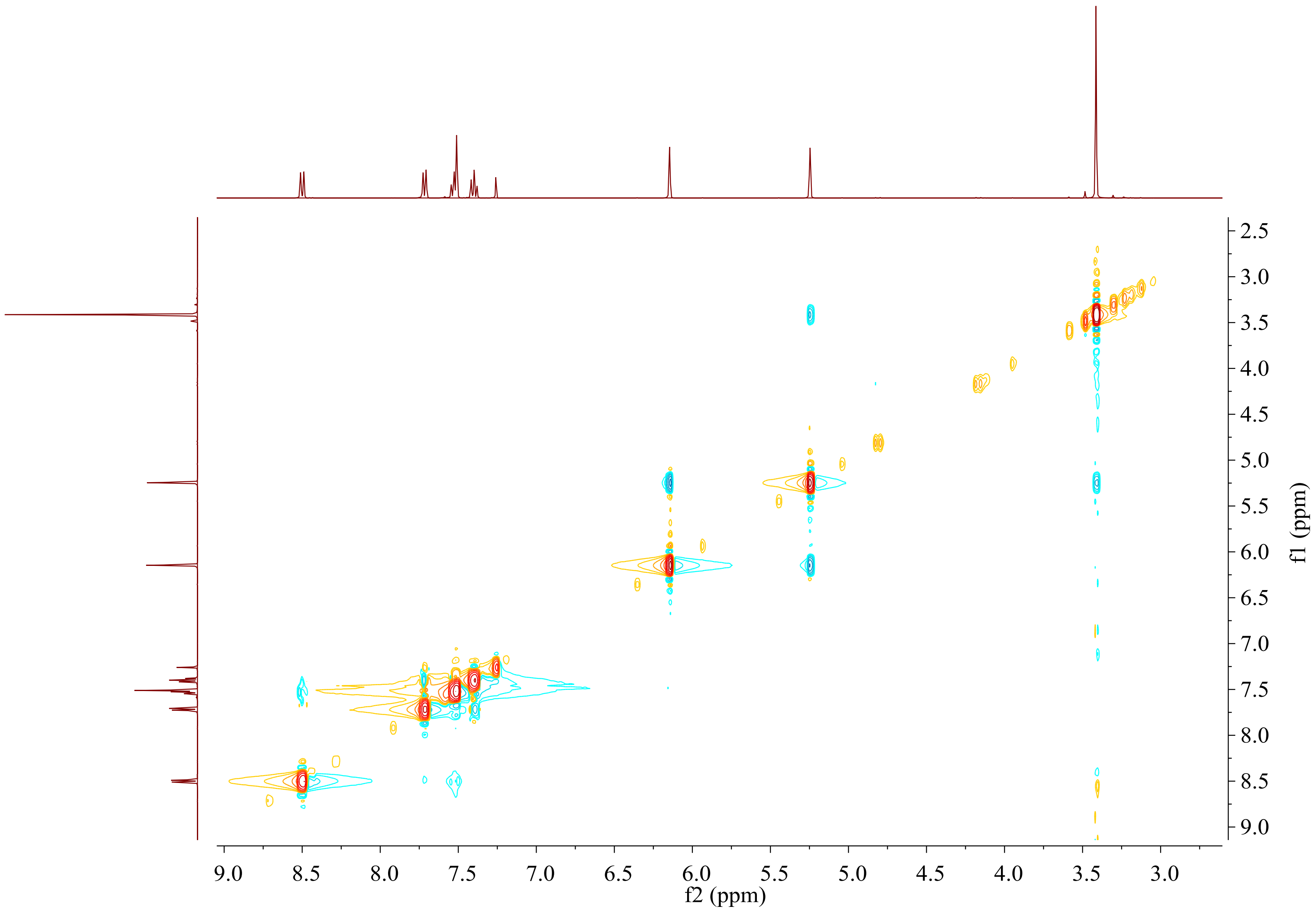

Figure S32. LREIMS of 1,2,3,4-tetrahydro-2-methyl-1,3,4-trioxopyrazino[1,2-a]indole (**5**).

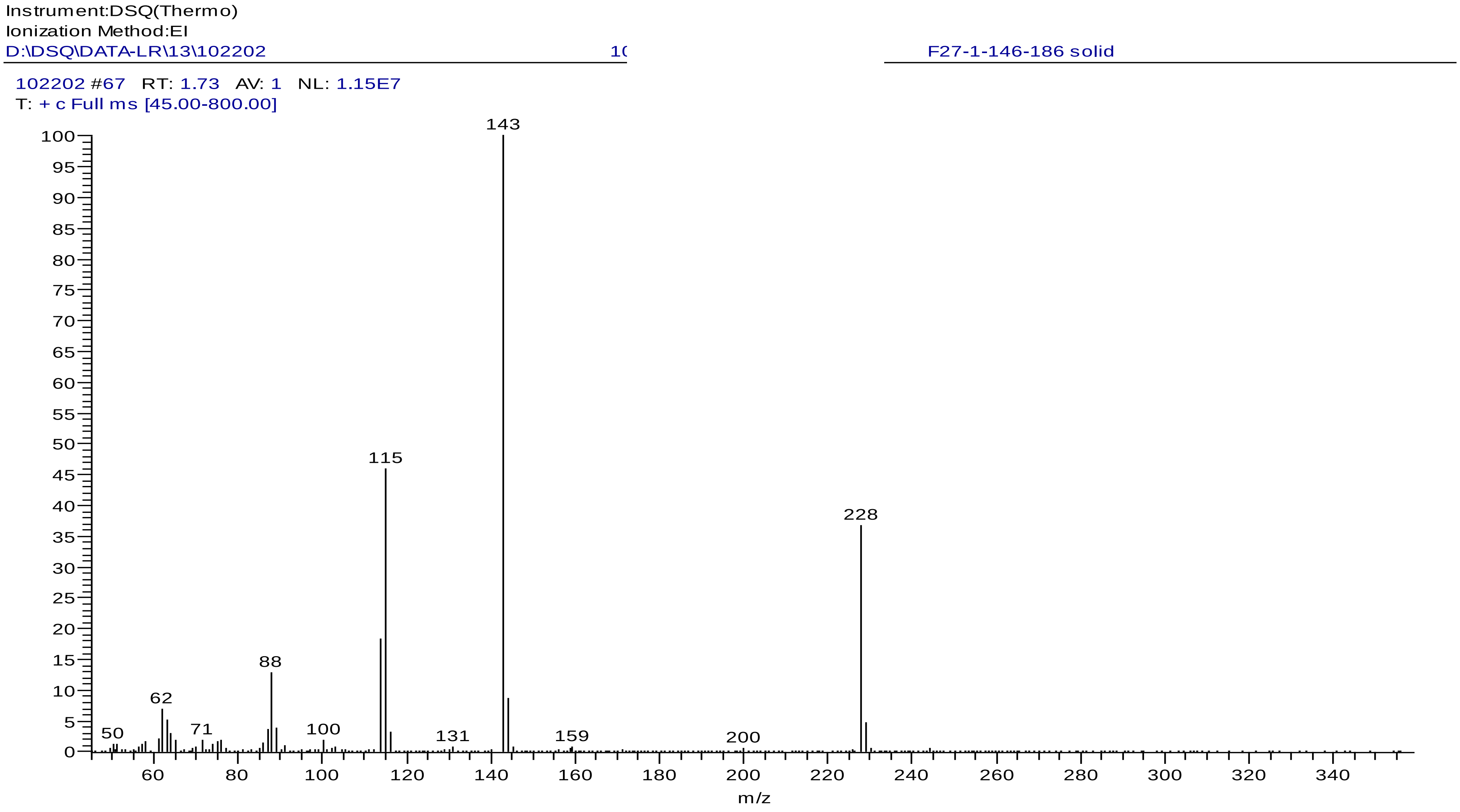

Figure S33. ^{1}H NMR (400 MHz, DMSO-*d*6) spectrum of 1,2,3,4-tetrahydro-2-methyl-1,3,4-trioxopyrazino[1,2-a]indole (**5**).

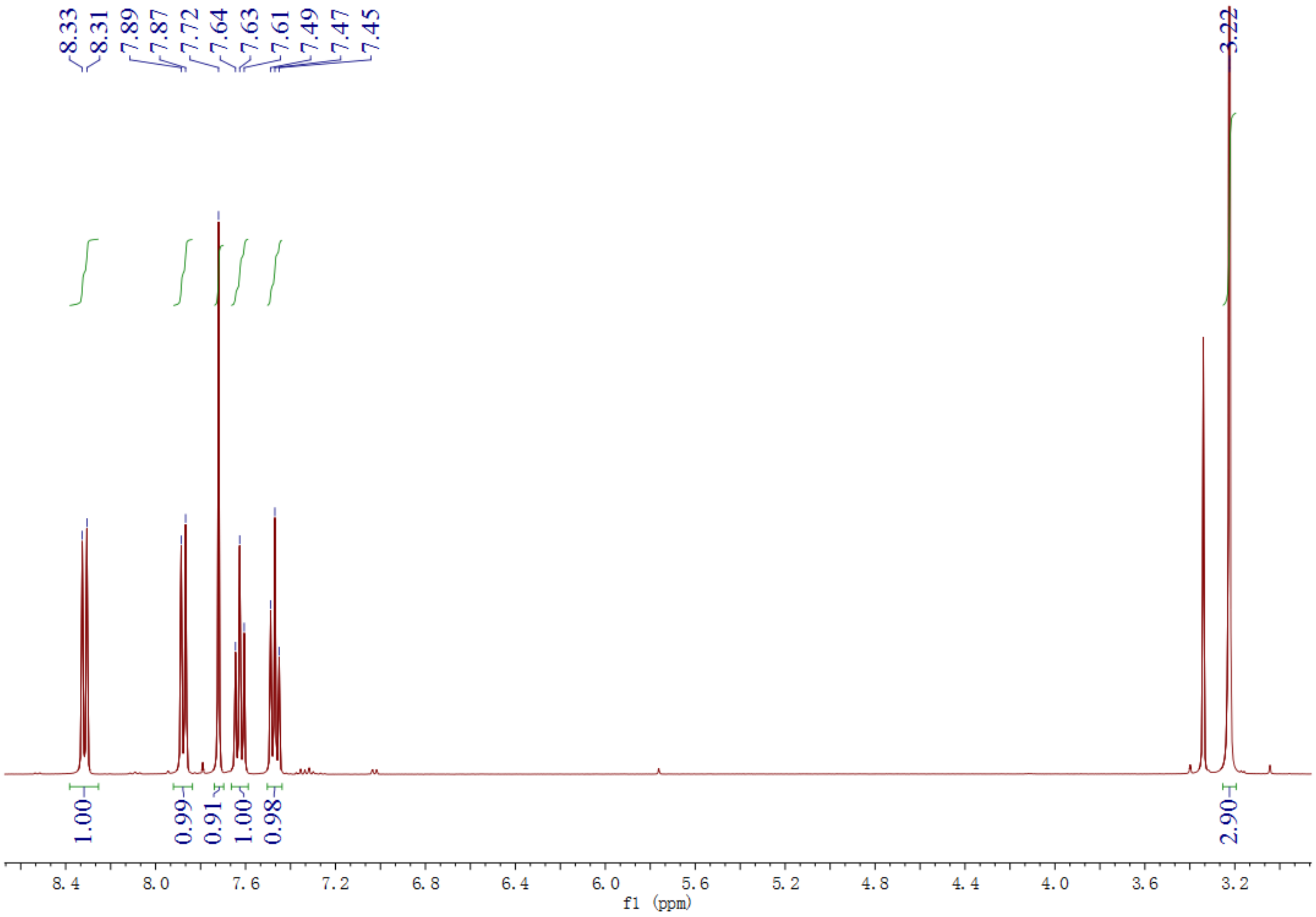

Figure S34. ^{13}C NMR (100 MHz, DMSO-*d*6) spectrum of 1,2,3,4-tetrahydro-2-methyl-1,3,4-trioxopyrazino[1,2-a]indole (**5**).

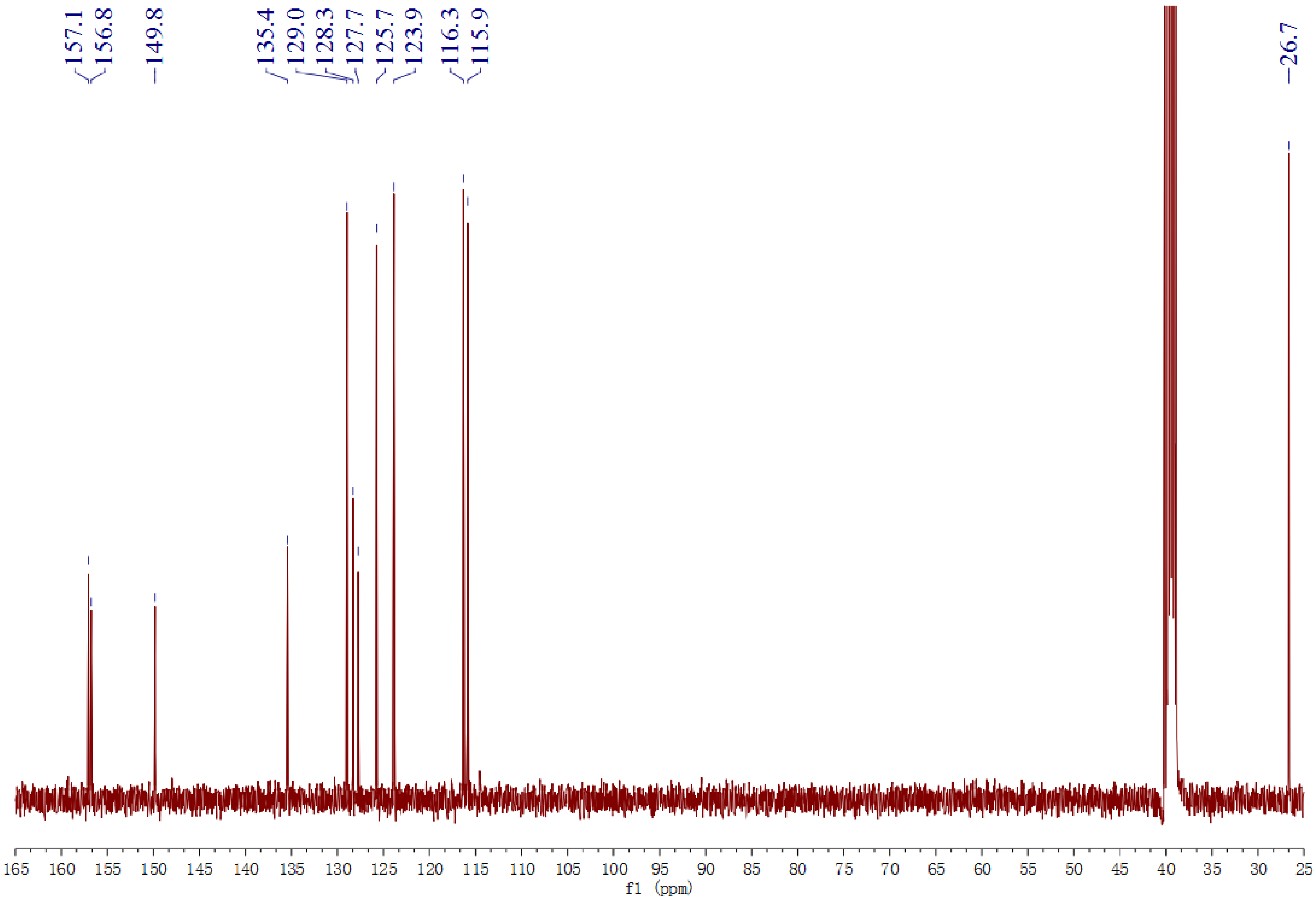

Figure S35. gHMQC spectrum of 1,2,3,4-tetrahydro-2-methyl-1,3,4-trioxopyrazino[1,2-a]indole (**5**).

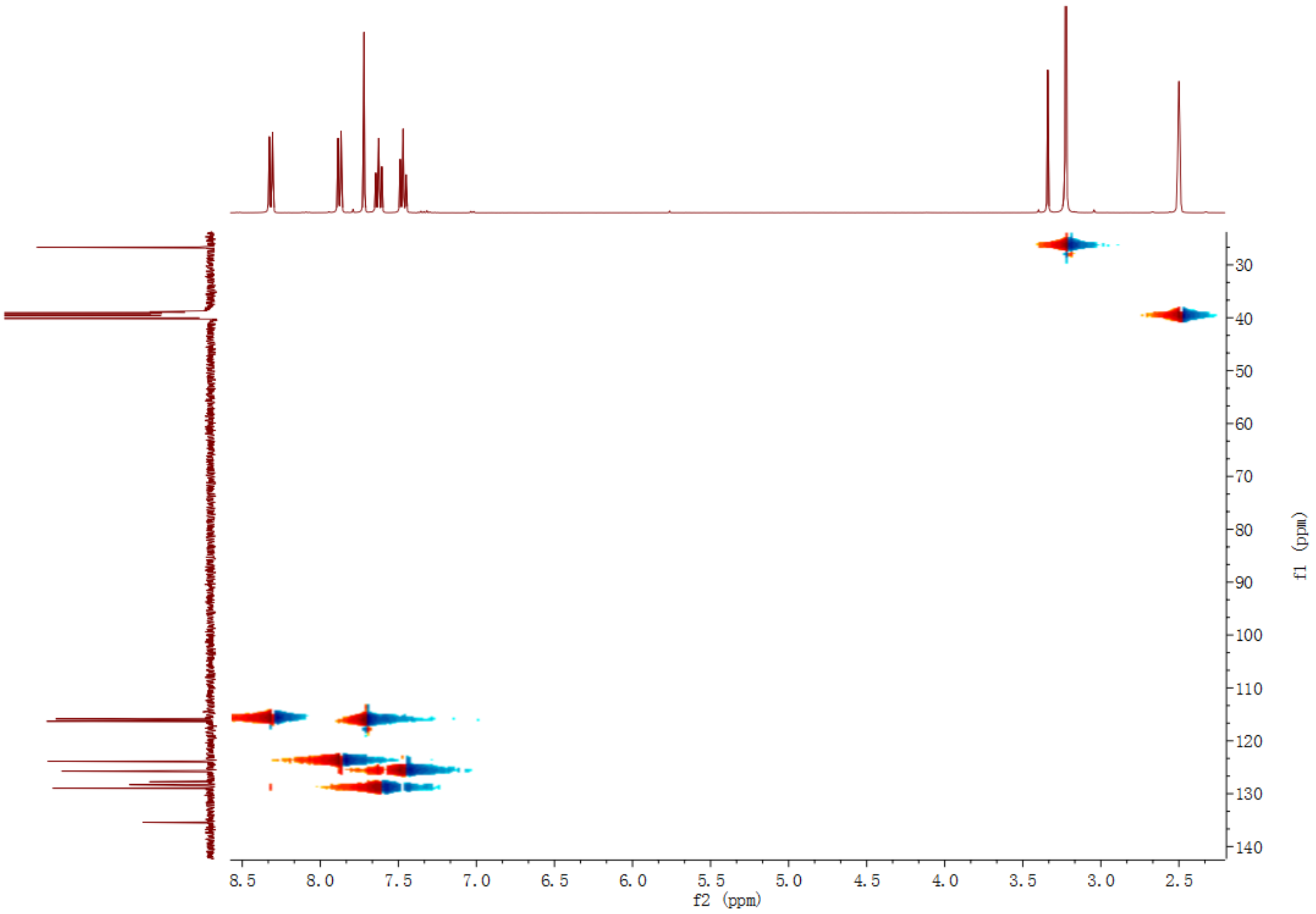

Figure S36. ^{1}H-^{1}H gCOSY spectrum of 1,2,3,4-tetrahydro-2-methyl-1,3,4-trioxopyrazino[1,2-a]indole (**5**).

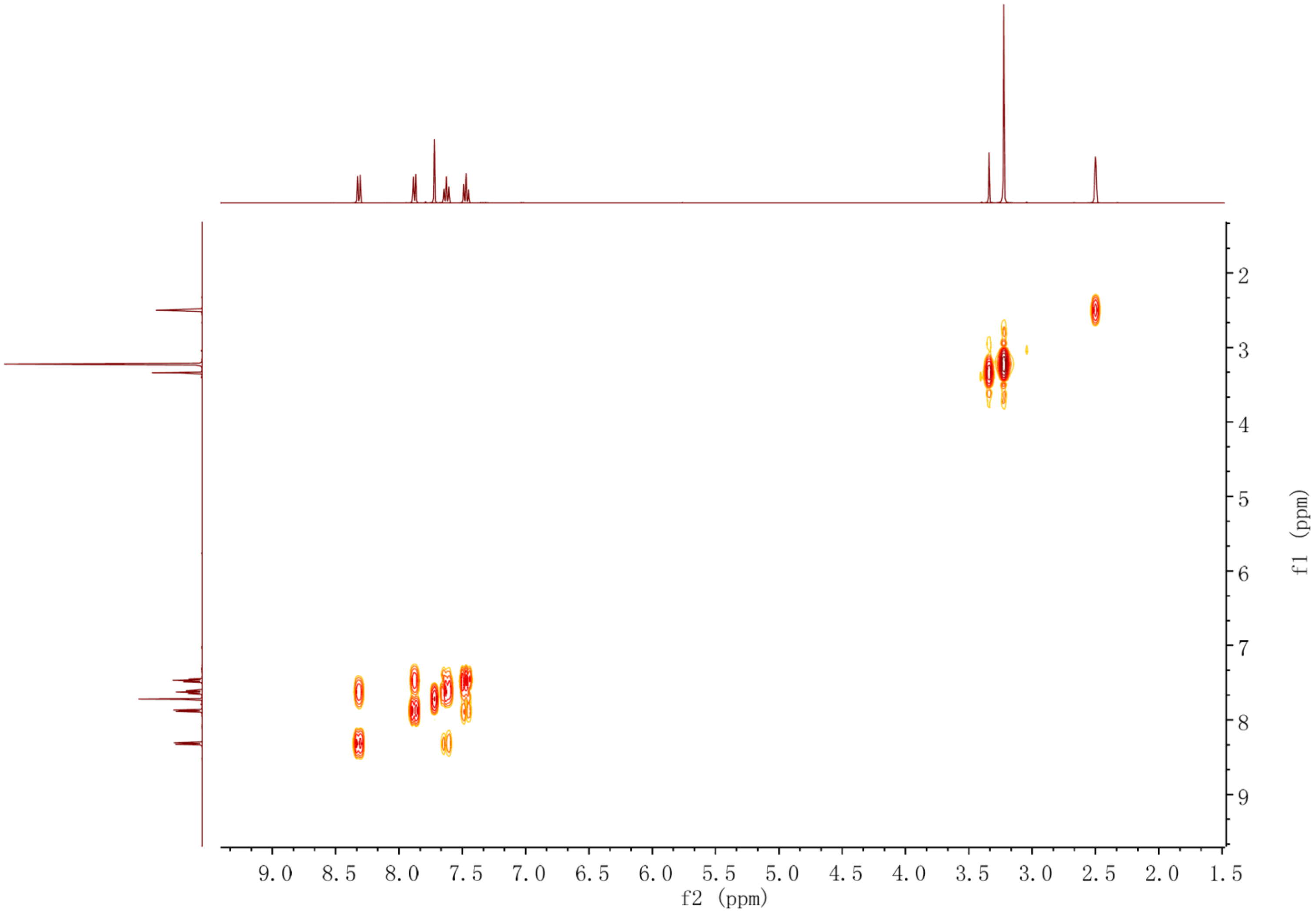

Figure S37. gHMBC spectrum of 1,2,3,4-tetrahydro-2-methyl-1,3,4-trioxopyrazino[1,2-a]indole (**5**).

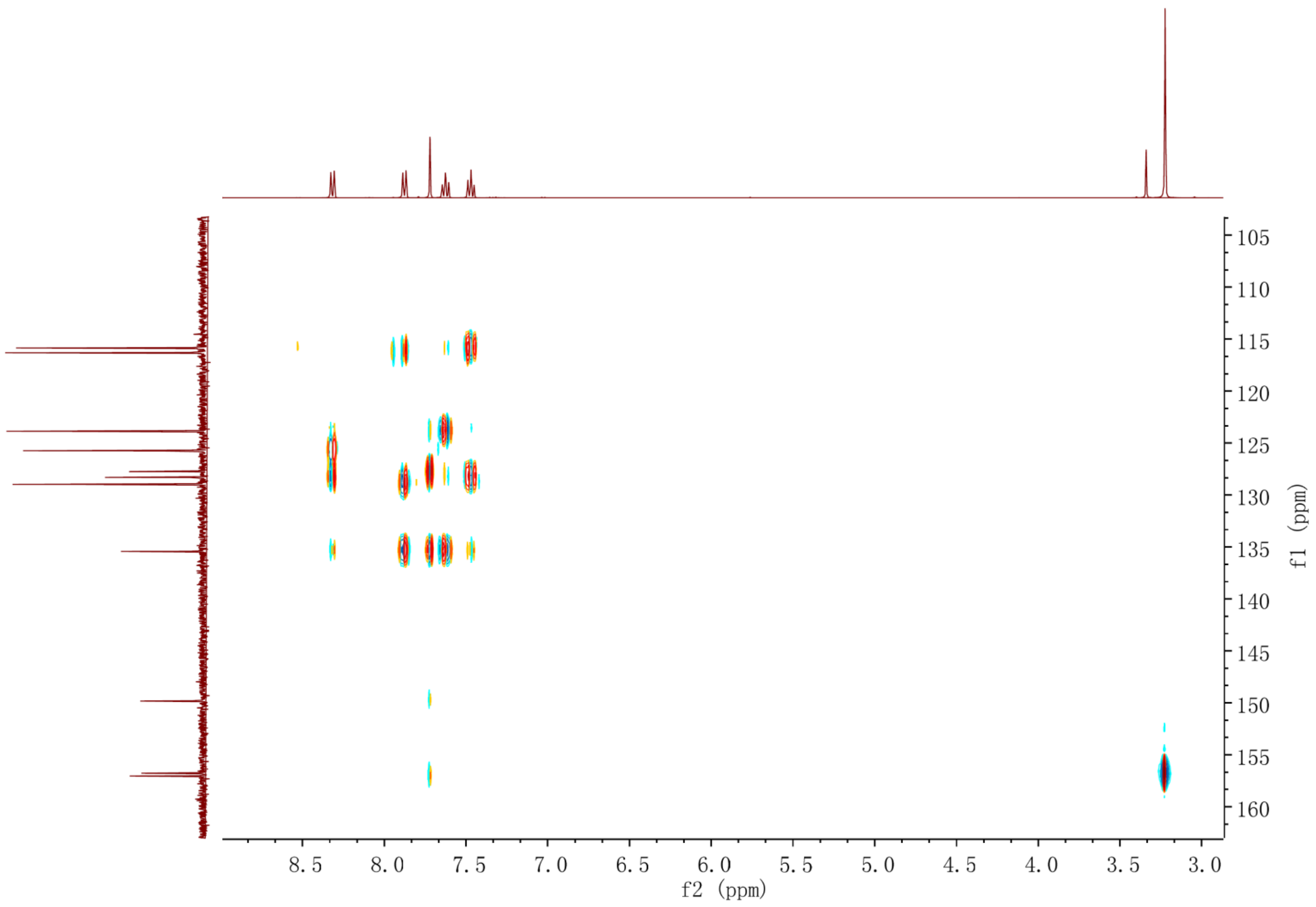

Figure S38. NOESY of 1,2,3,4-tetrahydro-2-methyl-1,3,4-trioxopyrazino[1,2-a]indole (**5**).

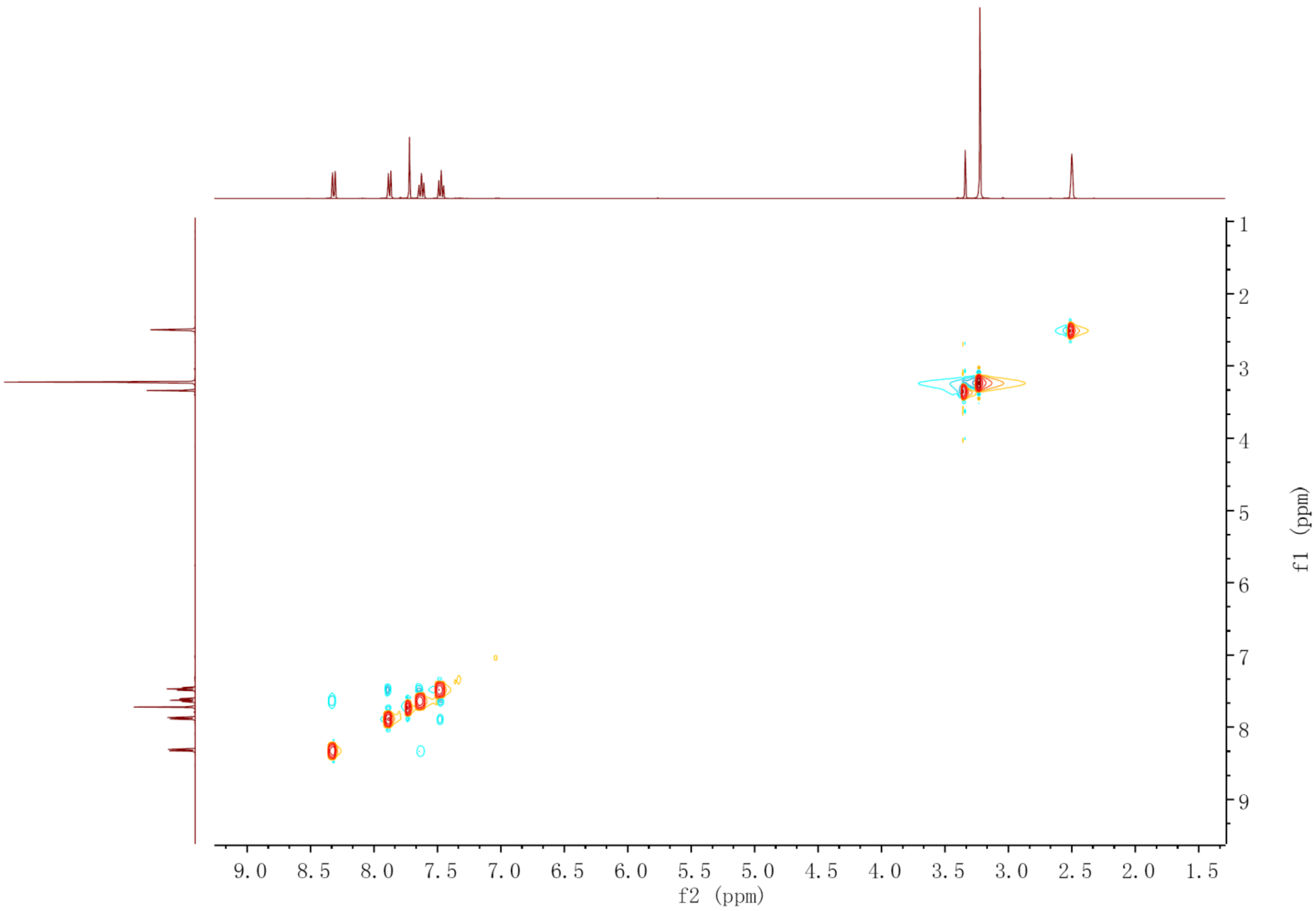

Figure S39. LREIMS of N-methyl-1*H*-indole-2-carboxamide (**6**).

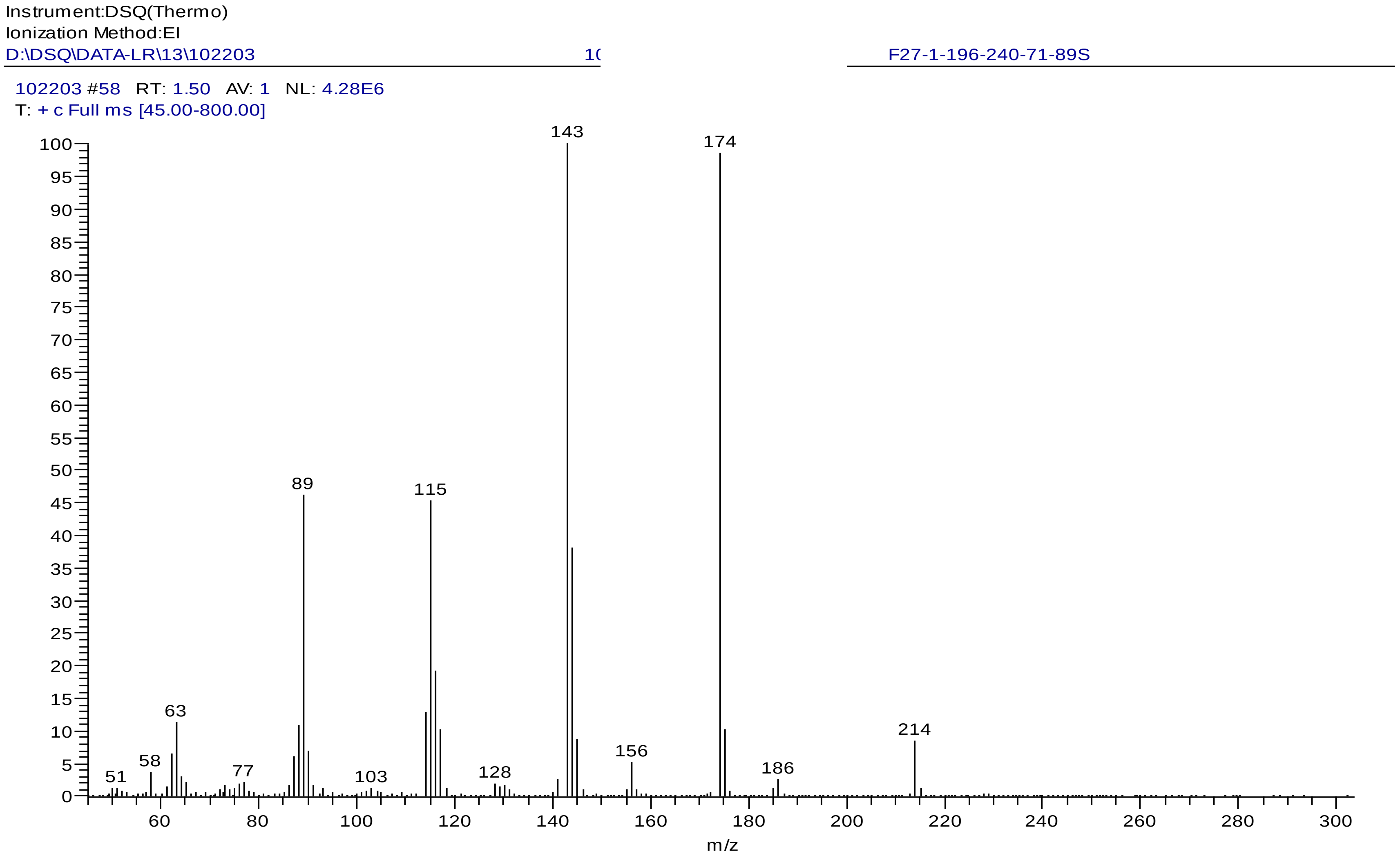

Figure S40. ^{1}H NMR (400 MHz, acetone-*d*6) spectrum of N-methyl-1*H*-indole-2-carboxamide (**6**).

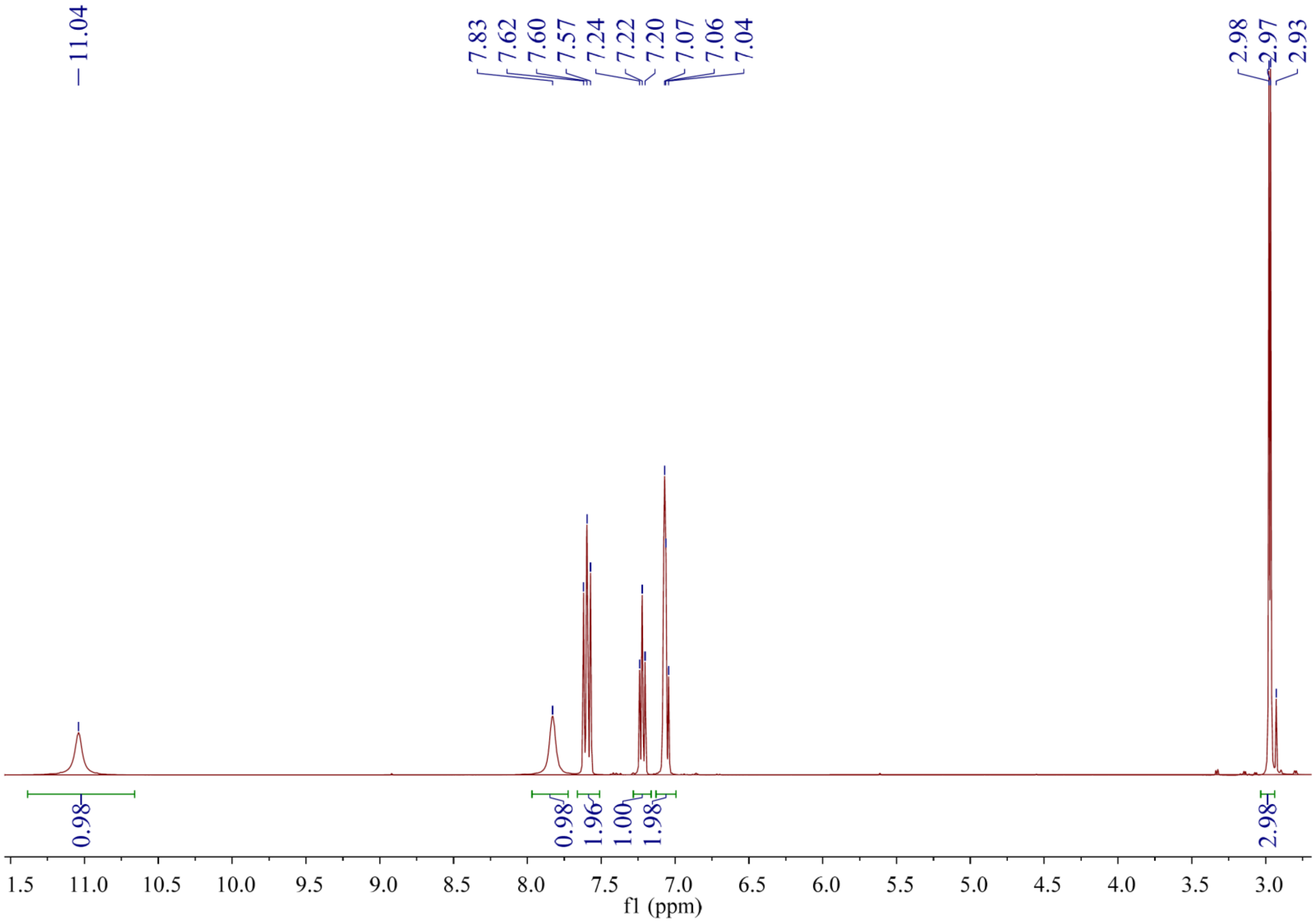

Figure S41. ^{13}C NMR (100 MHz, acetone-*d*6) spectrum of N-methyl-1*H*-indole-2-carboxamide (**6**).

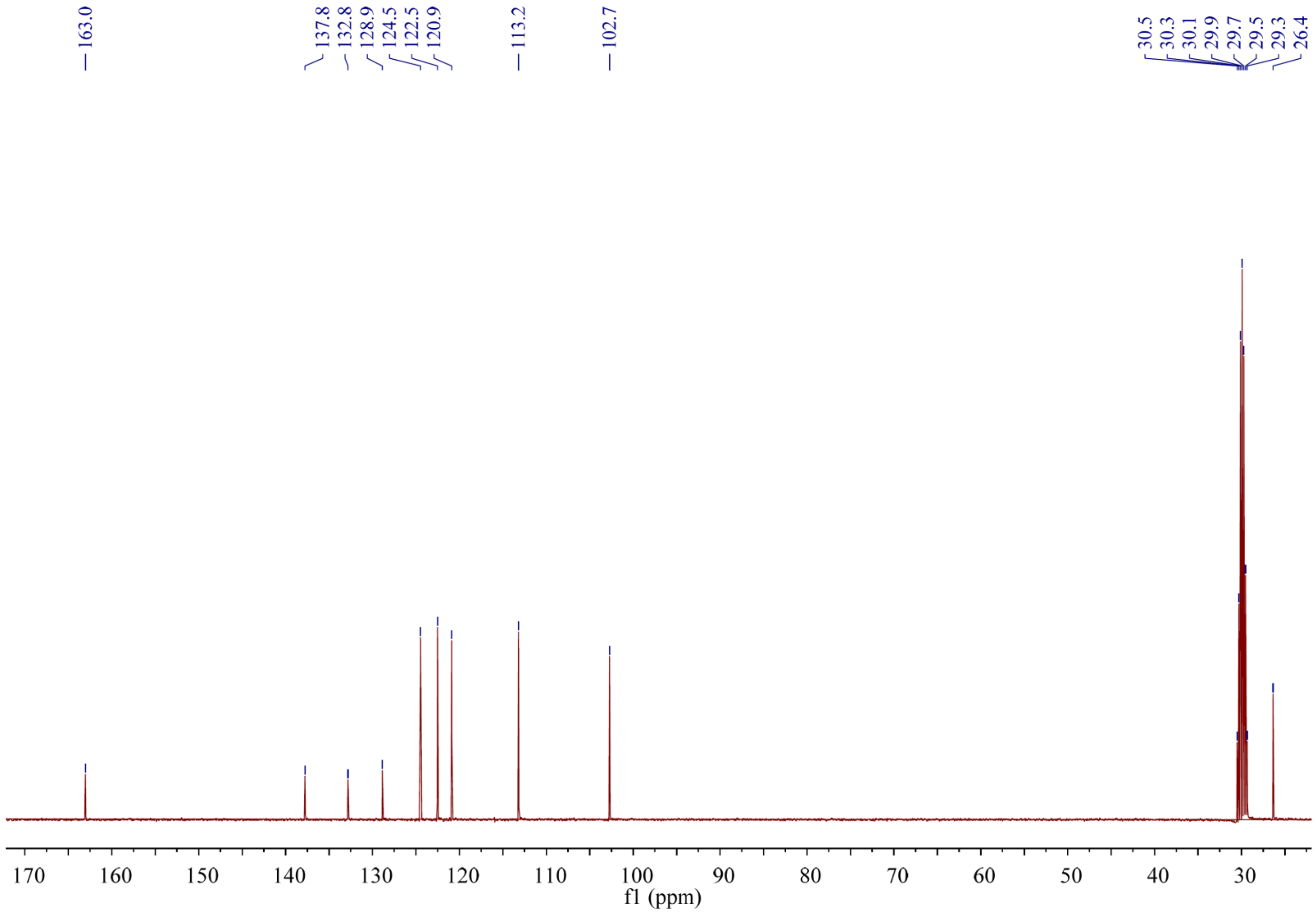

Figure S42. gHMQC spectrum of N- methyl-1*H*-indole-2-carboxamide (**6**).

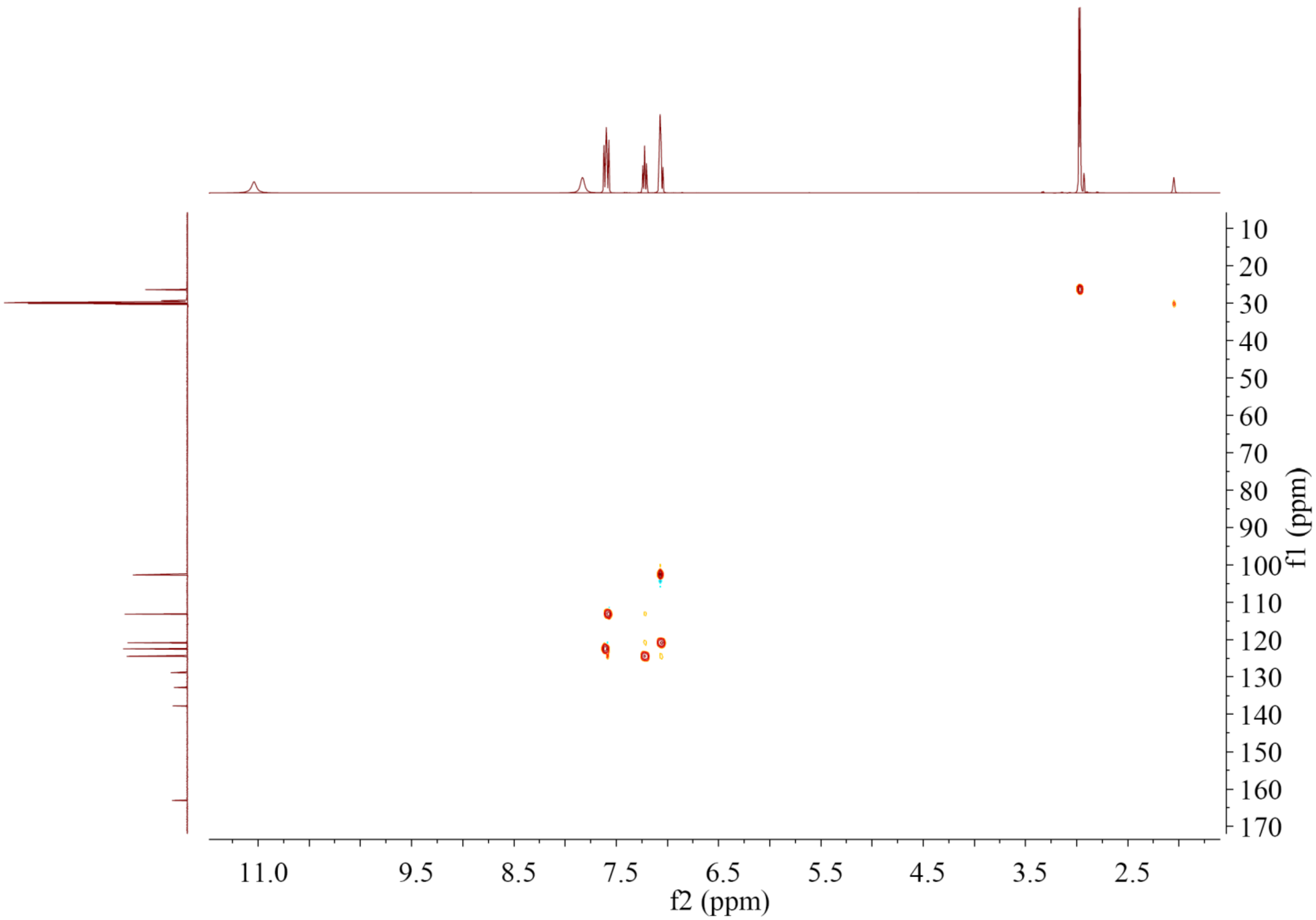

Figure S43. ^{1}H-^{1}H gCOSY spectrum of N-methyl-1*H*-indole-2-carboxamide (**6**).

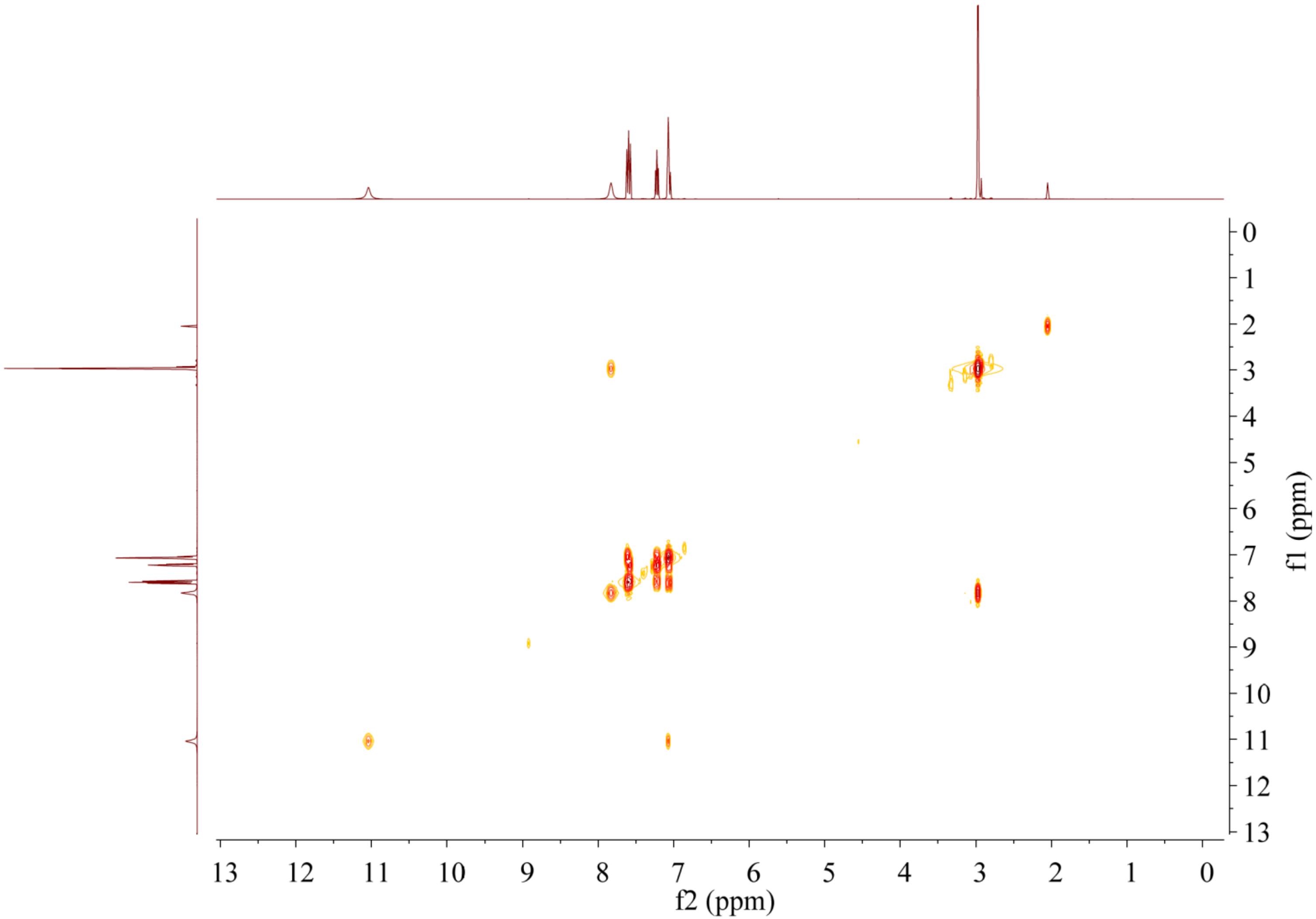

Figure S44. gHMBC spectrum of N-methyl-1*H*-indole-2-carboxamide (**6**).

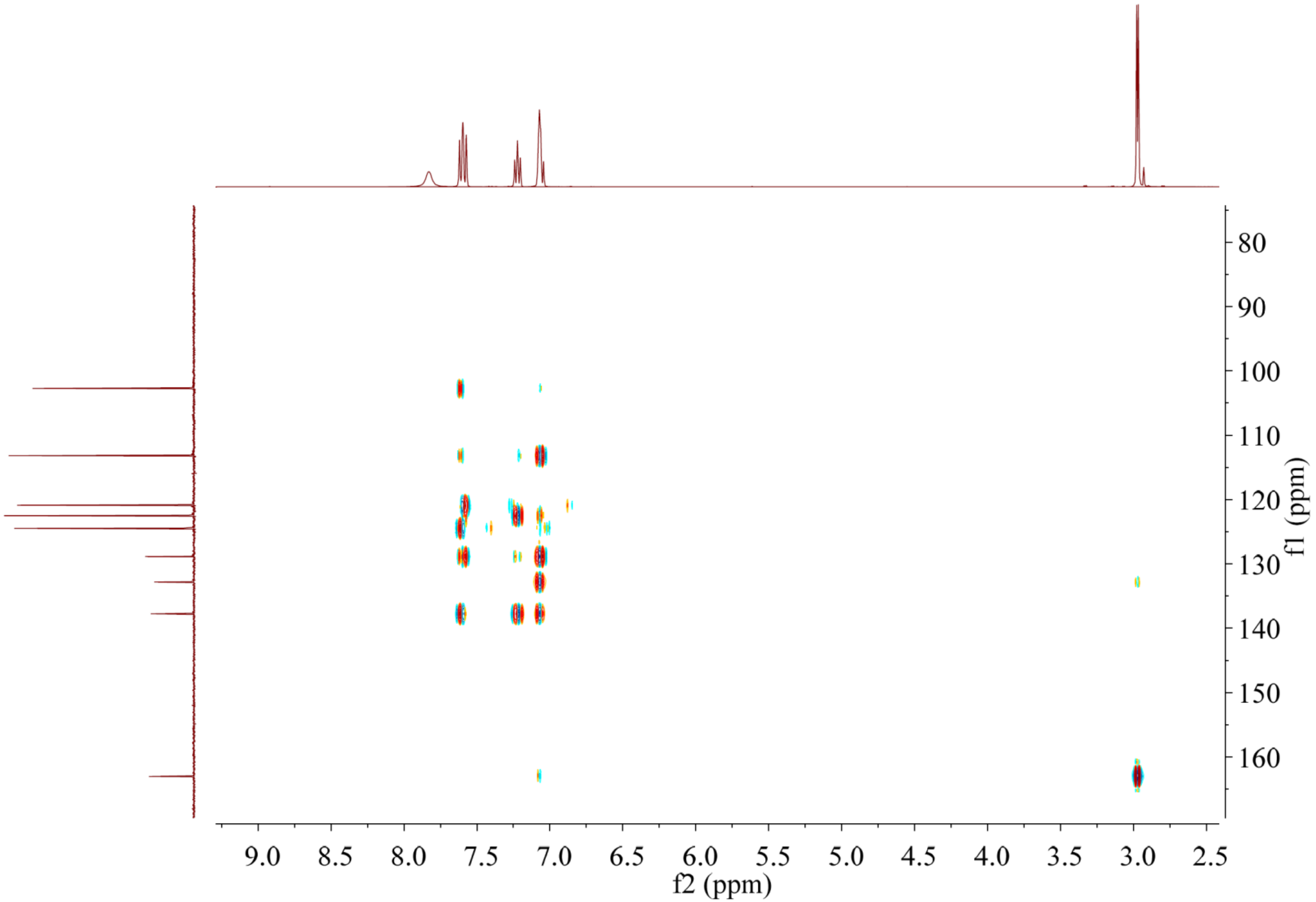

Figure S45. NOESY of N-methyl-1*H*-indole-2-carboxamide (**6**).

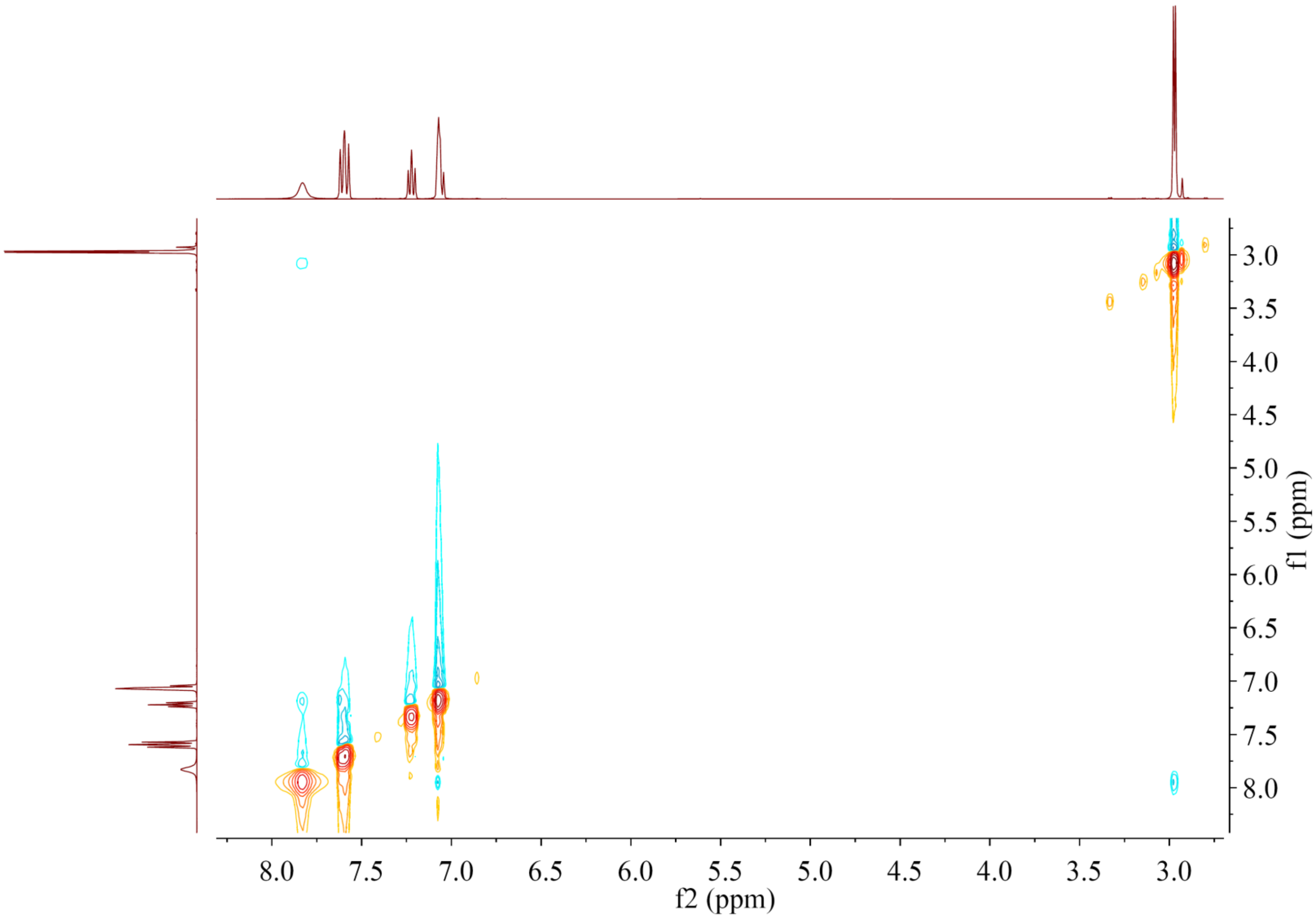

Figure S46. LREIMS of gliotoxin (**7**).

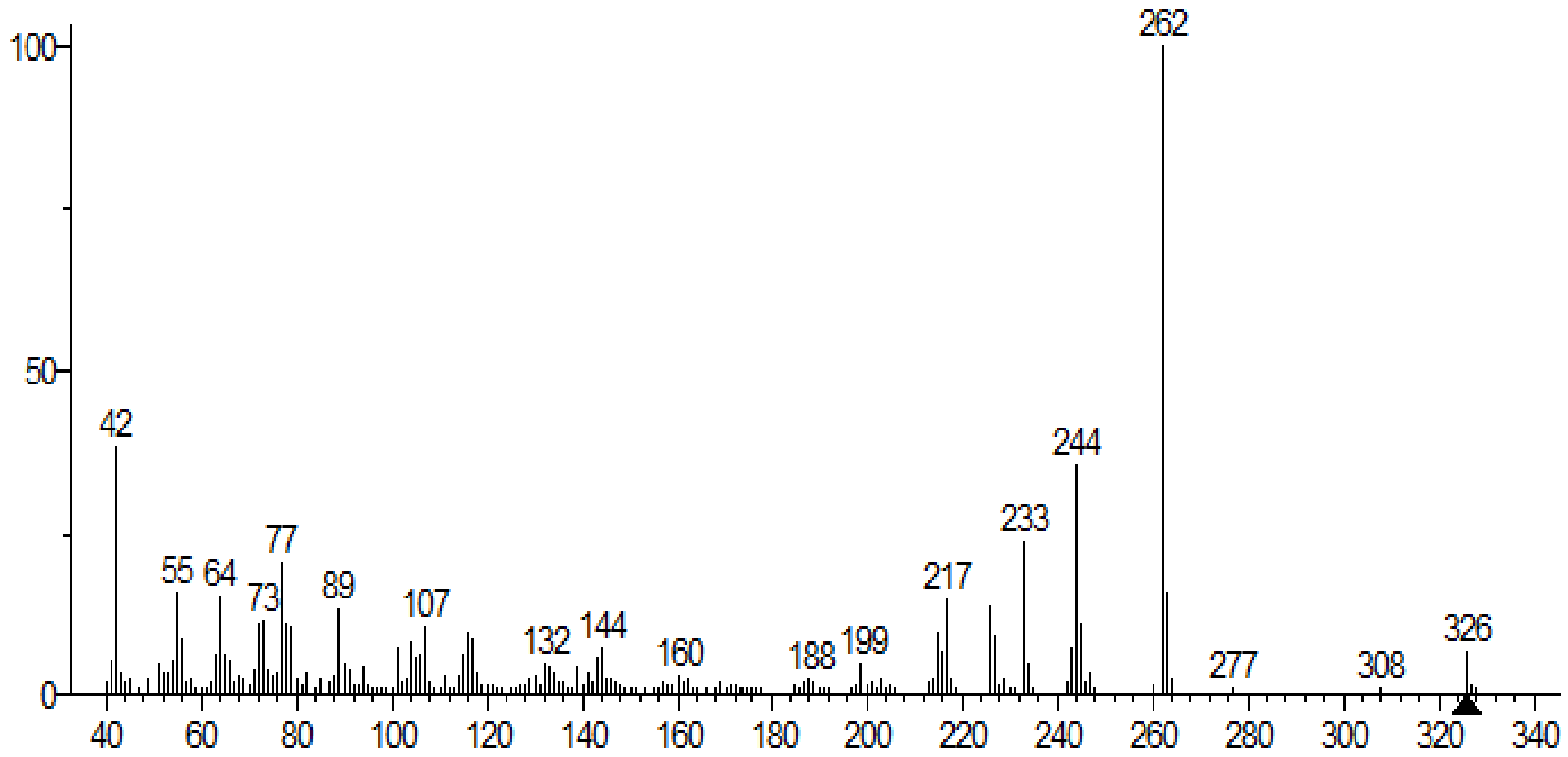

Figure S47. ^{1}H NMR (300 MHz, $CDCl_3$) spectrum of gliotoxin (**7**).

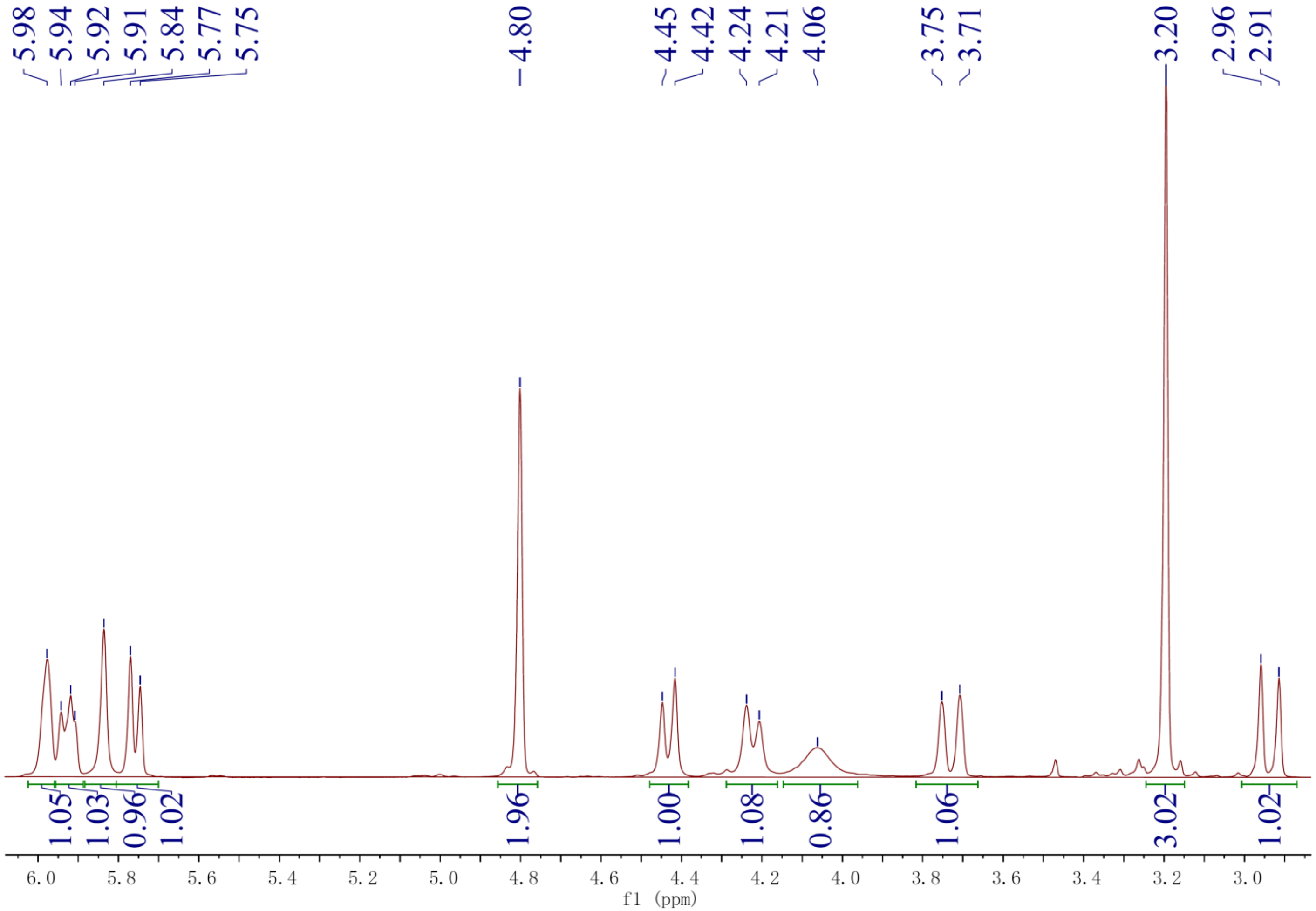

Figure S48. ^{13}C NMR (75 MHz, $CDCl_3$) spectrum of gliotoxin (**7**).

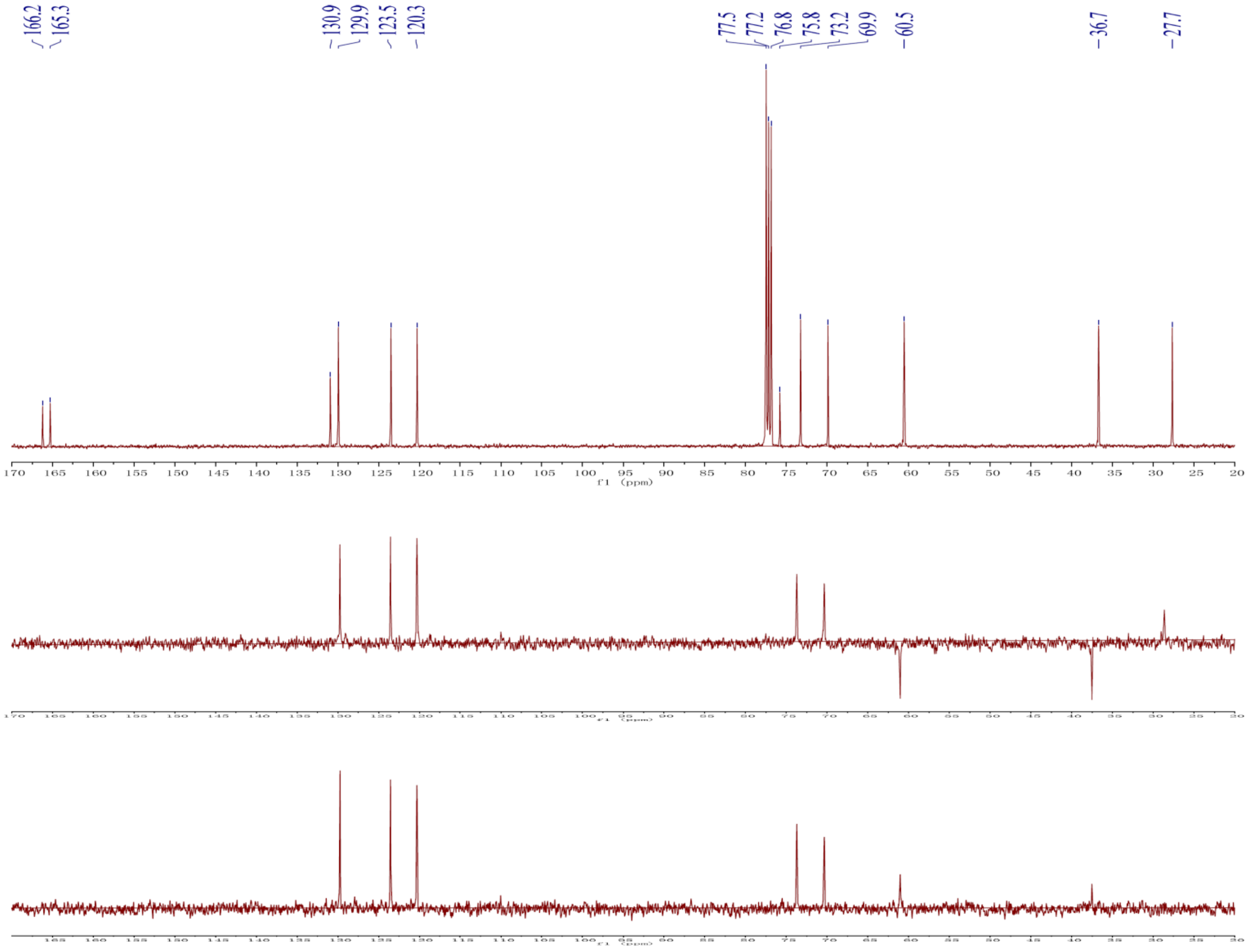

Figure S49. ^{1}H NMR (300 MHz, $CDCl_3$) spectrum of acetylgliotoxin (**8**).

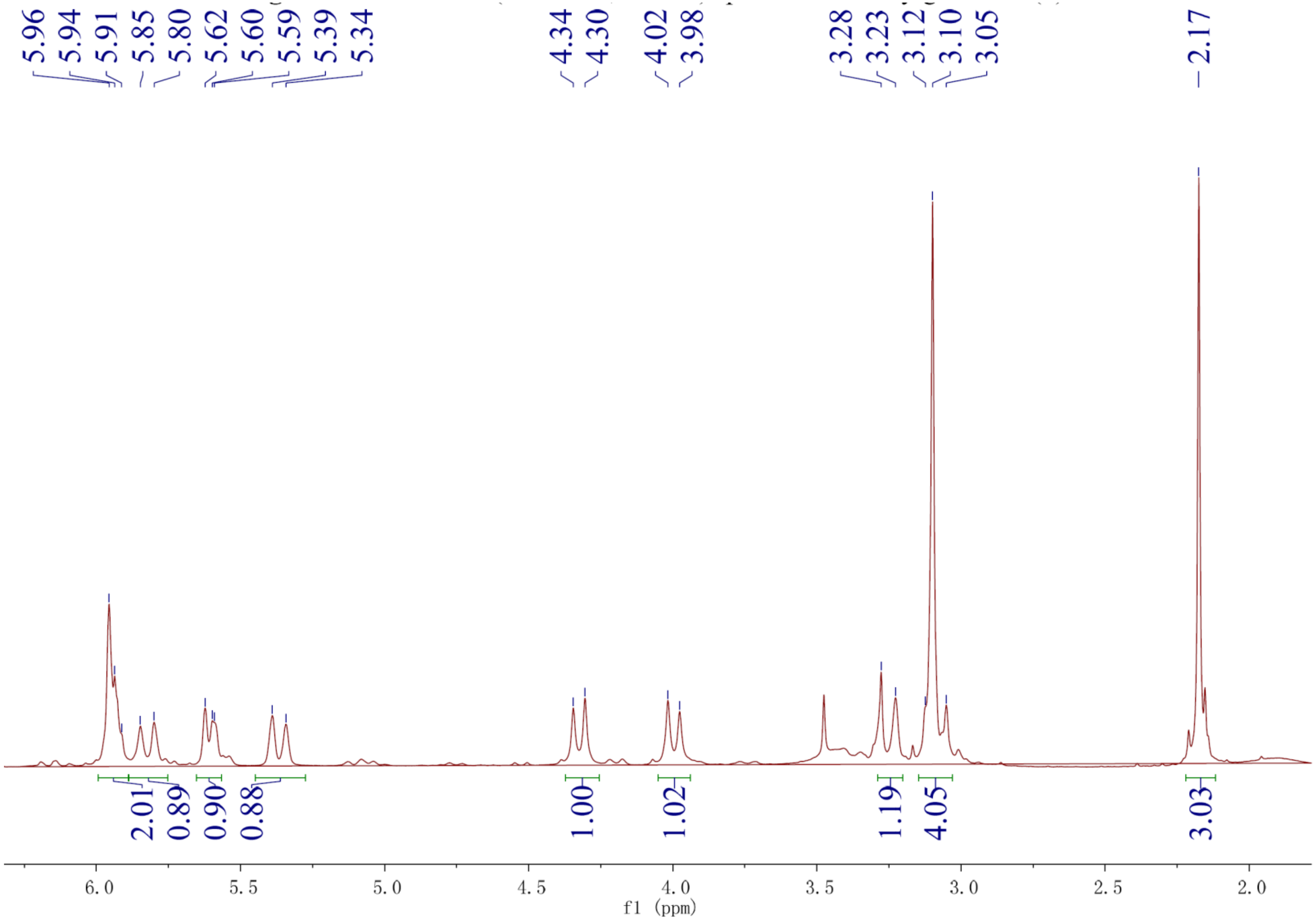

Figure S50.^{13}C NMR (75 MHz, CDCl3) spectrum of acetylgliotoxin (**8**).

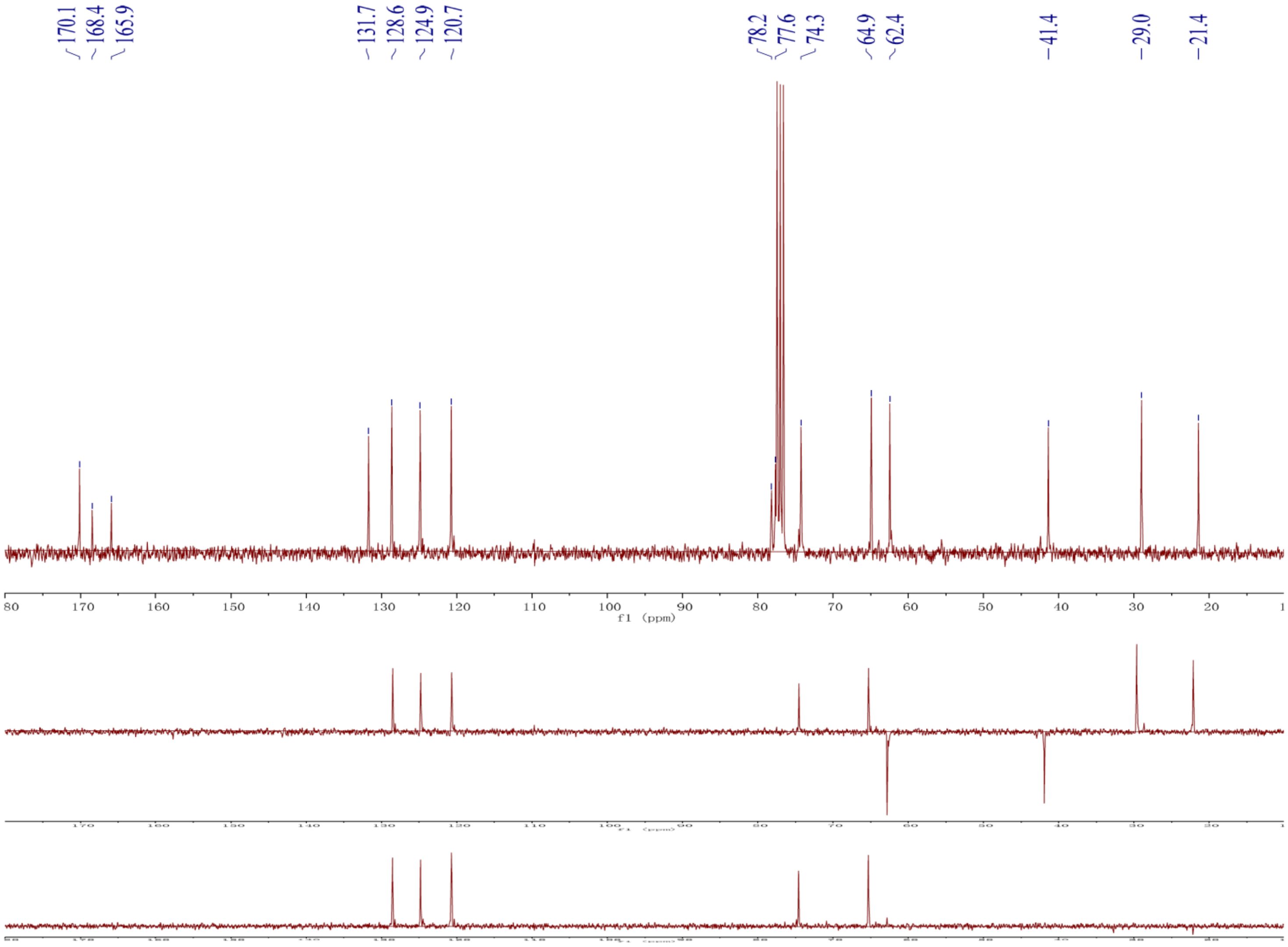

Figure S51. ^{1}H NMR (400 MHz, $CDCl_3$) spectrum of reduced gliotoxin (**9**).

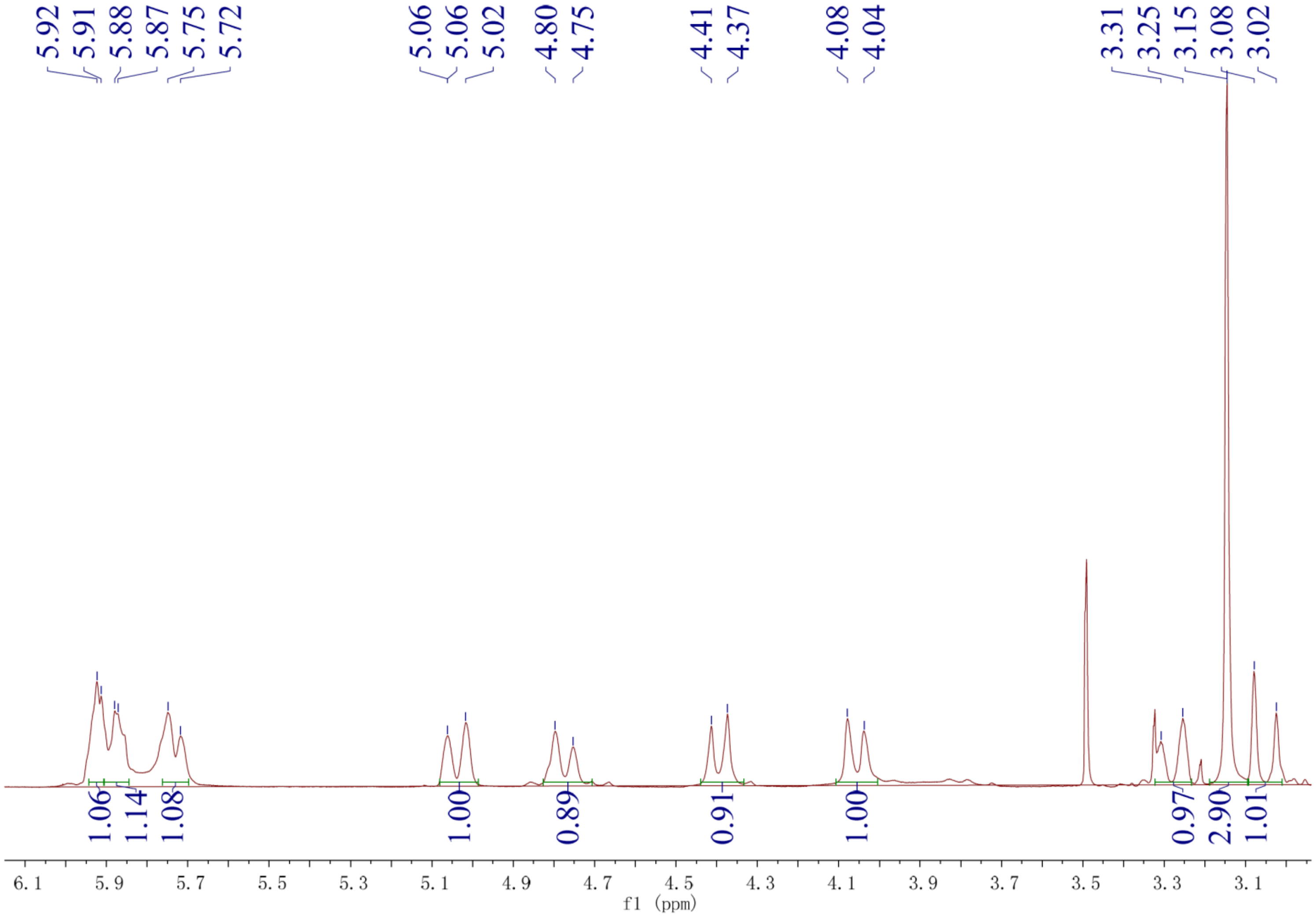

Figure S52. ^{13}C NMR (100 MHz, $CDCl_3$) spectrum of reduced gliotoxin (**9**).

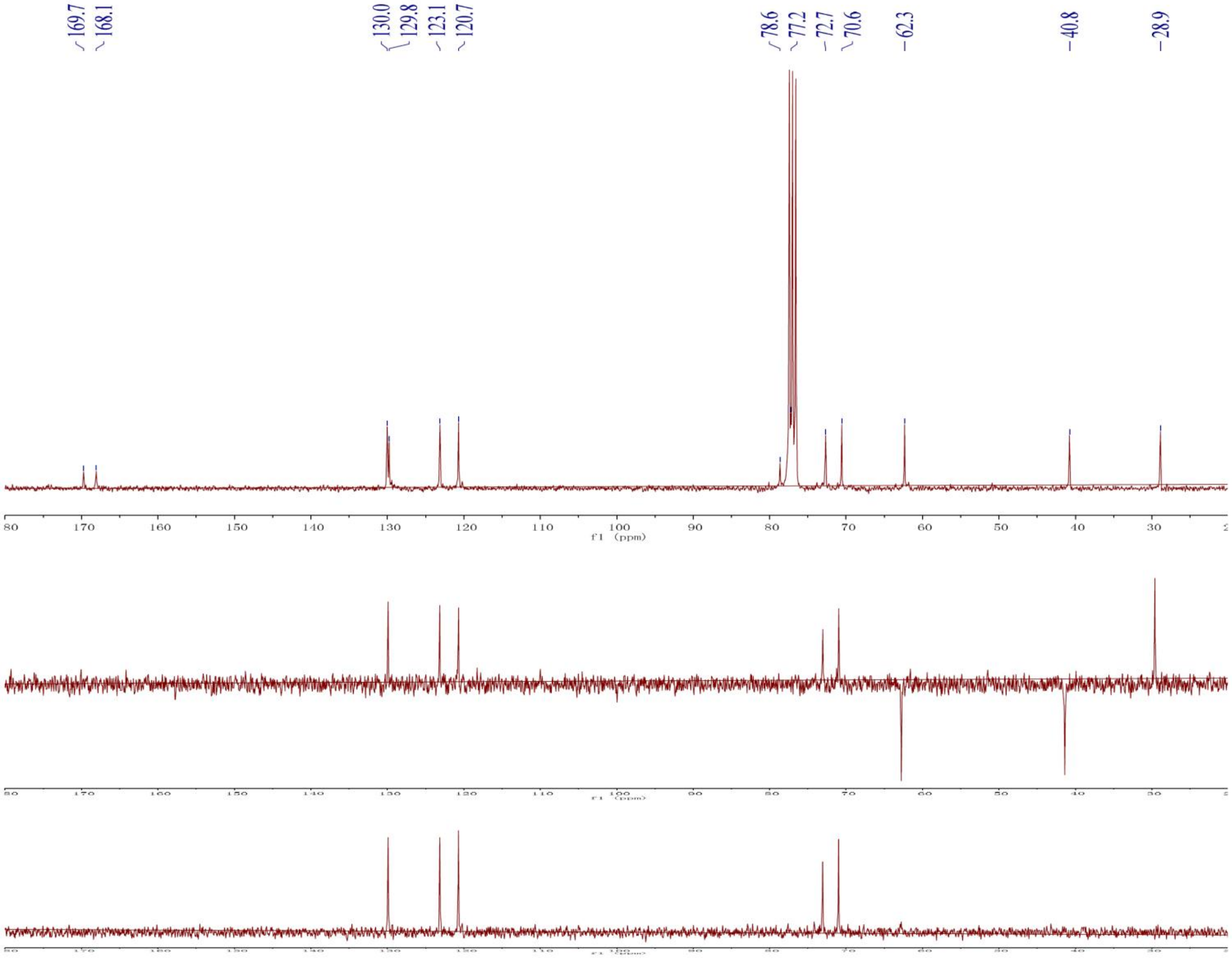

Figure S53. ^{1}H NMR (400 MHz, $CDCl_3$) spectrum of 6-acetylbis(methylthio)gliotoxin (**10**).

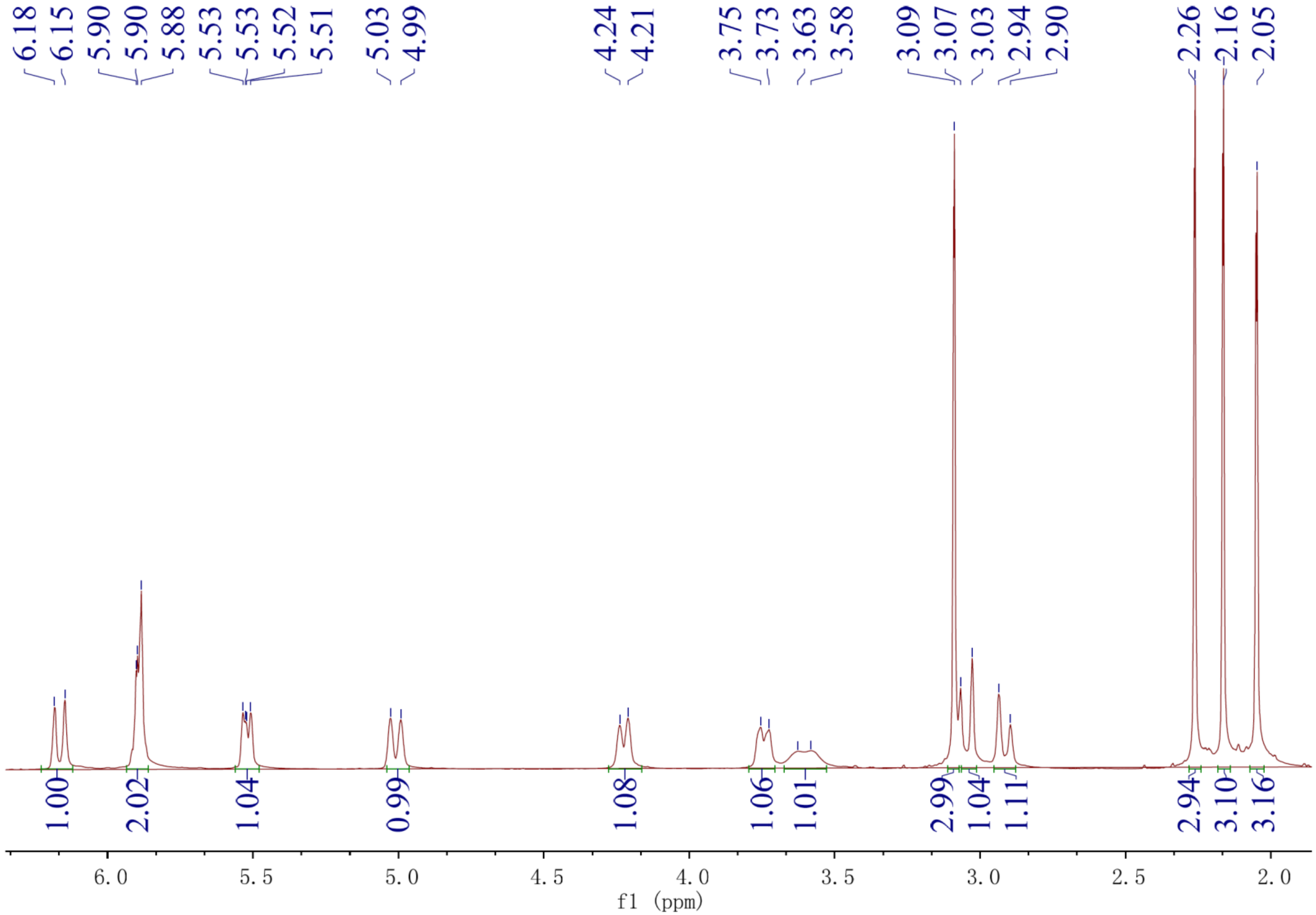

Figure S54. ^{13}C NMR (100 MHz, $CDCl_3$) spectrum of 6-acetylbis(methylthio)gliotoxin (**10**).

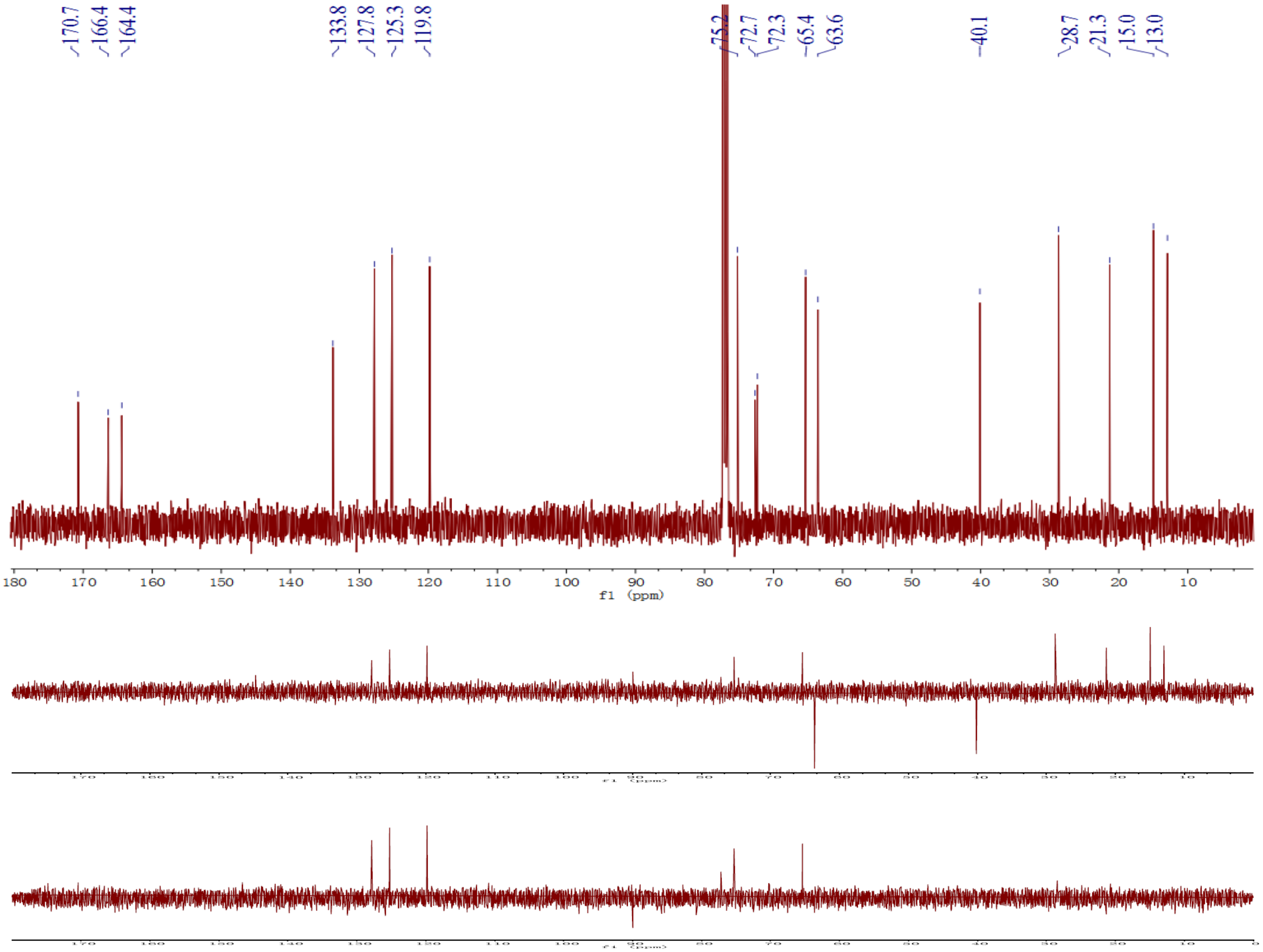

Figure S55. ^{1}H NMR (300 MHz, $CDCl_3$) spectrum of bisdethiobis(methylthio)gliotoxin (**11**).

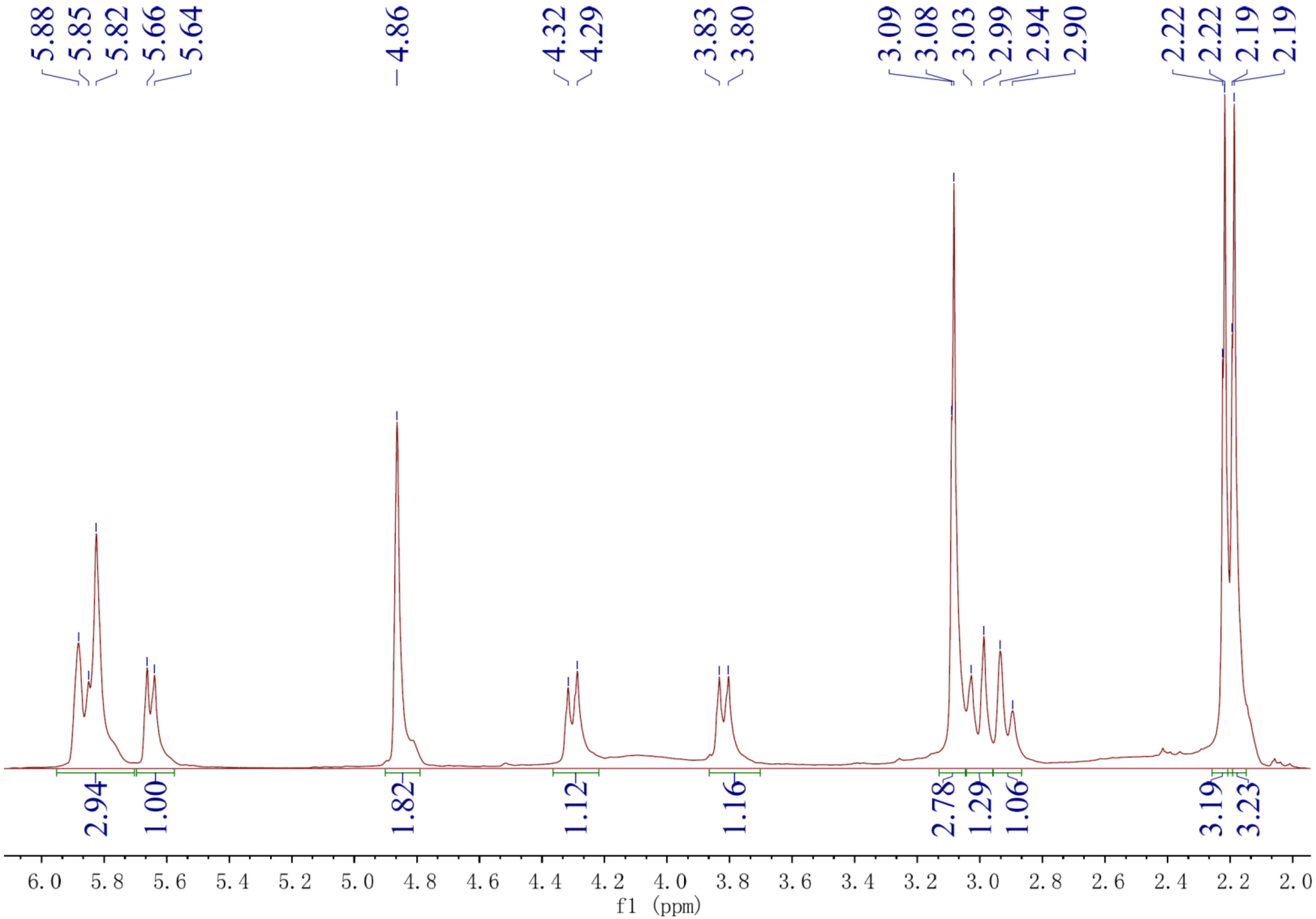

Figure S56. ^{13}C NMR (100 MHz, $CDCl_3$) spectrum of bisdethiobis(methylthio)gliotoxin (**11**).

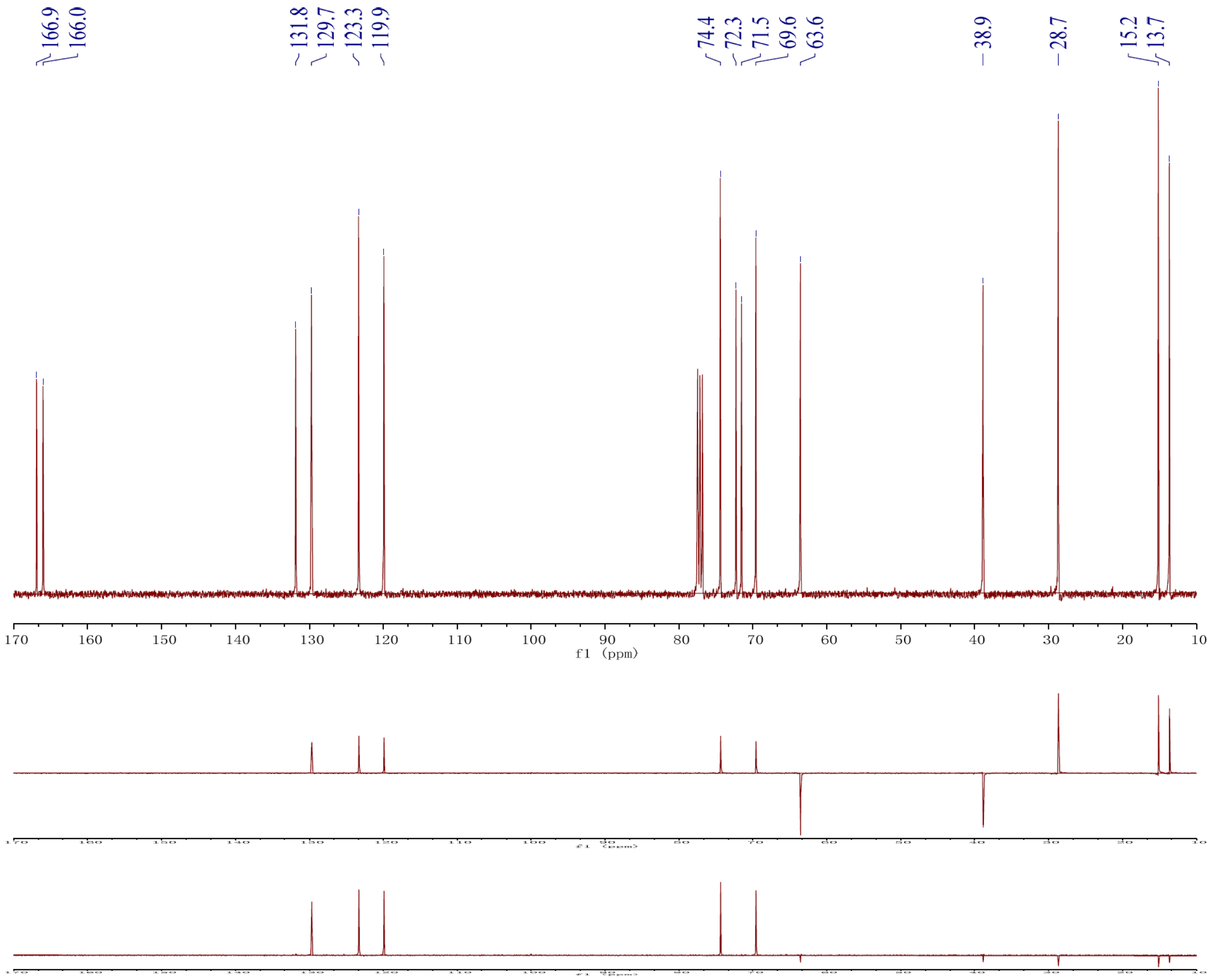

Figure S57. ^{1}H NMR (400 MHz, $CDCl_3$) spectrum of didehydrobisdethiobis(methylthio)gliotoxin (**12**).

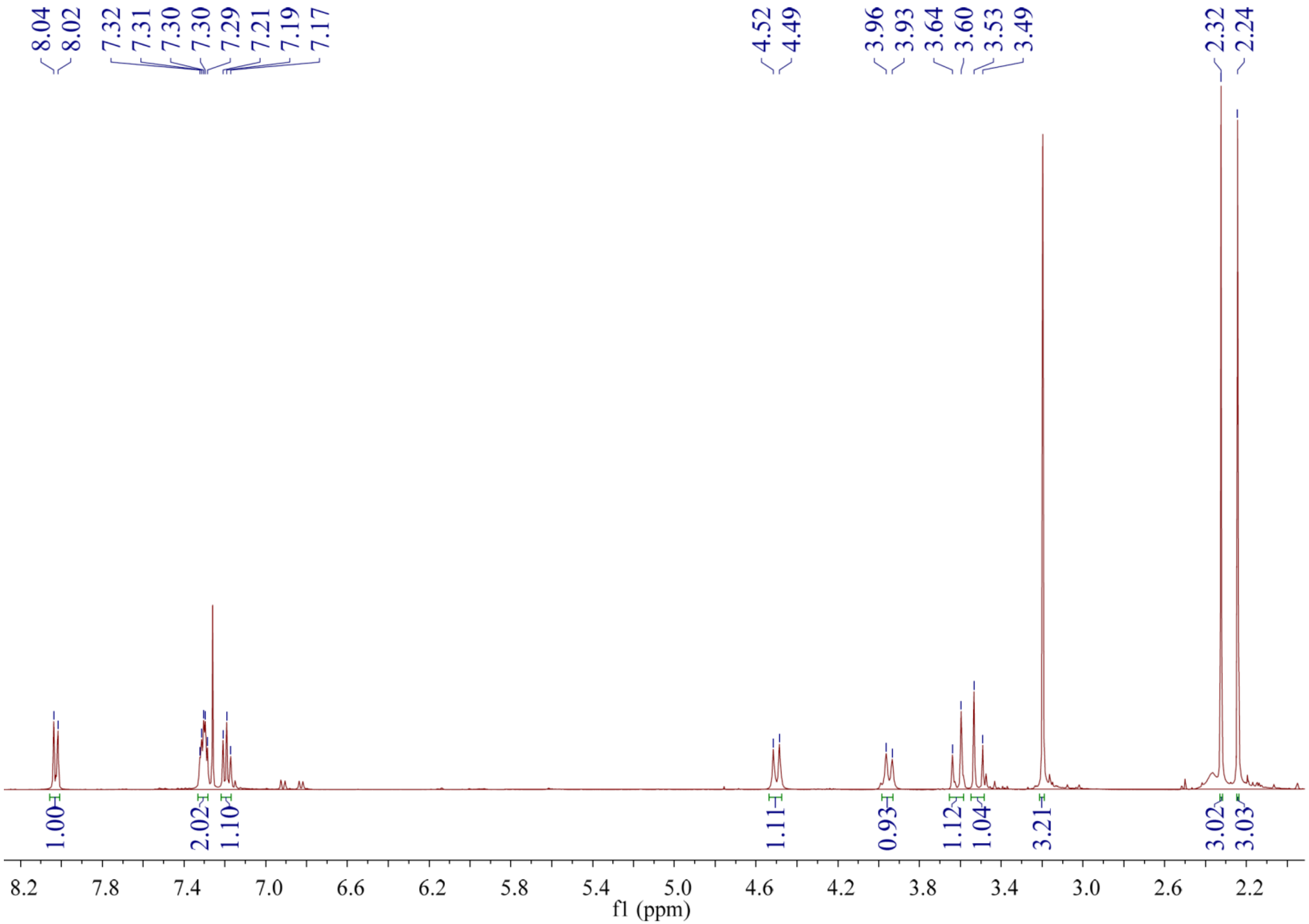

Figure S58. ^{13}C NMR (100 MHz, $CDCl_3$) spectrum of didehydrobisdethiobis(methylthio)gliotoxin (**12**).

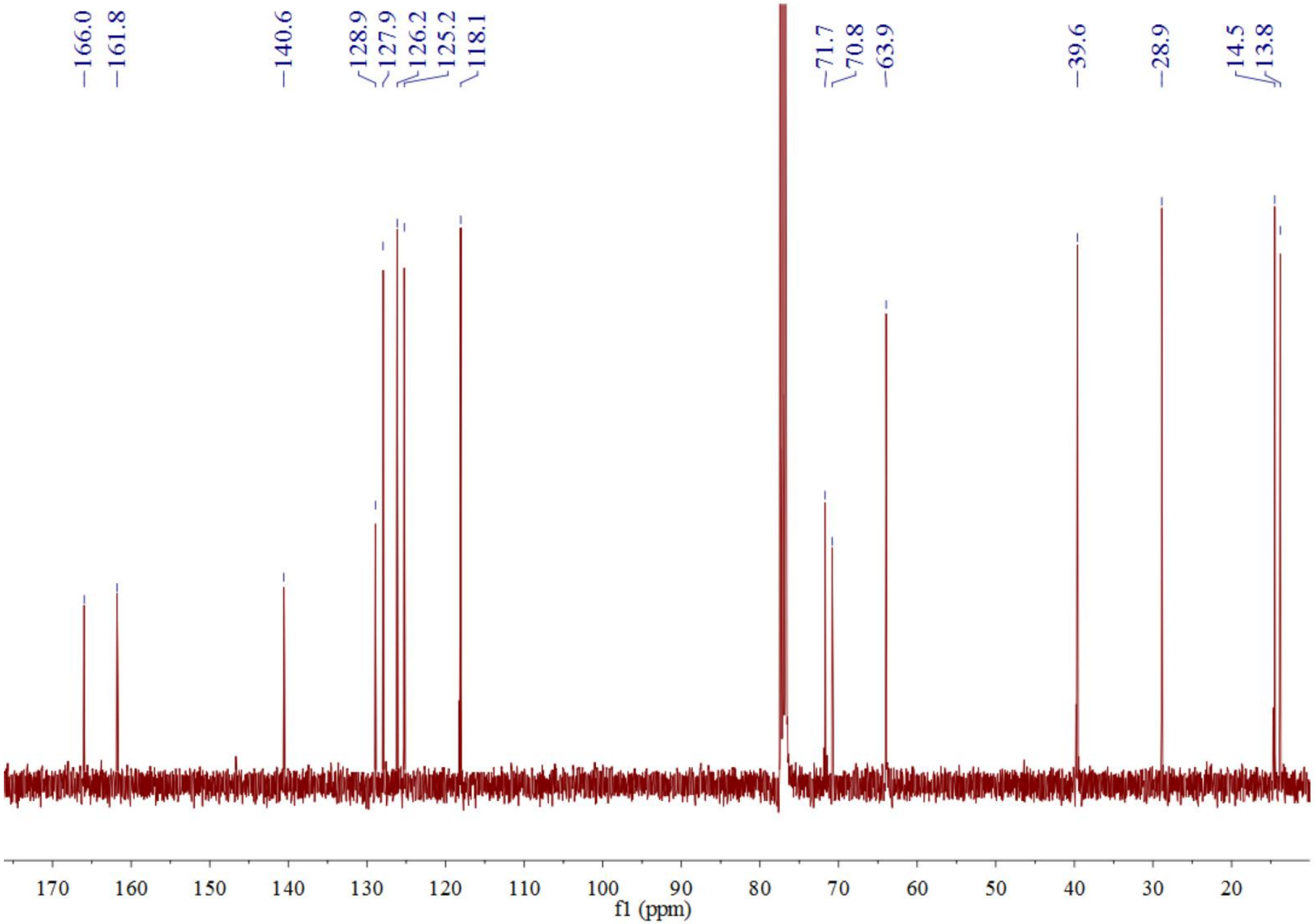

Figure S59. ^{1}H NMR (300 MHz, DMSO-*d*6) spectrum of bis-N-norgliovictin (**13**).

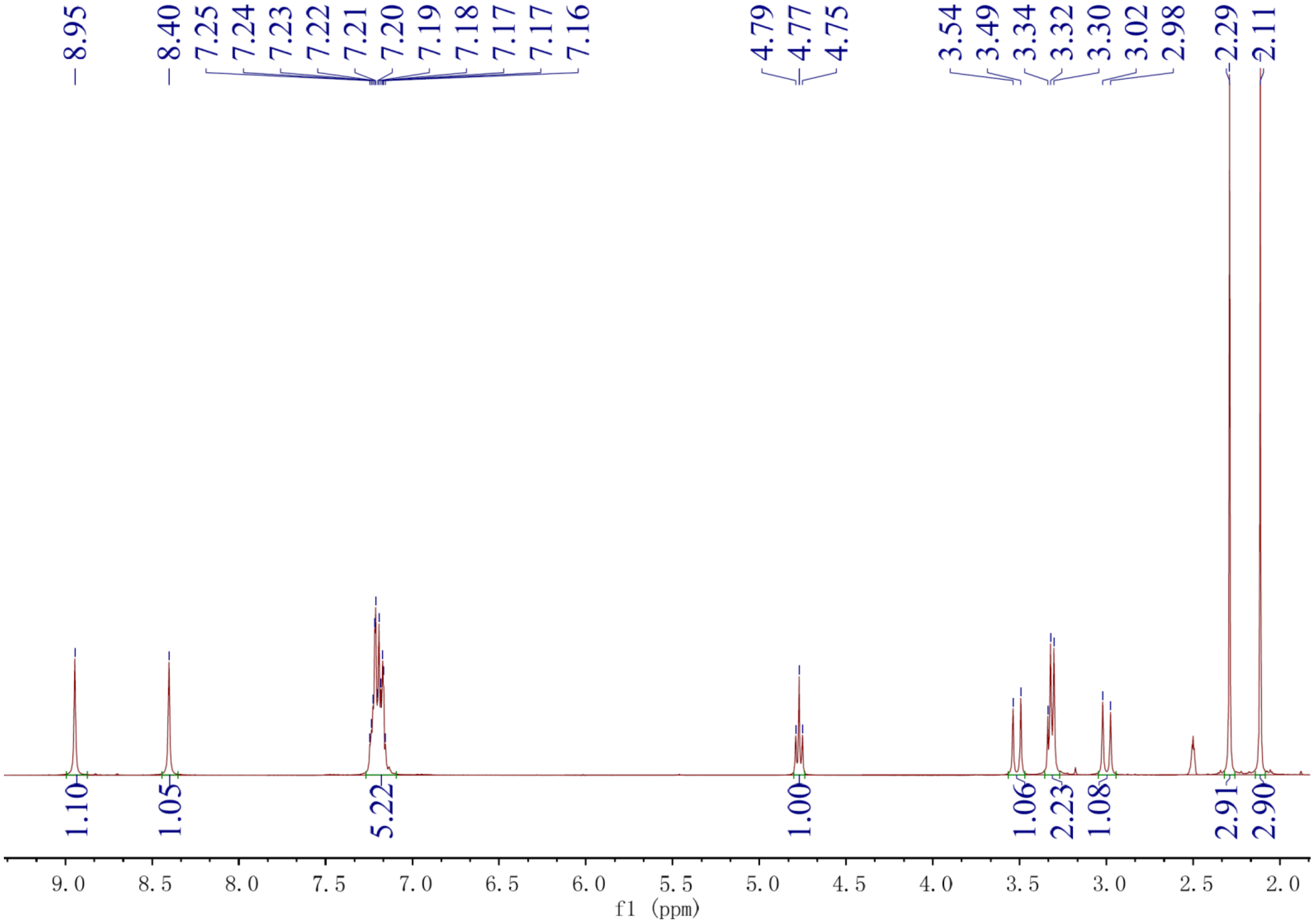

Figure S60. ^{13}C NMR (75 MHz, DMSO-*d*6) spectrum of bis-N-norgliovictin (**13**).

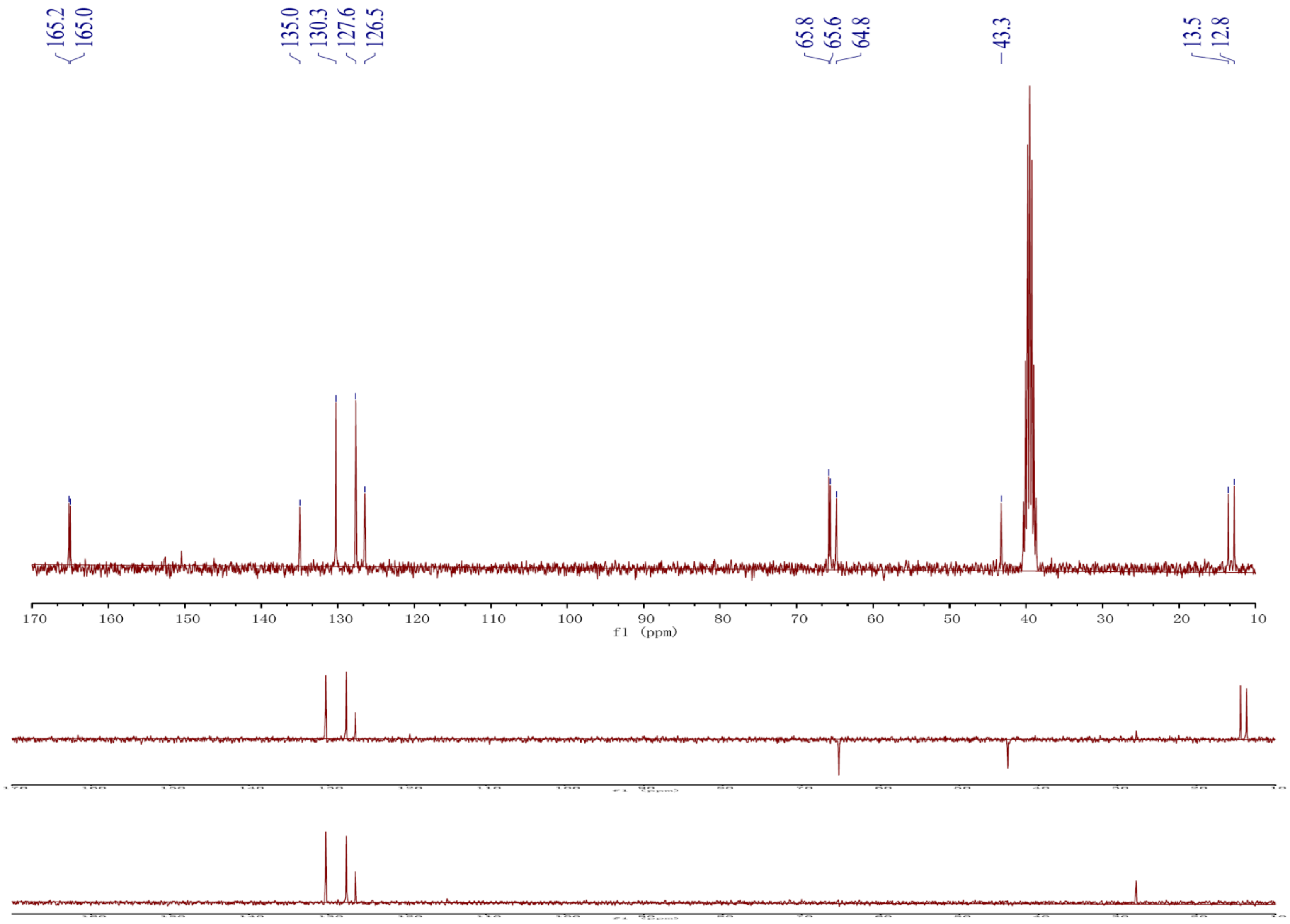

Figure S61. LREIMS of neosartin C (**14**).

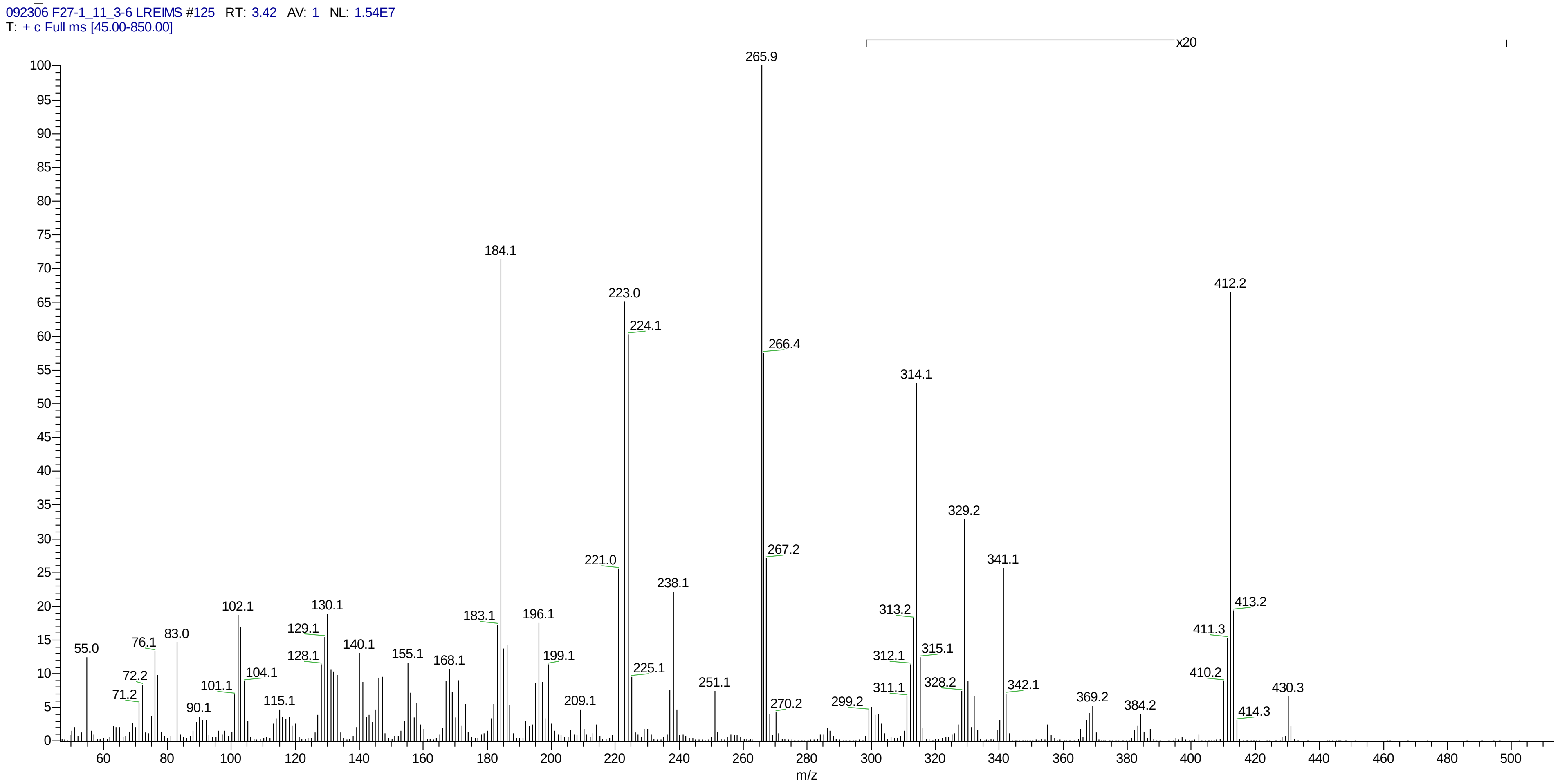

Figure S62. HREIMS of neosartin C (**14**).

SPECTRUM - MS
File : D:\DATA-HR\13\092901-f27-c1.RAW
Full ms [414.500 - 433.500] - Range: 430.100 - 430.200
Scan No. 18 of 24
Scan #: 18
RT: 0.67
Data points: 1

Mass	Relative Intensity	Theoretical Mass	Delta[ppm]	Delta[mmu]	RDB	Composition
430.16354	62.7	430.16356	-0.0	-0.0	16.0	$C_{24}H_{22}O_4N_4$

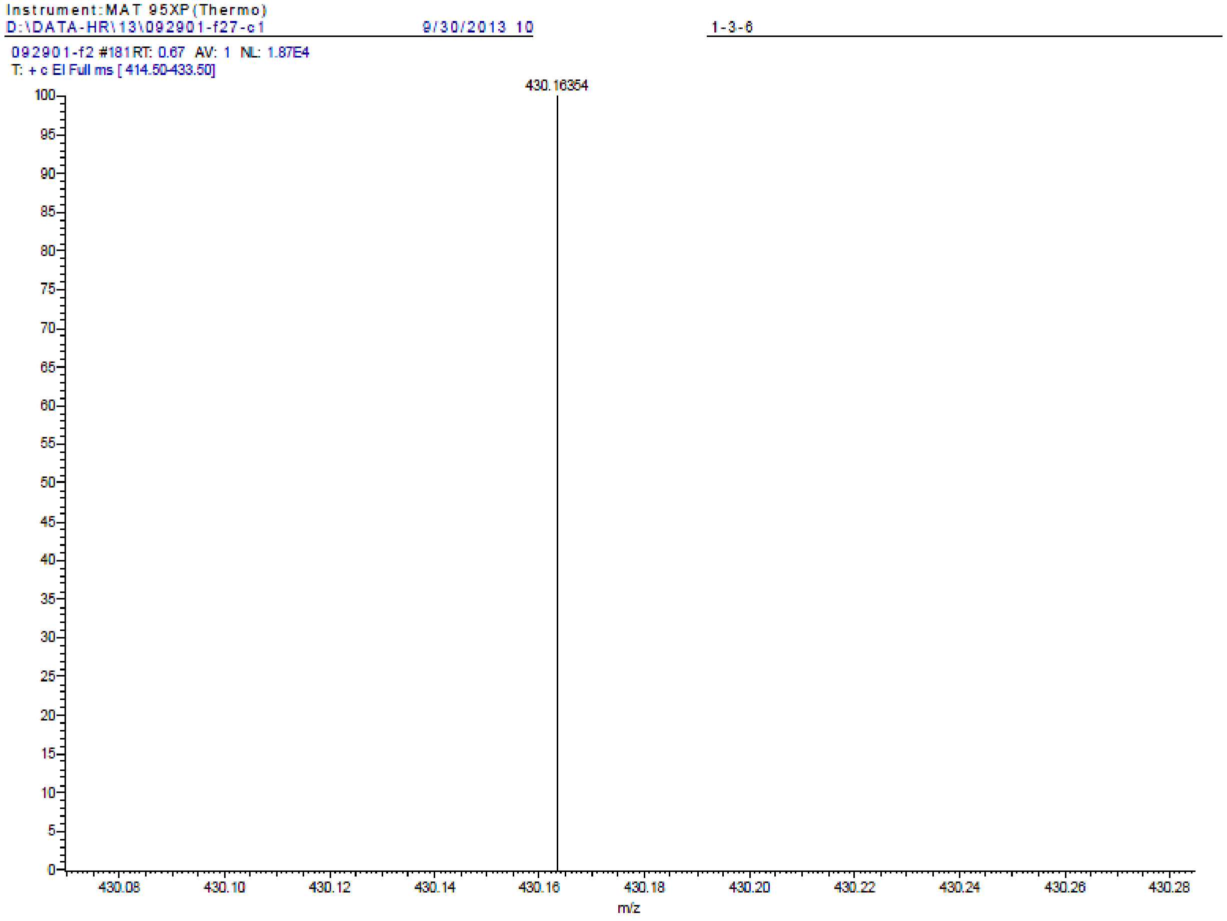

Figure S63. ^{1}H NMR (400 MHz, $CDCl_3$) spectrum of neosartin C (**14**).

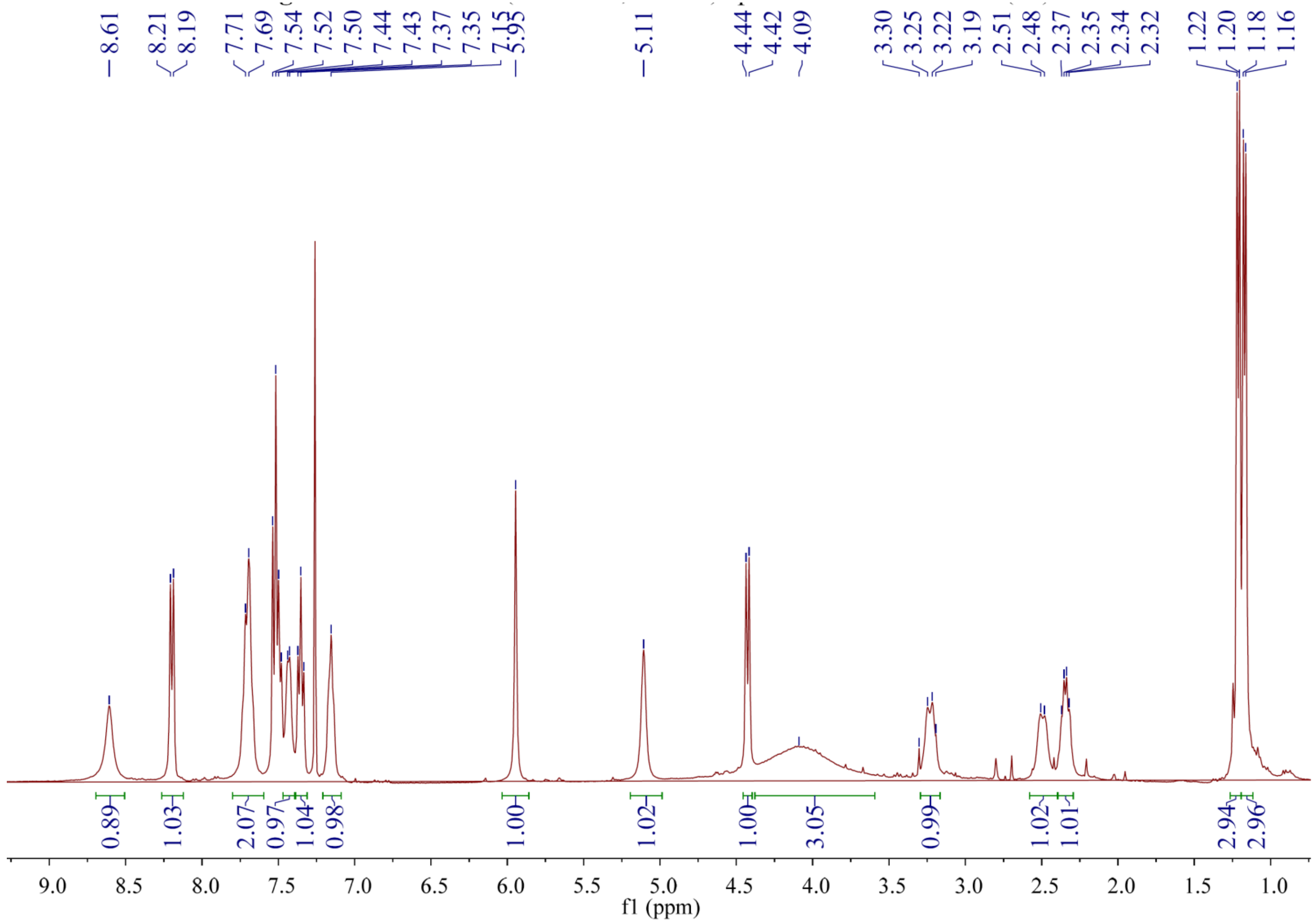

Figure S64. ^{13}C NMR (100 MHz, $CDCl_3$) spectrum of neosartin C (**14**).

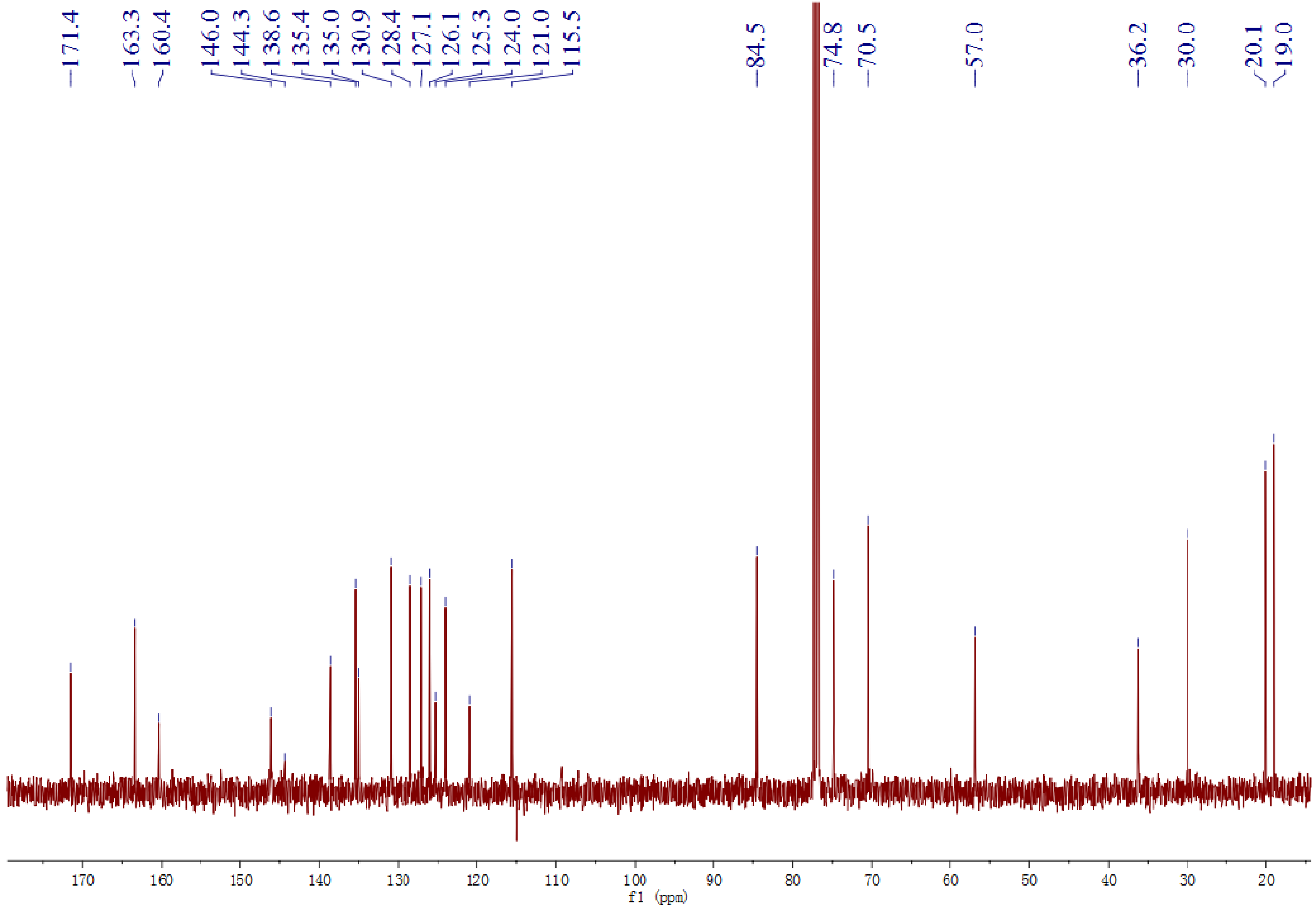

Figure S65. gHMQC spectrum of neosartin C (**14**).

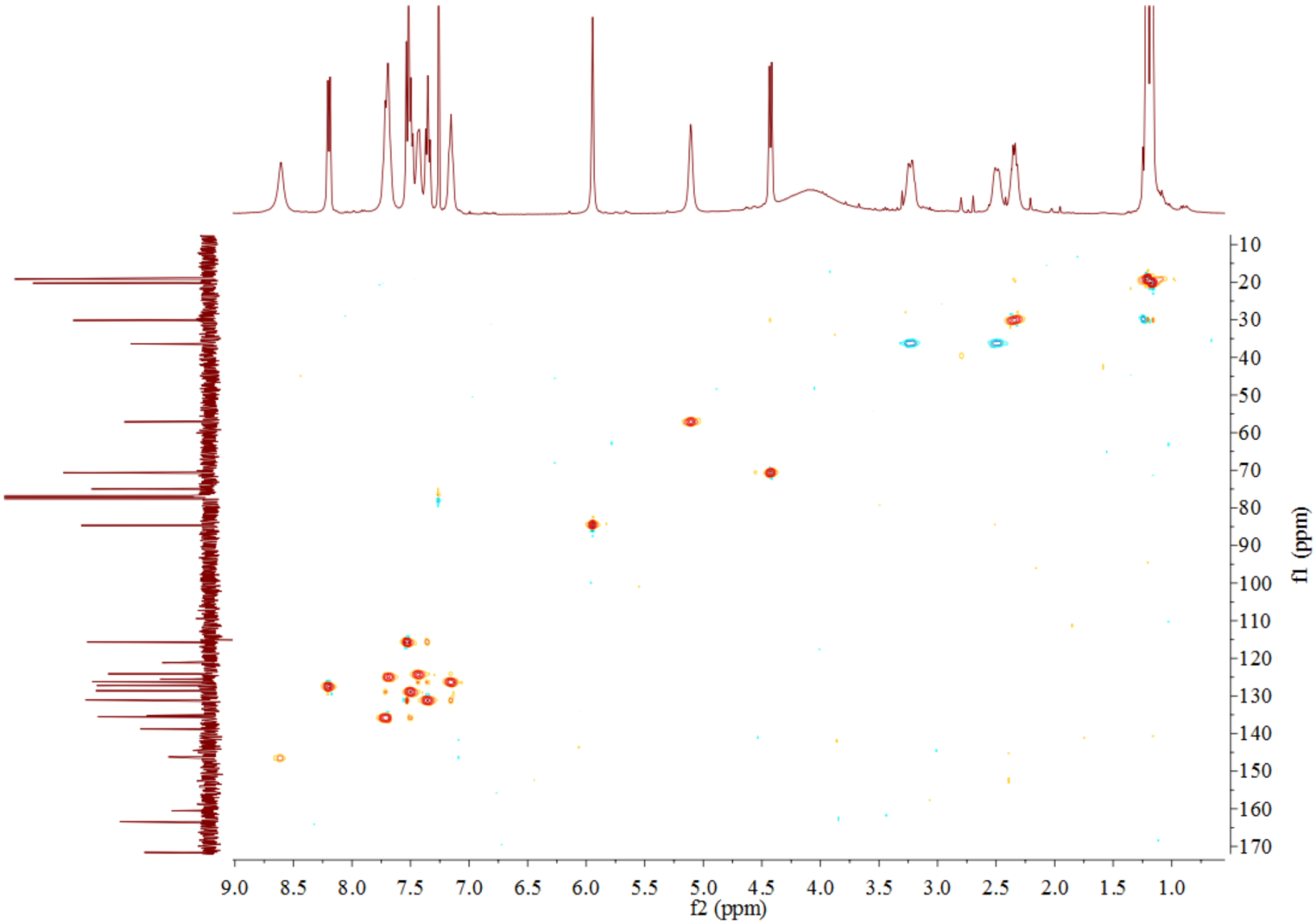

Figure S66. ^{1}H-^{1}H gCOSY spectrum of neosartin C (**14**).

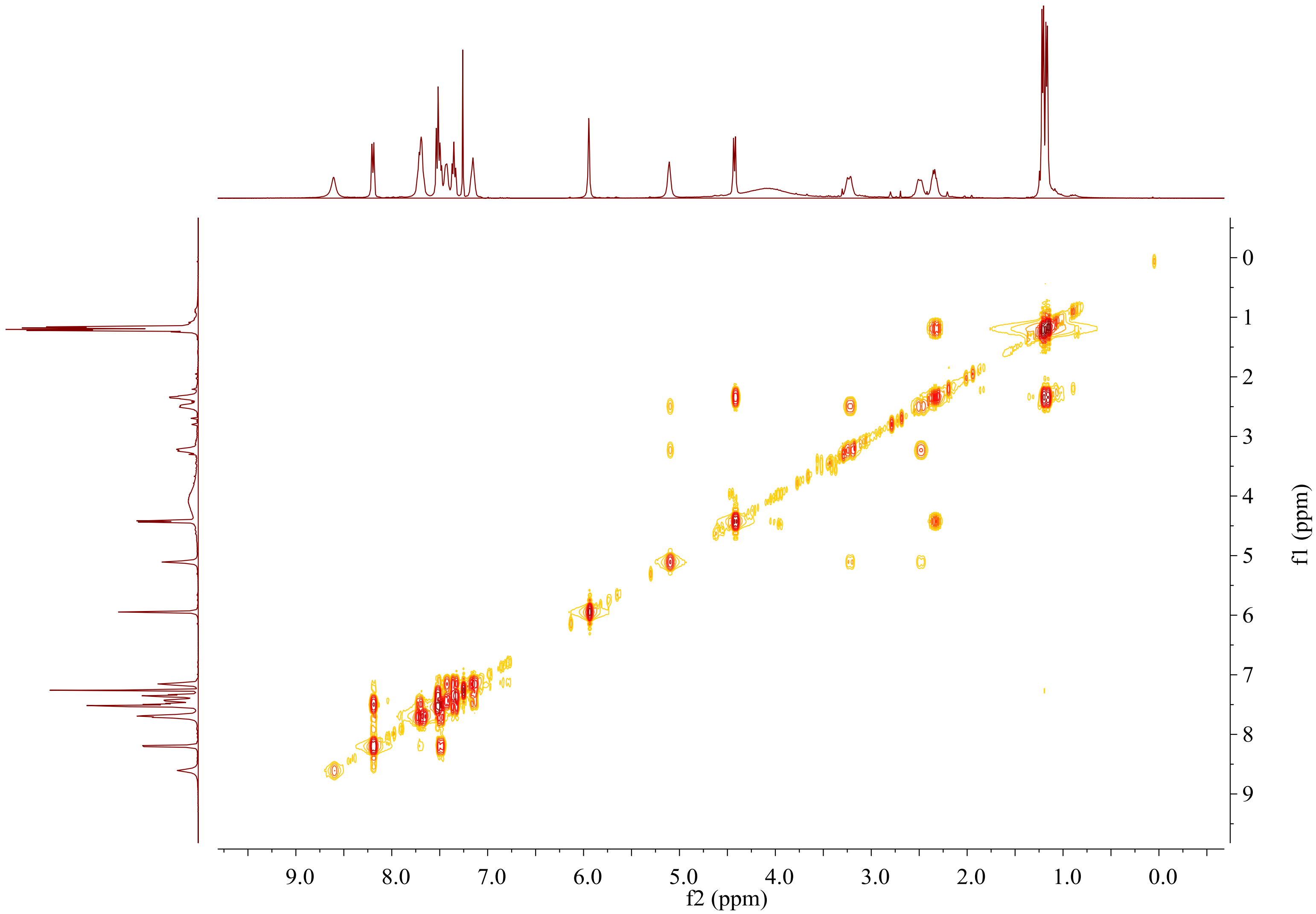

Figure S67. gHMBC spectrum of neosartin C (**14**).

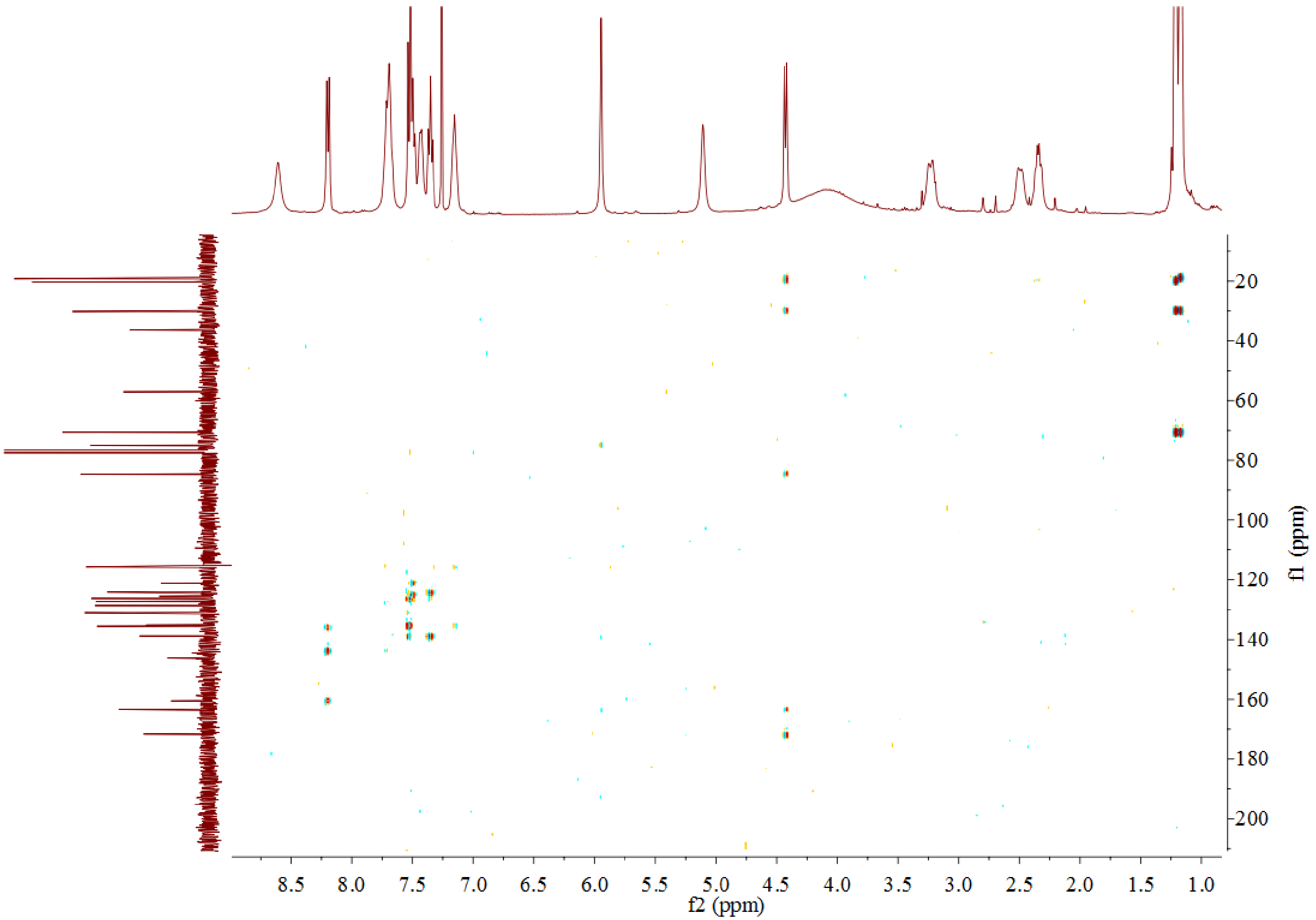

Figure S68. NOESY of neosartin C (**14**).

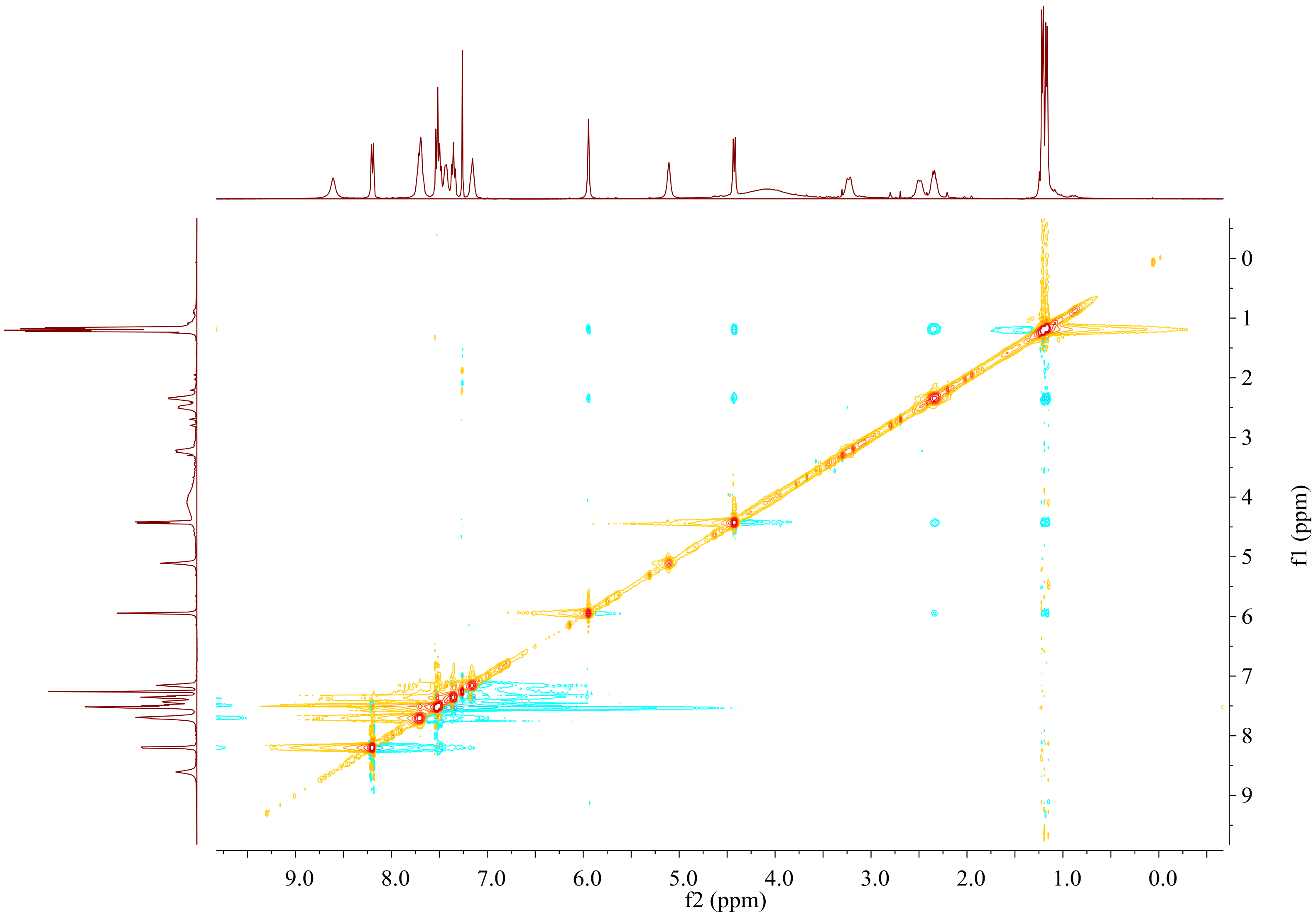

Figure S69. LREIMS of pyripyropene A (**15**).

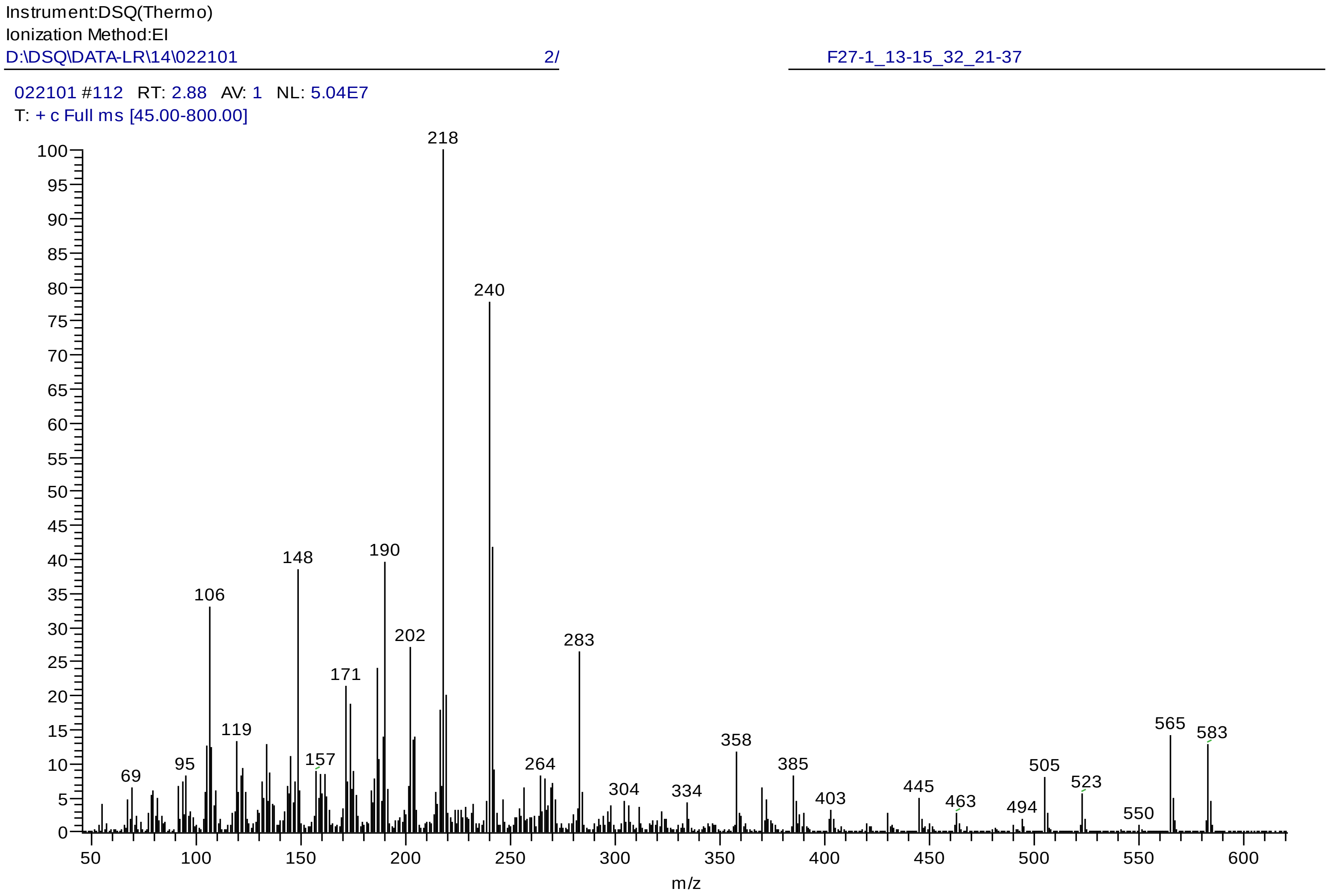

Figure S70. ^{1}H NMR (400 MHz, $CDCl_3$) spectrum of pyripyropene A (**15**).

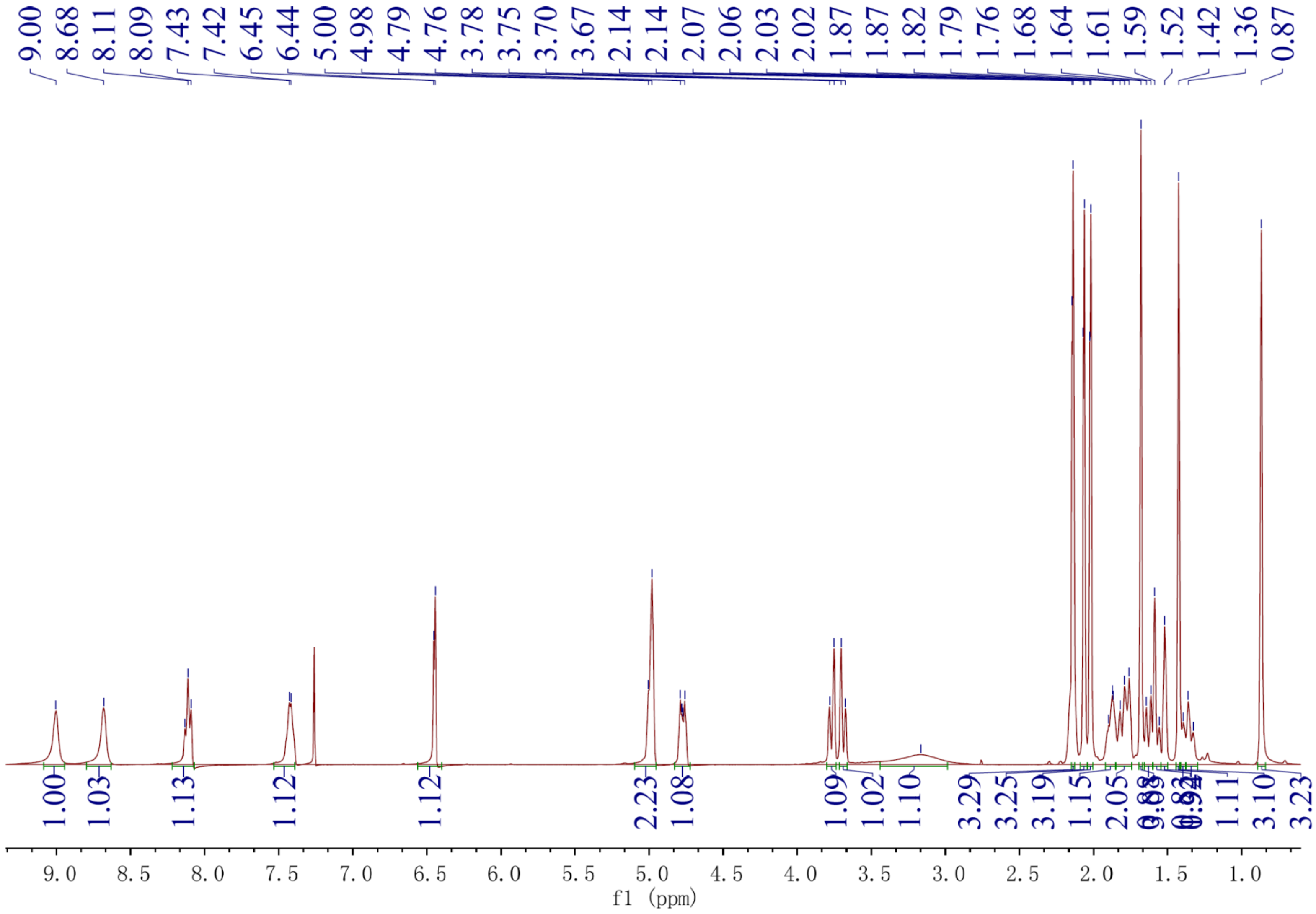

Figure S71. ^{13}C NMR (100 MHz, $CDCl_3$) spectrum of pyripyropene A (**15**).

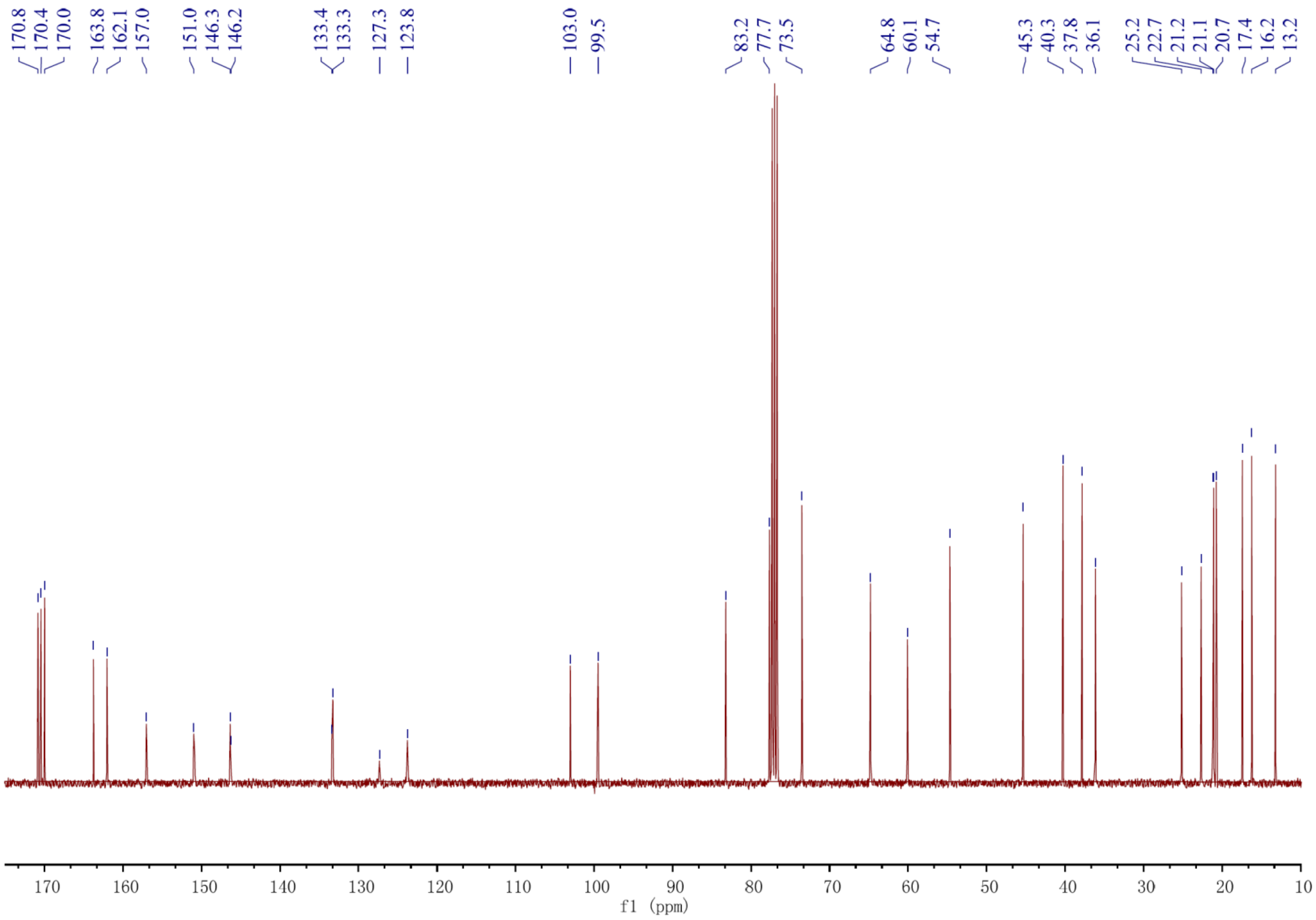

Figure S72. gHMQC of pyripyropene A (**15**).

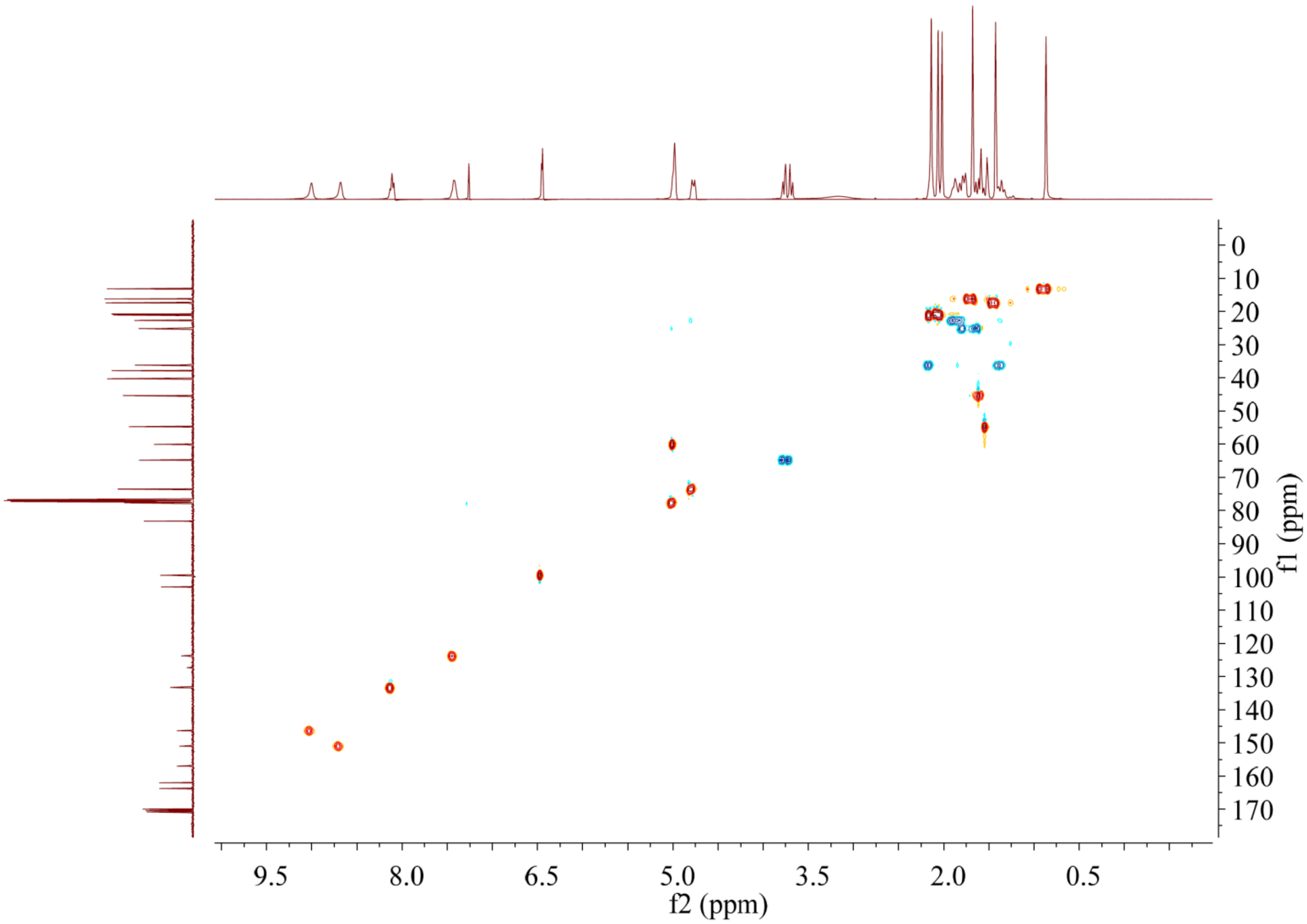

Figure S73. ^{1}H-^{1}H gCOSY of pyripyropene A (**15**).

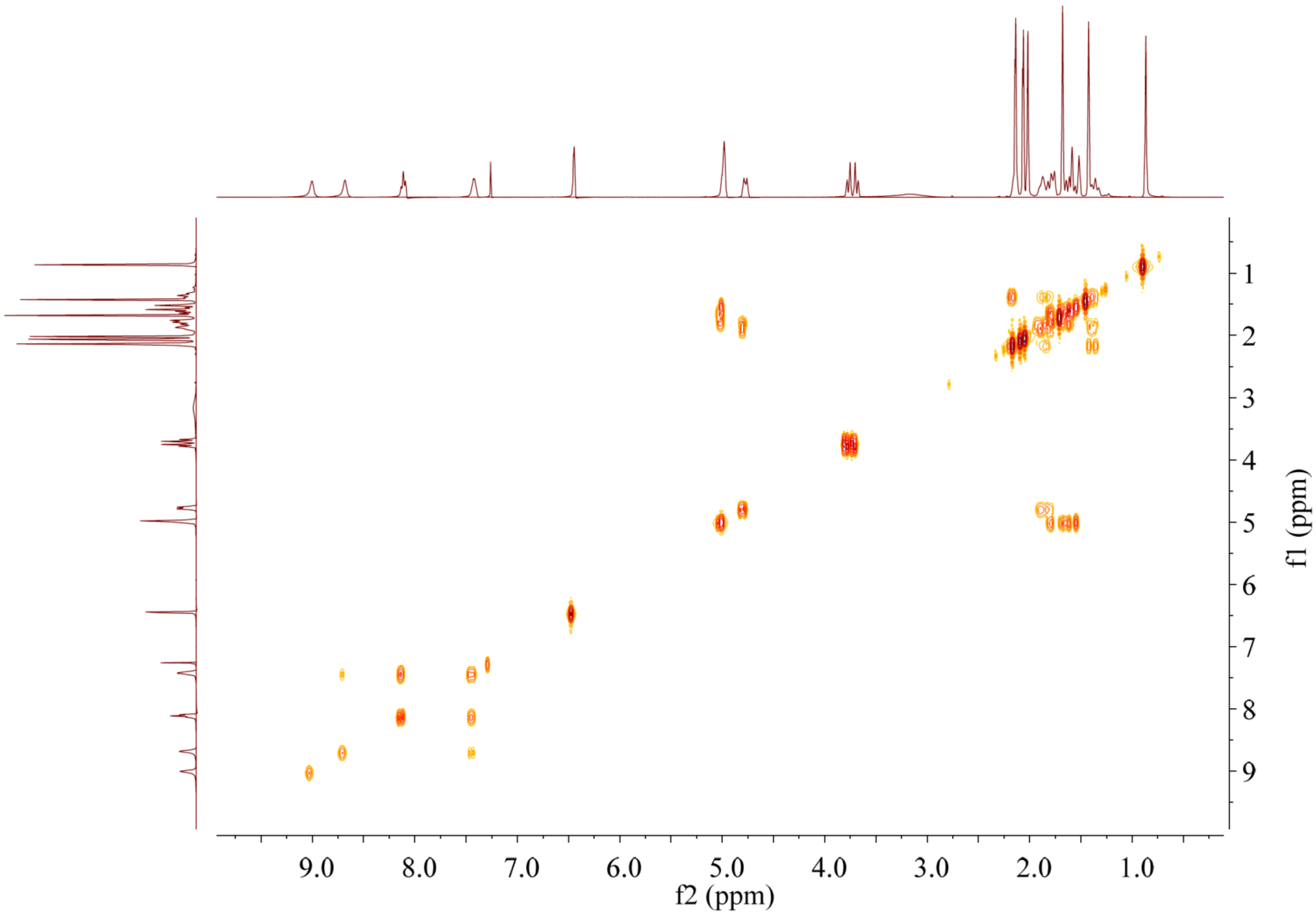

Figure S74. gHMBC of pyripyropene A (**15**).

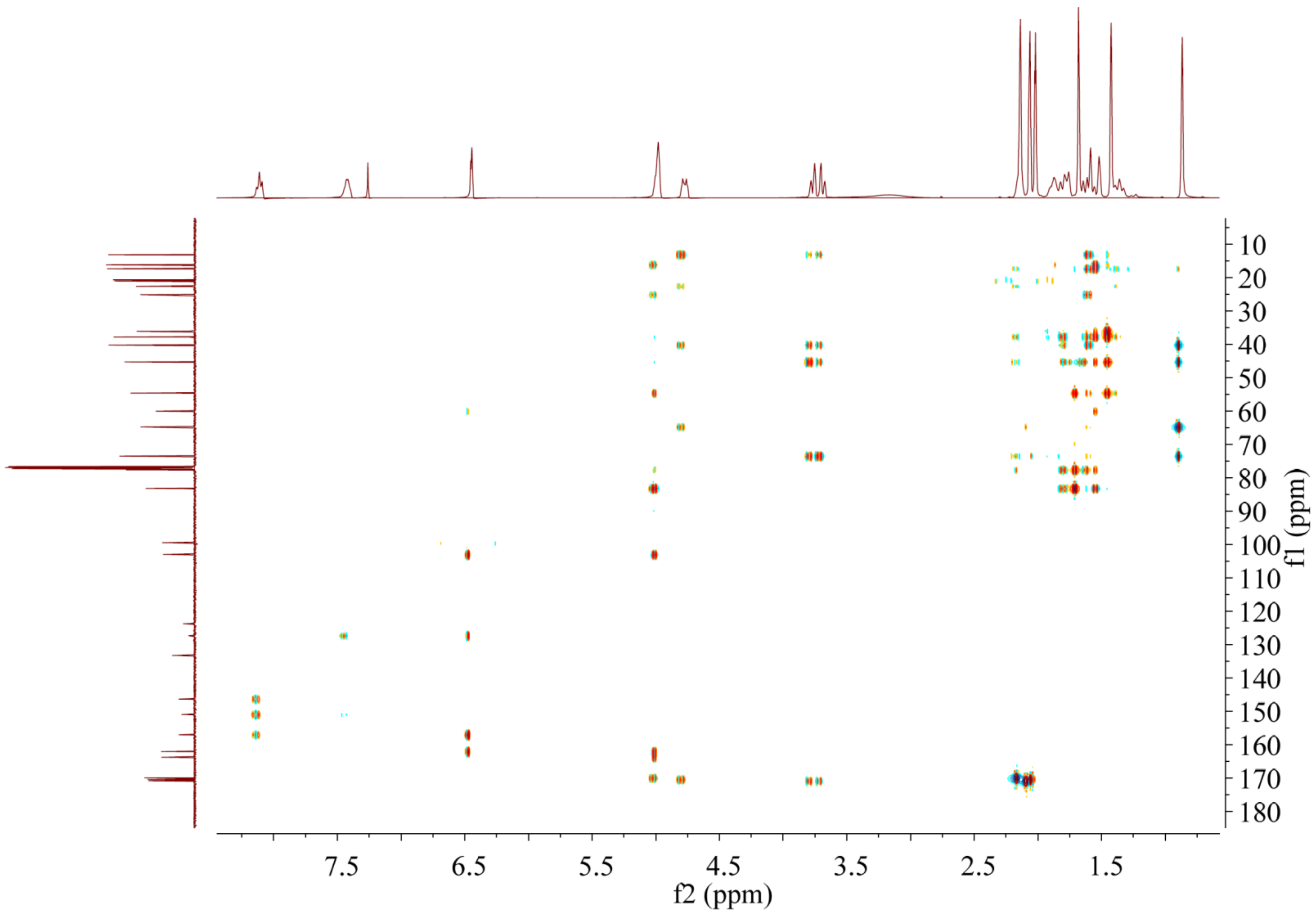

Figure S75. NOESY of pyripyropene A (**15**).

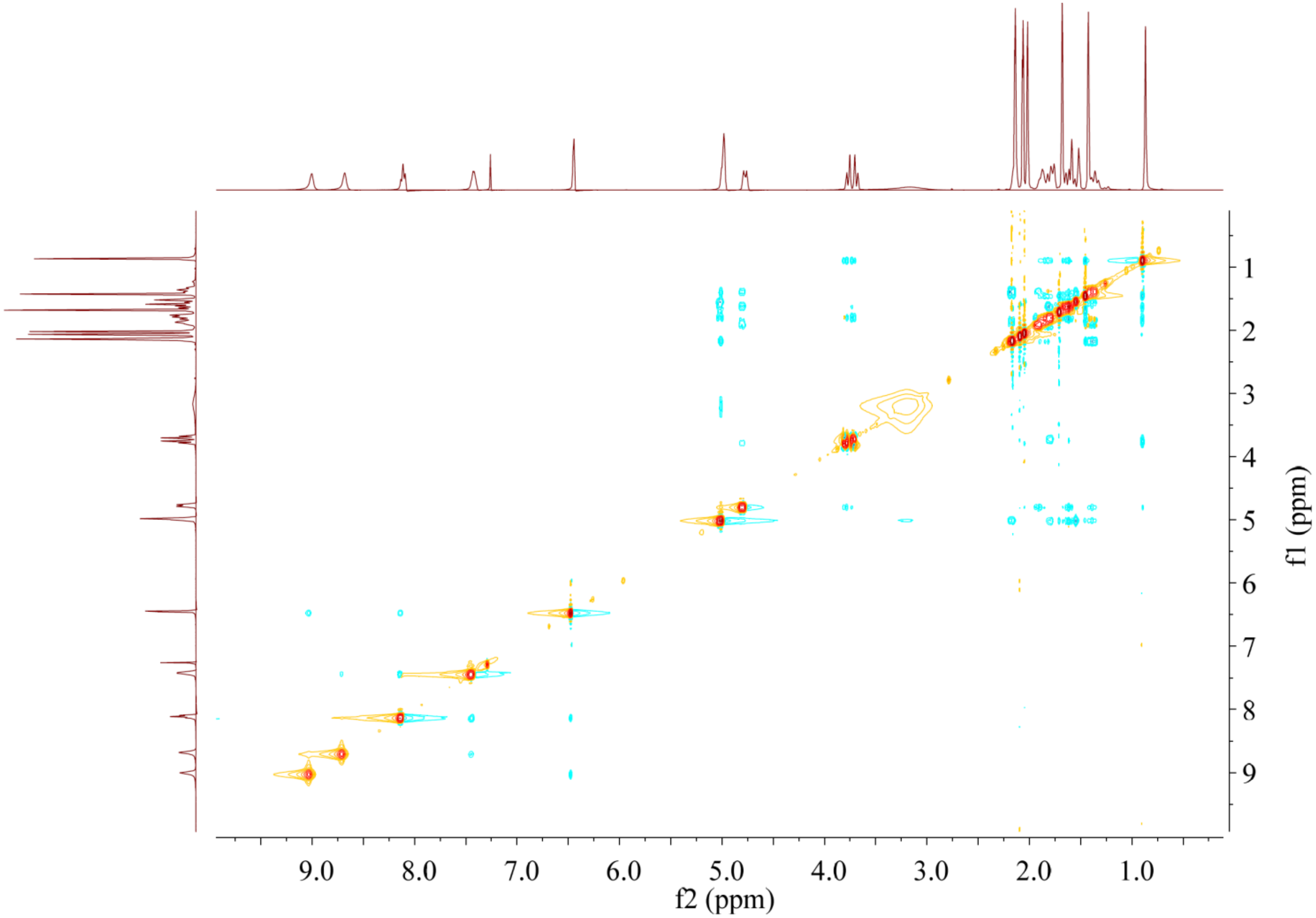

© 2014 by the authors; licensee MDPI, Basel, Switzerland. This article is an open access article distributed under the terms and conditions of the Creative Commons Attribution license (http://creativecommons.org/licenses/by/4.0/).
